# Effect of Water Networks On Ligand Binding: Computational Predictions vs Experiments

**DOI:** 10.1021/acs.jcim.4c01291

**Published:** 2024-11-22

**Authors:** Tibor Viktor Szalai, Dávid Bajusz, Rita Börzsei, Balázs Zoltán Zsidó, Janez Ilaš, György G. Ferenczy, Csaba Hetényi, György M. Keserű

**Affiliations:** 1 Medicinal Chemistry Research Group, Drug Innovation Centre, HUN-REN Research Centre for Natural Sciences, Magyar tudósok krt. 2, Budapest 1117, Hungary; 2 Department of Inorganic and Analytical Chemistry, Faculty of Chemical Technology and Biotechnology, Budapest University of Technology and Economics, Műegyetem rkp. 3, Budapest H-1111, Hungary; 3 National Drug Research and Development Laboratory, Magyar tudósok krt. 2, Budapest 1117, Hungary; 4 Pharmacoinformatics Unit, Department of Pharmacology and Pharmacotherapy, Medical School, University of Pécs, Szigeti út 12, Pécs H-7624, Hungary; 5 Department of Pharmaceutical Chemistry, Faculty of Pharmacy, University of Ljubljana, Aškerčeva cesta 7, Ljubljana 1000, Slovenia; 6 Department of Organic Chemistry and Technology, Faculty of Chemical Technology and Biotechnology, Budapest University of Technology and Economics, Műegyetem rkp. 3, Budapest H-1111, Hungary

## Abstract

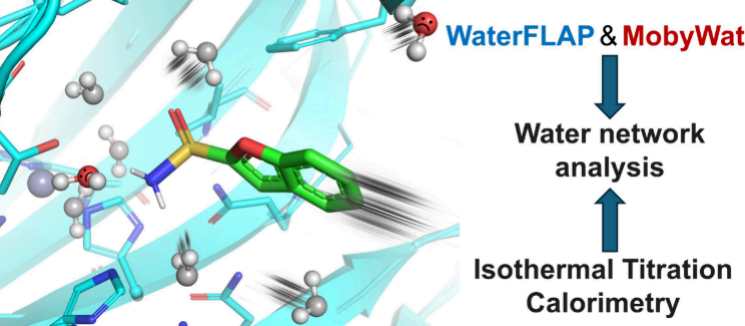

Rational drug design focuses on the explanation and prediction of complex formation between therapeutic targets and small-molecule ligands. As a third and often overlooked interacting partner, water molecules play a critical role in the thermodynamics of protein–ligand binding, impacting both the entropy and enthalpy components of the binding free energy and by extension, on-target affinity and bioactivity. The community has realized the importance of binding site waters, as evidenced by the number of computational tools to predict the structure and thermodynamics of their networks. However, quantitative experimental characterization of relevant protein–ligand–water systems, and consequently the validation of these modeling methods, remains challenging. Here, we investigated the impact of solvent exchange from light (H_2_O) to heavy water (D_2_O) to provide complete thermodynamic profiling of these ternary systems. Utilizing the solvent isotope effects, we gain a deeper understanding of the energetic contributions of various components. Specifically, we conducted isothermal titration calorimetry experiments on trypsin with a series of *p*-substituted benzamidines, as well as carbonic anhydrase II (CAII) with a series of aromatic sulfonamides. Significant differences in binding enthalpies found between light vs heavy water indicate a substantial role of the binding site water network in protein–ligand binding. Next, we challenged two conceptually distinct modeling methods, the grid-based WaterFLAP and the molecular dynamics-based MobyWat, by predicting and scoring relevant water networks. The predicted water positions accurately reproduce those in available high-resolution X-ray and neutron diffraction structures of the relevant protein–ligand complexes. Estimated energetic contributions of the identified water networks were corroborated by the experimental thermodynamics data. Besides providing a direct validation for the predictive power of these methods, our findings confirmed the importance of considering binding site water networks in computational ligand design.

## Introduction

Molecular recognition of ligands by relevant drug targets is a fundamental process in drug design, and understanding the underlying thermodynamics is essential for the accurate prediction of binding affinities and optimizing drug candidates.^1,2^ The early model of Emil Fischer emphasizes the shape complementarity of the enzyme binding pocket (lock) and the ligand (key).^3^ This model captures important aspects of ligand–protein binding; however, many other factors modulating ligand affinity have since been revealed. While shape complementarity of apolar moieties confers modest sensitivity to the orientation of the interacting atoms, directional interactions, like hydrogen bonds and multipole–multipole interactions have a steep dependence on atomic positions and they are optimal in a narrow range of geometrical parameters.^4,5^ When ligands bind in a physiological environment, both the ligand and the protein are solvated prior to binding and the binding process includes at least a partial desolvation of both components. The distinction between apolar and polar moieties is crucial when concerning desolvation and the replacement of the ligand–water and protein–water interactions by ligand–protein and water–water interactions.^6−9^ The contribution of desolvation to the binding free energy is often significant, or even dominant when large, apolar ligands bind to apolar binding sites.^10,11^ While the size of the apolar surface that is buried during binding often correlates with the binding free energy,^12^ a more detailed description, accounting for the position and interactions of water molecules before and after ligand binding, is often needed for the interpretation of structure–activity relationships and a quantitative account of ligand affinity.^13−17^ However, experimental information on water positions is sparse since water molecules are typically invisible by X-ray crystallography, unless high resolution is achieved. Neutron crystallography is more amenable to locate water molecules, including H atom positions, but its application is less abundant owing to associated technical challenges.^18,19^ Even when crystallographic techniques provide information on the position of (selected) water molecules, it is not straightforward to identify their contribution to the binding free energy. However, the net thermodynamics of water networks can be characterized by isothermal titration calorimetry (ITC) applied to the same ligand–protein system both in light and heavy water. Solvation is generally enthalpically more favorable in heavy water compared to regular water.^20^ This observation is often associated with the approximately 10% stronger hydrogen bonds in deuterium oxide than in regular water and with the water reorganization upon ligand–protein binding.^21−24^ However, a detailed understanding of the phenomenon requires the analysis of the intra- and intermolecular H-bonds among all constituents, namely, the ligand, protein, and water, and there are indications that solvation enthalpy differences in light and heavy water depend on the solute properties and vary between hydrophobic and hydrophilic solutes^24,25^ and between positively and negatively charged groups.^26^ Moreover, replacing H_2_O with D_2_O can in some cases affect the structure of the solutes.^25^ Altogether, the more favorable enthalpy of solvation in heavy water compared to light water is a frequent observation, although its details are not fully understood. Since ligand–protein binding in water is accompanied by desolvation, the measure of the binding enthalpy difference in heavy versus regular water indicates the perturbation of the water network. Therefore, by replacing regular water with heavy water, we can probe the role of water molecules in the binding energetics without directly modifying the ligand–protein interactions. While ITC is widely used for the thermodynamic characterization of ligand–protein binding (see reviews, e.g., in refs (27 and 28)), its application to measure binding enthalpies in heavy water is less common, although the reorganization of the water network was investigated for various systems including carbohydrate–protein,^29−32^ DNA–protein,^33^ and ligand–protein^20,34,35^ complexes. An alternative approach to explore the role of water in protein–ligand binding is to apply computational tools developed for the structural and energetic characterization of water molecules in a protein pocket before and after ligand binding.^36−40^ These tools typically assign approximate enthalpy or free energy contributions to each water molecule and aim to provide a quantitative structural and energetic description of their apo and ligand-bound network within a protein pocket. Although the computational characterization of water networks has become an integral part of the design protocols, quantitative assessment of their predictions against experimental binding thermodynamic data is still missing.

In the present study, we jointly apply the above methods to analyze the role of water network perturbation in ligand binding. We combine data from experimental techniques, namely, water positions from high-resolution crystallography and enthalpy of water network reorganization upon ligand binding from ITC measurements in light and heavy water. Experimental characterization of water positions and energetics was compared with the results of computational approaches that generate both positions and binding free energies for a network of water molecules.

Two protein–ligand systems were selected for investigation: trypsin with a series of *p*-substituted benzamidines and carbonic anhydrase II (CAII) with a series of aromatic sulfonamides.^41,42^ These systems were chosen based on their reported sensitivity to water network effects,^43−45^ but in addition to being popular model systems in biochemical and drug design efforts, both proteins are relevant drug targets in oncology: trypsin overexpression is connected to colorectal cancer,^46^ while CAII upregulation is connected to tumor progression and metastasis.^47^

Experimental water positions in apo and complex protein structures were taken from published high-resolution X-ray and neutron diffraction data. We performed ITC experiments in light and heavy water to obtain direct quantitative information on the binding enthalpy and the affinity, allowing us to evaluate the changes associated with the water network rearrangement in both the enthalpy and entropy terms. We selected two orthogonal computational methods for predicting water positions and energetics and to compare them with experimental results.

WaterFLAP, included in the FLAP 2.2 software package,^39,48^ is a knowledge-based approach that uses GRID molecular interaction fields (MIFs)^49^ to classify the water molecules into structural, displaceable, or bulk characters. WaterFLAP has been successfully used previously to generate water networks for G protein-coupled receptors (GPCRs), to provide an understanding of structure–activity relationships and showing how the displacement of “unhappy” (high-energy) water molecules provides a key binding energy component to assist structure-based drug design.^2^ As a grid-based method, a key advantage of WaterFLAP against molecular dynamics-based (MD-based) approaches is higher speed/lower computational demand.^50^ On the other hand, WaterFLAP showed a reduced accuracy in water placement compared to the MD-based WaterMap.^51^ The highlighted problem was the prediction of the energetic contributions of water molecules. The water scoring method of WaterFLAP also highly depends on the number of hydrogen bonds of the water network.^51^ Additionally, as the characterization of water molecules relies on energy cutoffs, WaterFLAP may miss biologically significant water molecules if they fall outside the predefined energy ranges.

The other applied water prediction method, MobyWat,^38,52^ uses a different principle, as it predicts and analyses the individual hydrating water positions based on occupancy and mobility values gained from MD trajectories. It predicts void-free hydrated structures including molecular surfaces and interfaces of complexes. The mobility of the predicted water molecules and protein–water and water–water interactions is followed by molecular dynamics to explore the static and dynamic hydration networks and identify the crucial (nonmobile) water molecules in ligand binding. MobyWat was tested on numerous proteins, including complexes with ligands of various sizes.^38,52,53^ The water molecule position predicting ability of MobyWat was validated on more than 1500 high-quality crystallographic water oxygen positions on which it achieved an overall more than 80% success rate.^52^ It was also investigated how the accuracy of predicted water positions is influenced by the parameters of MD simulations including temperature, pressure, and the applied force field and explicit water model.^54^ From the above-mentioned MD parameters, temperature showed the highest impact on the efficiency and reproducibility of the predicted hydration structure.^54^ To note, WaterMap also applies an MD-based approach for generating water positions. Importantly, MobyWat is an open-source software, while WaterMap is commercial. MobyWat implements a solution for the calculation of the hydration structure of the entire (unliganded) target protein surface and also for the target–ligand interface, along with various clustering and analysis schemes, and an extensive validation mode. Additionally, it can be used with any MD software, as it reads common file formats like the text-based PDB structure files or the portable binary xdr (xtc) trajectory files.

The results obtained from this study are expected to contribute to a deeper understanding of the thermodynamics of protein–ligand binding and the specific role of the water networks in a wider sense. By identifying cases where water molecules have a pronounced impact on the binding energetics, we can refine computational models and enhance the accuracy of binding affinity predictions. This, in turn, has the potential to facilitate the identification of more effective ligands by computer-aided drug design.

## Materials and Methods

### Materials

Bovine pancreatic trypsin (trypsin from bovine pancreas TPCK treated, essentially salt-free, lyophilized powder, ≥10,000 BAEE units/mg protein, CAS: 9002-07-7, cat. no. T1426) and CAII from bovine erythrocytes (lyophilized powder, ≥3,000 W-A units/mg protein, CAS: 9001-03-0, cat. no. C2522) were acquired from Sigma-Aldrich. 4-Amidinobenzamide hydrochloride (**4-CONH2-BA**, CAS: 59855-11-7, cat. no. OR-2209), 4-methoxybenzenecarboximidamide hydrochloride (**4-OCH3-BA**, CAS: 51721-68-7, cat. no. QB-3215), benzamidine hydrochloride (**BA**, CAS: 1670-14-0, cat. no. OR-0201), 4-aminobenzamidine hydrochloride (**4-NH2-BA**, CAS: 2498-50-2, cat. no. OR-2207), thiophene-2-sulfonamide (**TP-2-SA**, CAS: 6339-87-3, cat. no. OR-1553), 2-fluorobenzenesulfonamide (**2-F-SA**, CAS: 30058-40-3, cat. no. QA-4700), and sulfanilamide (**4-NH2-SA**, CAS: 63-74-1, cat. no. AN-2948) were obtained from Combi-Blocks; 1-benzofuran-2-sulfonamide (**BF-2-SA**, CAS: 124043-72-7, cat. no. EN300-279329), thiazole-2-sulfonamide (**TA-2-SA**, CAS: 113411-24-8, cat. no. EN300-107045), 1,3-benzothiazole-2-sulfonamide (**BTA-2-SA**, CAS: 433-17-0, cat. no. EN300-60766), 4-methylbenzenesulfonamide (**4-CH3-SA**, CAS: 70-55-3, cat. no. EN300-15721), 1*H*-1,3-benzodiazole-2-sulfonamide (**BDA-2-SA**, CAS: 5435-31-4, cat. no. EN300-61671), and 4-methyl-benzene-1-carboximidamide hydrochloride (**4-CH3-BA**, CAS: 6326-27-8, cat. no. EN300-73561) were obtained from Enamine; 4,5,6,7-tetrahydro-1-benzothiophene-2-sulfonamide (**THBT-2-SA**, CAS: 142294-64-2, cat. no. AS-8962) was obtained from Key Organics/BIONET.

For the CAII measurements, a 100 mM phosphate buffer was prepared using Na_2_HPO_4_·2H_2_O (from Sigma-Aldrich, cat. no. 1.37036) and Milli-Q water, and its pH was set to 7.6 using HCl. For the heavy water measurements, the buffer was lyophilized, and then, the solid residue was dissolved with an appropriate amount of D_2_O to get a 100 mM phosphate buffer solution. The regular water buffer was stored at 4 °C, while the heavy water buffer was aliquoted into 15 mL batches and stored at −20 °C to avoid isotope exchange with air moisture.

A buffer solution for the measurements with trypsin was prepared using 50 mM tris(hydroxymethyl)aminomethane (Tris, from Sigma-Aldrich, cat. no. 10812846001), 100 mM CaCl_2_ (from Sigma-Aldrich, cat. no. 223506), and 0.1 V/V % of PEG-600 (from Sigma-Aldrich, cat. no. 8.07486), and its pH was set to 7.8 using HCl. For the heavy water measurements, the buffer was lyophilized, and then, the solid residue was dissolved with an appropriate amount of D_2_O to get a 50 mM Tris buffer solution. The regular water buffer was stored at 4 °C, while the heavy water buffer was aliquoted in 15 mL batches and stored at −20 °C to avoid isotope exchange with air moisture.

### Isothermal Titration Calorimetry

ITC measurements for CAII were carried out on a MicroCal PEAQ-ITC microcalorimeter (Malvern Instruments, Worcestershire, UK). Protein solutions with a concentration between 50 and 125 μM were prepared in batches freshly prior to measurements, using a buffer warmed up to room temperature. Exact concentrations were verified on a NanoDrop 1000 spectrophotometer by measuring the optical absorbance of the solution at 280 nm using a molar absorption coefficient of 50,410 M^–1^ cm^–1^ with an optical path length of 0.05 cm and a molecular weight of 29 kDa.^55^ Ligand solutions were freshly prepared using the buffer solutions warmed up to room temperature prior to use, in a concentration of either 500 μM or 1 mM, depending on the ligand. The protein solution was loaded into the sample cell in a volume of 200 μL and was titrated at 25 °C with the ligand solution at a stirring speed of 750 rpm. Data were analyzed using the MicroCal PEAQ-ITC analysis software (version 1.22) by fitting a single-site binding curve.

ITC measurements for trypsin were carried out on a General Electric MicroCal VP-ITC device. Protein solutions with a concentration of 150 μM were prepared in batches freshly prior to measurements using a buffer warmed up to room temperature. Ligand solutions were freshly prepared, using the buffer solutions warmed up to room temperature, prior to use with a concentration of 5 mM. The protein solution was loaded into the sample cell with a volume of 1.433 mL and was titrated at 25 °C with the ligand solution at a stirring speed of 307 rpm. Data were analyzed using Origin7 with ITC_200_ plugin by fitting a single-site binding curve.^56^

The change in Gibbs free energy (Δ*G*_bind_) upon protein–ligand binding is expressed as
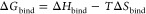
1where Δ*H*_bind_ represents the enthalpy change, *T* represents the temperature, and Δ*S*_bind_ corresponds to the entropy change upon binding. Water molecules within the protein binding site contribute to the overall binding energetics by influencing both the entropy and enthalpy components of the binding free energy. The direct connection between affinity and the Gibbs free energy is described by
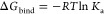
2where *R* represents the universal gas constant and *K*_a_ represents the association constant (and *K*_d_ the respective dissociation constant, the reciprocal of *K*_a_) of protein–ligand binding, expressed as the following equilibrium process:
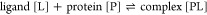

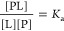
3
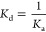
4

The applied injection programs for each measurement, as well as the respective titration curves, are included in the Supporting Information (Tables S7 and S8, ITC results section). For all measurements, the ligand solution was degassed prior to use. Blank measurements consisting of titrating the ligand into the buffer were carried out to correct for the heat of dilution for all ligands, using the appropriate injection program. The blank measurements were used to correct the acquired curves. The parameters acquired directly from the fitting were the stoichiometry of binding, Δ*H*_bind_ (binding enthalpy), and *K*_d_ (dissociation constant),^56^ while the further thermodynamic terms were calculated based on eqs 1, 2, and 4. All ligand measurements were replicated at least twice.

### Water Network Prediction and Scoring with WaterFLAP

WaterFLAP combined with GRID was used to predict waters in the binding site and near the ligands. Protein complex structures were imported from the Protein Data Bank (PDB)^57^ where available: specifically, PDB entries 1S0R (**BA**),^58^3GY4 (**4-NH2-BA**),^59^2GLX,7WA2 (**4-OCH3-BA**),^60^2WEG (**2-F-SA**),^43^6RL9 (**4-NH2-SA**),^61^4YXI (**4-CH3-SA**),^62^3S71 (**BF-2-SA**), 3S72 (**BDA-2-SA**), 3S73 (**BTA-2-SA**), 3S77 (**TA-2-SA**), and 3S78 (**TP-2-SA**) were used.^45^

For ligands lacking a PDB entry (**4-CONH2-BA**, **4-CH3-BA**, and **THBT-2-SA**), the 3GY4 and 3S71 protein structures were preprocessed using the Schrödinger Protein preparation wizard;^63^ then, grid generation was carried out on both proteins using Schrödinger Glide.^64,65^ Centers of the grids were chosen as the centroid of the ligands found in the PDB entries. 3D structures of the ligands were generated using Schrödinger LigPrep.^63^ Glide was used in SP (standard precision) mode to predict the binding poses. Specifically, **4-CONH2-BA** and **4-CH3-BA** were docked into the 3GY4 protein structure, and **THBT-2-SA** was docked into the 3S71 structure. Only one binding mode was chosen from the docking results for each ligand, where the amidine or the sulfonamide group was in the consensus location and orientation, as observed for the other ligands of the respective series.

First, the empty (apo) binding sites in 1S0R and 3S78 were filled with water molecules using the “predict waters” function in WaterFLAP with a pocket radius of 10 Å; then, the predicted waters were refined with the centered shift optimization method within a pocket radius of 9 Å using a CRY hydrophobic probe and a final GRID energy of −1.0 kcal/mol. Waters were scored after the refining step. Throughout this work, we adapt the phrasing of WaterFLAP to refer to thermodynamically favorable (“happy”, i.e., >2 kcal/mol more stable than bulk) and unfavorable (“unhappy”, i.e., >1.5 kcal/mol less stable than bulk) water molecules. To clarify, these are to be understood against the base case of having that specific water molecule as part of the bulk solvent phase. Naturally, any water molecule is favored over an empty void, which would incur huge entropic penalties.

To calculate the free energy change of the water network (Δ*G*_waters_), water molecules were predicted in the binding site; then, water perturbation analysis was made using the ligand-bound complex structure. The ΔΔ*G*_bind_ corresponds to the change in Δ*G*_bind_ for the (perturbed) water molecule after the ligand binds to the protein and disturbs the water network. These ΔΔ*G*_bind_ values and the Δ*G*_bind_ values of the displaced water molecules were used to calculate the free energy change of the water network, Δ*G*_waters_ (eq 5).^66^
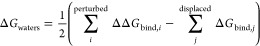
5

In eq 5,  is the sum of the ΔΔ*G*_bind_ values of the water molecules near the binding site after water perturbation analysis,  is the sum of the Δ*G*_bind_ values of the displaced water molecules upon ligand binding,  is the correction factor accounting for double counting the interactions. Note that, in order to calculate the Δ*G*_bind,*i*_ values, water molecules of the apo vs ligand-bound structures should be matched against each other. For simplicity, this was achieved by accounting for the water structure immediately after ligand binding (i.e., neglecting longer-timescale water reorganization upon ligand binding).

### Water Network Prediction with MobyWat

Atomic coordinates of ligand-free (apo) and ligand-bound (holo) trypsin and carbonic anhydrase were accessed at the Protein Data Bank under codes 5MNZ (apo trypsin) and 5MO0 (trypsin-**BA**),^67^7WA2 (trypsin-**4-OCH3-BA**) and 3GY4 (trypsin-**4-NH2-BA**), and 3GZ0 (apo anhydrase),^68^ respectively. To complete missing residues and atoms, homology modeling was used at the Swiss-Model web server.^69^ Water molecules were removed. The protein was capped with an −NHMe group at the C-terminal end in PyMol.^70^ The prepared apo structure was used for subsequent calculations. From the holo structure, only the ligand position was used to define the binding site. Experimental water molecules were only used for validation. In the case of alternative locations for water molecules (as a result of ambiguous structural refinement or a conformational ensemble/equilibrium that exists under the crystallization conditions), location A, i.e., the coordinate set with the highest occupancy was kept over B.

Method 3 from MobyWat^52^ was used for the prediction of the hydration structure, first starting from the previously prepared water-free apo target. For interface water prediction, the ligand from the holo complex was superimposed into the apo target. The exact parameters of minimization and molecular dynamics are described in the “Water Network Prediction with MobyWat” section and in ref (52). Surface water molecules within 5 Å of the superimposed ligand were energy-minimized with the apo target using the previously described steepest descent and conjugate gradient minimization protocol repeated twice. The minimized surface waters with the apo target were used for individual water binding enthalpy calculation.

The validation mode of MobyWat was used for success rate calculation of predicted surface and interface water molecules. The success rate quantifies the match between predicted and experimental (reference) water positions. The match tolerance was set to 1.5 Å. Under a 1.5 Å distance between a predicted and an experimental water molecule indicates identified matching pairs. Then, the number of matches is divided by the number or reference water positions and multiplied by 100 to get the success rate in percentage.

### Calculation of Individual Water Molecule Binding Enthalpies

The minimized and hydrated apo target structures from the previous section were used in the Fragmenter web server.^71^ The ligand was superimposed into the apo target to define the binding site. Target atoms within 8 Å from the ligand and water molecules within 5 Å from both the ligand and target were cut out as the binding interface. The ligand was removed from the binding interface, and the resulting cut-out binding interface structure containing the apo target and the optimized surface water molecules were used for binding enthalpy calculation.^72,73^

The optimized and hydrated apo structure from the previous section and the ligand from the holo complex were merged into one file. This file was then subjected to the editing mode of MobyWat to identify the water molecules overlapping with the ligand. The water molecules that are below a 1.75 Å distance from the ligand are considered as overlapping water molecules.

One water molecule, the water-free cut-out target interface from the previous step and individual water molecules inserted into the target were used for binding enthalpy calculations as in ref (71). The net charge of the system was +1. The calculation of one water molecule resulted in the heat of formation (Δ_f_*H*) value Δ_f_*H*(*H_2_O*). The calculation with the water-free target interface resulted in the value Δ_f_*H*(Target). The calculation with individual water molecules inserted back into the target interface resulted in the Δ_f_*H*(Target:H_2_O) values. These values were used in eq 6 to calculate the binding enthalpy (Δ*H*_b_) of individual water molecules according to Hess’ law. Based on their Δ*H*_b_ values, waters were ranked, with the lowest rank indicating the most favorable Δ*H*_b_. Twenty water molecules were included in the calculations.
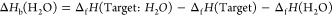
6

## Results and Discussion

For both model systems, we have investigated the protein–ligand binding thermodynamics with isothermal titration calorimetry (ITC). Under isothermal–isobaric conditions, the binding free energy (Δ*G*_bind_) depends on Δ*S*_bind_ and Δ*H*_bind_. The binding entropy Δ*S*_bind_ can be described using three terms:^74^
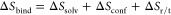
7where Δ*S*_solv_ is the entropy change from the water molecules, as a result of ligand and protein surface burial with accompanying water release upon binding. Δ*S*_conf_ is the entropy change resulting from the changes in the conformational freedom of the protein and the ligand upon binding,^75,76^ and Δ*S*_r/t_ is the entropy change from the loss of translational and rotational degrees of freedom of the protein and ligand upon binding.^77,78^ Δ*S*_solv_ most often has a positive value, Δ*S*_conf_ can be both a negative and positive value, depending on the ligand–protein system, and Δ*S*_r/t_ always has a negative value.^74^ The conformational entropy, Δ*S*_conf_, changes upon ligand binding, and it may, in principle, be affected by the water network and the light to heavy water replacement. However, the small RMSD between the apo and complex X-ray structures (Table S3) suggests that the investigated proteins are rigid and conformational entropy change does not have a major contribution to ligand binding. The vibrational entropy, another part of the configurational entropy, does not appear explicitly in eq 7; however, the effect of a liberating bound water on protein vibrational modes can be modeled,^79^ and water models derived with molecular dynamics may capture at least part of this effect.

Δ*H*_bind_ could be described using two terms:
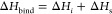
8where Δ*H*_*i*_ is a term that includes the enthalpy change exclusively from the dissolved substances, while Δ*H*_s_ includes all solvent-related enthalpy changes.^21,80^ In this study, we only changed the solvent from H_2_O to D_2_O while keeping every other parameter the same. This means that we should mainly perceive the changes in the Δ*S*_solv_ and Δ*H*_s_ terms. The observed differences in these terms indirectly (Δ*S*_solv_) or directly (Δ*H*_s_) arise from the approximately 10% difference in the strength of hydrogen bonds in light versus heavy water^21−24^ and are usually balanced out, bringing the change in binding free energy close to zero (enthalpy–entropy compensation).^8,44,81,82^ Please note that in addition to Δ*S*_solv_ and Δ*H*_s_, the terms Δ*S*_conf_ and Δ*H*_*i*_ can also be affected, albeit more indirectly, through any effects of the light to heavy water exchange upon the conformational space of the solutes (particularly by interface water molecules). The decomposition outlined in eqs 7 and 8 largely neglects this nuanced interplay of the different terms but provides a simple framework to discuss the different contributions to the net thermodynamic properties.

Specific experimental results, as well as structure-based observations on the two model systems, are discussed in the following two subsections for trypsin and carbonic anhydrase II, respectively. In general, the results show no significant difference (<1 kcal/mol) between the Δ*G*_bind_ values in regular water and heavy water, as expected from the enthalpy–entropy compensation, which is usually present in systems with weak intermolecular interactions like hydrogen bonds. Ben-Naim and Marcus made discussions understanding enthalpy–entropy compensation in their work.^83^ The authors observed that the Gibbs free energy change associated with solvation is predominantly influenced by the enthalpy change, with the entropy playing a compensatory role. For nonpolar solutes like methane in water, the entropy change dominates in the solvation process; however, in cases where polar solutes or solutes in polar solvents (such as water in water) are considered, the enthalpy change tends to dominate, with the entropy change providing a compensating effect.^83^

### Binding of *p*-Substituted Benzamidines to Trypsin

**Figure 1:**
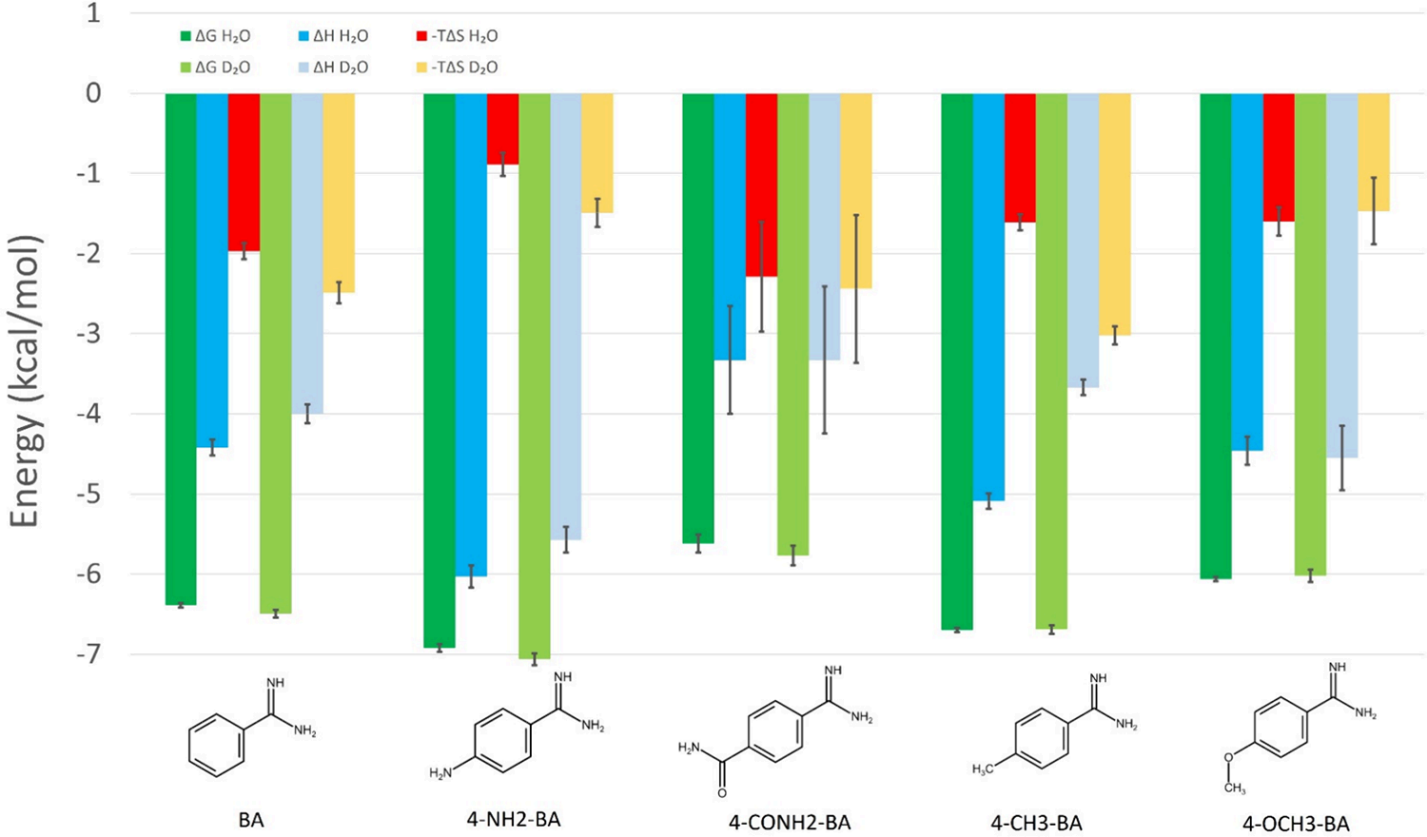
Thermodynamic signatures for the *p*-substituted benzamidines against trypsin in regular vs heavy water.

A series of five *p*-substituted benzamidines were evaluated against trypsin. Table 1 shows the thermodynamic data acquired from ITC measurements, including the changes in enthalpy between heavy and light water (ΔΔ*H*_bind_), while Figure 1 shows a direct comparison of the “thermodynamic signatures” of the ligands between heavy water and light water.

**Table 1:**
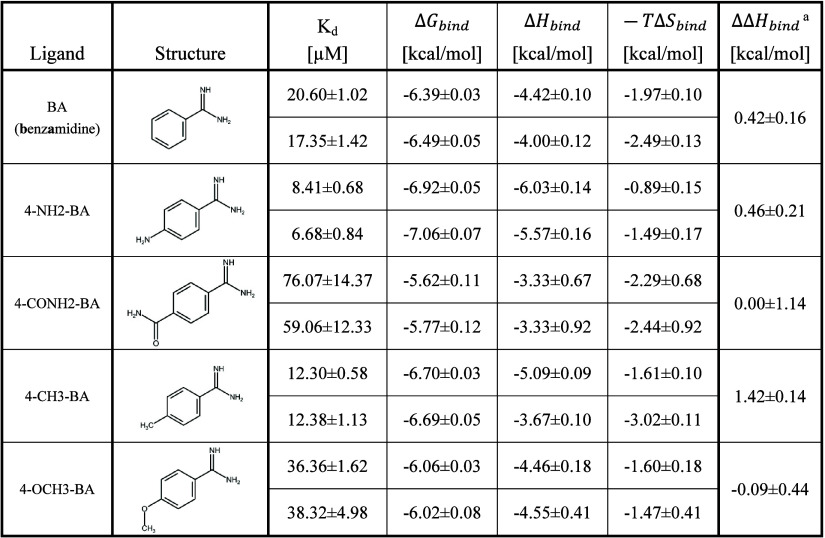
Binding Thermodynamics of *p*-Substituted Benzamidines to Trypsin in Light Water (Top) vs Heavy Water (Bottom)

a .

**Figure 2:**
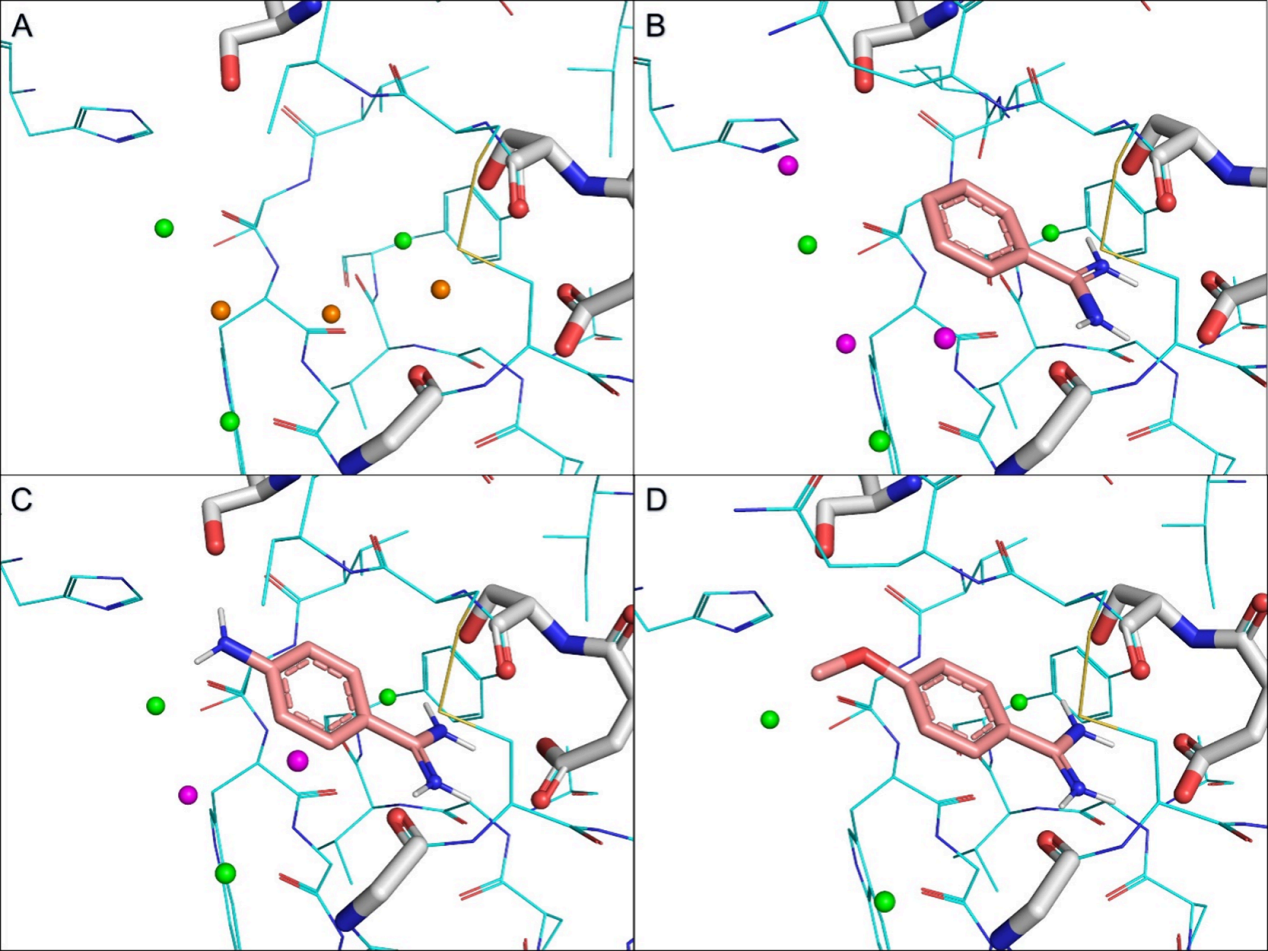
Water molecules in experimental structures of trypsin. Water molecules highlighted with orange spheres are removed from the binding site after ligand binding, while those highlighted with green spheres are retained across all structures. Water molecules highlighted with magenta spheres have no counterpart in the apo structure. (A) Joint X-ray/neutron diffraction structure of trypsin in its apo form (PDB ID 5MOP).^67^ (B–D) X-ray structures of trypsin complexed with (B) **BA** (PDB ID 1S0R),^58^ (C) **4-NH2-BA** (PDB ID 3GY4),^59^ and (D) **4-OCH3-BA** (PDB ID: 7WA2).^60^

To investigate the relationship between the ΔΔ*H*_bind_ values and the effect of ligand binding on the water network in the experimental structures, we first checked the water molecules for the apo protein (PDB ID 5MOP)^67^ and in the appropriate ligand–protein complex structure, where it was available (**BA**, **4-NH2-BA**, and **4-OCH3-BA**). Only structures with better than 2 Å resolution were considered to identify localized water molecules and minimize the uncertainty owing to unresolved ones. The structures with the waters in and near the binding site are shown in Figure 2. There are six water molecules near **BA**, five near **4-NH2-BA**, and three near **4-OCH3-BA**. It was found that there are conserved water molecules in the binding site that are retained in the complex structures, as well as in the apo structure. Comparing the complex structures against each other, we can find additional water molecules (that do not have a counterpart in the apo structure) near **4-NH2-BA** and **BA**, while we cannot find any additional water molecules near **4-OCH3-BA**. These results suggest that the water network in the proximity of **BA** and **4-NH2-BA** is similar, while the water network around **4-OCH3-BA** is slightly different (has less water molecules). This correlates with the ΔΔ*H*_bind_ results acquired from the ITC measurements, as **BA** and **4-NH2-BA** have very similar ΔΔ*H*_bind_ values (0.42 ± 0.16 kcal/mol for **BA** and 0.46 ± 0.21 kcal/mol for **4-NH2-BA**), while **4-OCH3-BA** has a slightly different ΔΔ*H*_bind_ value (−0.09 ± 0.44 kcal/mol).

In general, the overall affinity of the ligands does not significantly change in light versus heavy water, highlighting the role of enthalpy–entropy compensation, especially in the case of **4-CH3-BA**, where a significant change in light versus heavy water binding enthalpy could be detected. Regarding the trend within this series, we can notice that the larger amide and methoxy groups confer weaker *K*_d_ and smaller ΔΔ*H*_bind_ values than unsubstituted **BA**, while the smaller amine and methyl groups slightly improve affinity and increase ΔΔ*H*_bind_. The observation that larger ligands show smaller ΔΔ*H*_bind_ suggests that ΔΔ*H*_bind_ depends not only on the water network perturbation but also other factors, like what the differential solute–solute versus solute–solvent H-bonds may contribute. This latter may reduce the magnitude of ΔΔ*H*_bind_, the binding enthalpy difference in H_2_O versus D_2_O, and weaken the correlation between water network reorganization and ΔΔ*H*_bind_ and may eventually lead to negative ΔΔ*H*_bind_.^21,30,32,35^

### Water Network Analysis in Trypsin by WaterFLAP

The water network perturbation upon ligand binding was also analyzed by the modeling methods WaterFLAP^39,48^ and MobyWat.^53^ To validate the water molecules predicted by WaterFLAP, good-quality X-ray diffraction (resolution ≤1 Å) and neutron diffraction structures (resolution ≤1.8 Å) were used (5MNZ and 5MNF).^67^ Water molecules predicted by WaterFLAP were compared with the structural waters inside the binding site. The X-ray diffraction structure had seven water molecules in the binding site, each having a predicted water in its 1.5 Å proximity, while the neutron diffraction structure had five water molecules in the binding site, each also having a predicted water in its 1.5 Å proximity (Figure S1). Moreover, WaterFLAP predicted a denser water network, containing more water molecules compared to the X-ray and neutron diffraction structures.

**Figure 3:**
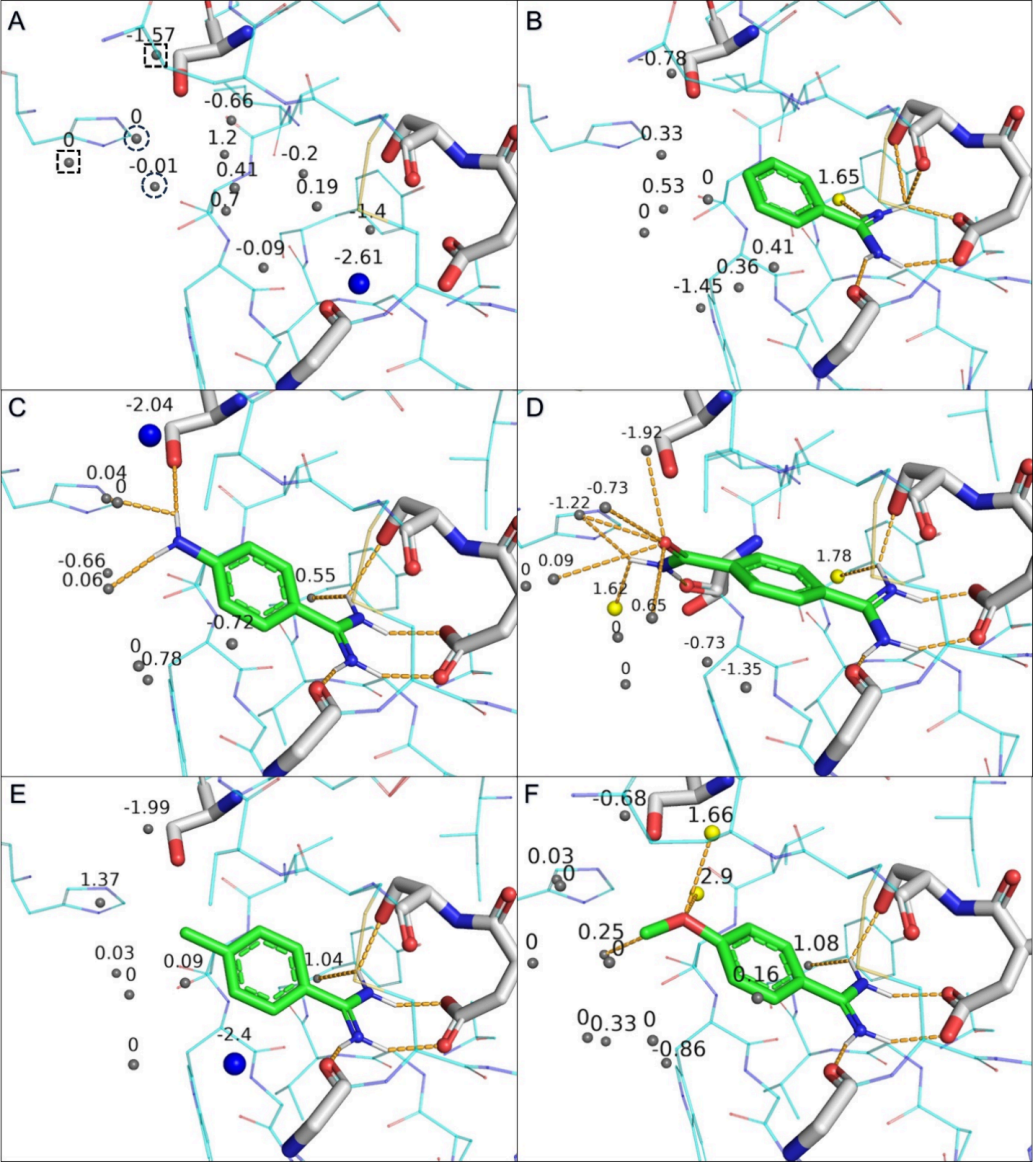
Water molecules predicted by WaterFLAP inside the binding site of trypsin, with all waters labeled with their calculated Δ*G* values from WaterFLAP. H-bonds are shown as orange dashed lines. (A) Empty binding site of trypsin filled with water molecules. There is one “happy” (blue) and 12 bulk-like (gray) waters inside the binding site. Squared waters are displaced upon the binding of **4-CONH2-BA** and **4-OCH3-BA**, the circled water molecules are displaced by all ligands except **BA**, and the rest of the water molecules, including the “happy” water, are displaced by all ligands. (B) **BA** has one “unhappy” (yellow) and eight bulk-like water molecules in its proximity and establishes an H-bond with the “unhappy” water. (C) **4-NH2-BA** has one “happy” and eight bulk-like water molecules in its proximity and establishes H-bonds with the S195 residue and three bulk-like waters. (D) **4-CONH2-BA** has two “unhappy” and 10 bulk-like water molecules in its proximity and establishes H-bonds with both “unhappy” waters and five bulk-like waters. (E) **4-CH3-BA** has one “happy” and seven bulk-like water molecules in its proximity and establishes an H-bond with a bulk-like water. (F) **4-OCH3-BA** has two “unhappy” and 12 bulk-like water molecules in its proximity and establishes H-bonds with both “unhappy” waters and two bulk-like waters.

We examined which waters placed by WaterFLAP in the empty binding site of trypsin are replaced by the various ligands. The squared waters in Figure 3A are displaced only by **4-CONH2-BA** and **4-OCH3-BA**, the circled waters are displaced by all ligands except **BA**, and the rest of the displayed waters are displaced by all trypsin ligands. We can observe a “happy” (Δ*G* < −2 kcal/mol) water with a calculated Δ*G* of −2.61 kcal/mol (Figure 3A) that is displaced by all ligands, which works against their overall affinity and leads to a modest variation in ΔΔ*H*_bind_. A positionally matching structural water molecule with this “happy” water molecule can also be found within the good-quality X-ray and neutron diffraction structures (Figure S1). Examining the waters close to the *para*-position of **BA** (Figure 3B), or the circled waters in Figure 3A, we can only find bulk-like waters (−2 kcal/mol ≤ Δ*G* < 1.5 kcal/mol) with low Δ*G* values (+0.33, −0.01, or 0 kcal/mol), meaning that their displacement does not affect the affinity significantly in the case of **4-CH3-BA** and **4-NH2-BA**. One of the squared bulk-like waters in Figure 3A has a calculated Δ*G* of −1.57 kcal/mol, being in a thermodynamically favorable state (although not termed a “happy” water by definition), and its displacement could explain the decrease in binding affinity for **4-CONH2-BA** and **4-OCH3-BA**. In the case of **4-NH2-BA**, the amine group establishes H-bonds with bulk-like waters, but the substituents of **4-OCH3-BA** and **4-CONH2-BA** also make H-bonds with “unhappy” (3 kcal/mol > Δ*G* ≥ 1.5 kcal/mol) waters. The presence of these unhappy water molecules contributes to the affinity decrease of these compounds.

It is worth also noting that **4-CH3-BA** shows a higher ΔΔ*H*_bind_ value of +1.42 ± 0.14 kcal/mol than the other compounds (ΔΔ*H*_bind_ < 0.5 kcal/mol). As the difference between the two types of compounds is in the number of ligand–protein and ligand–water H-bonds, we assume that these H-bonds and their variation in the bound and unbound states contribute to the observed ΔΔ*H*_bind_.

An assessment of the free energy change of water upon ligand binding was obtained by summing Δ*G*_bind_ values of waters (as evaluated from the apo structure) that are displaced during complex formation and ΔΔ*G*_bind_ of waters that are conserved in the complex, the latter being obtained by WaterFLAP water perturbation analysis (Table S1). These data show that calculated water network free energy changes associated with ligand binding (Δ*G*_waters_) do not correlate with Δ*G*_bind_ nor with ΔΔ*H*_bind_ values. We note that a lack of correlation between ΔΔ*H*_bind_ and Δ*H*_bind_ or Δ*G*_bind_ of ligands was previously observed in ref (21).

### Water Network Analysis in Trypsin by MobyWat

The positions of surface and interface water molecules were calculated for trypsin and **BA**, **4-NH2-BA**, and **4-OCH3-BA** complexes by MobyWat as described in the methods section. The success rate (SR) values are detailed in Table S6 (the match tolerance was set to 1.5 Å in agreement with what was used for WaterFLAP).

Due to the natural uncertainty of surface water molecules, their prediction tends to result in lower success rates around 80%.^84^ However, in the present case of the trypsin target, an 87.5% SR was achieved. The prediction of interface water molecules is more accurate; often, an SR of 90% is reachable.^84,85^ MobyWat indeed achieved an SR of 100% for two of the three interfaces, and in the remaining case, an SR of 83% was found (Table S6).^84,85^

**Figure 4:**
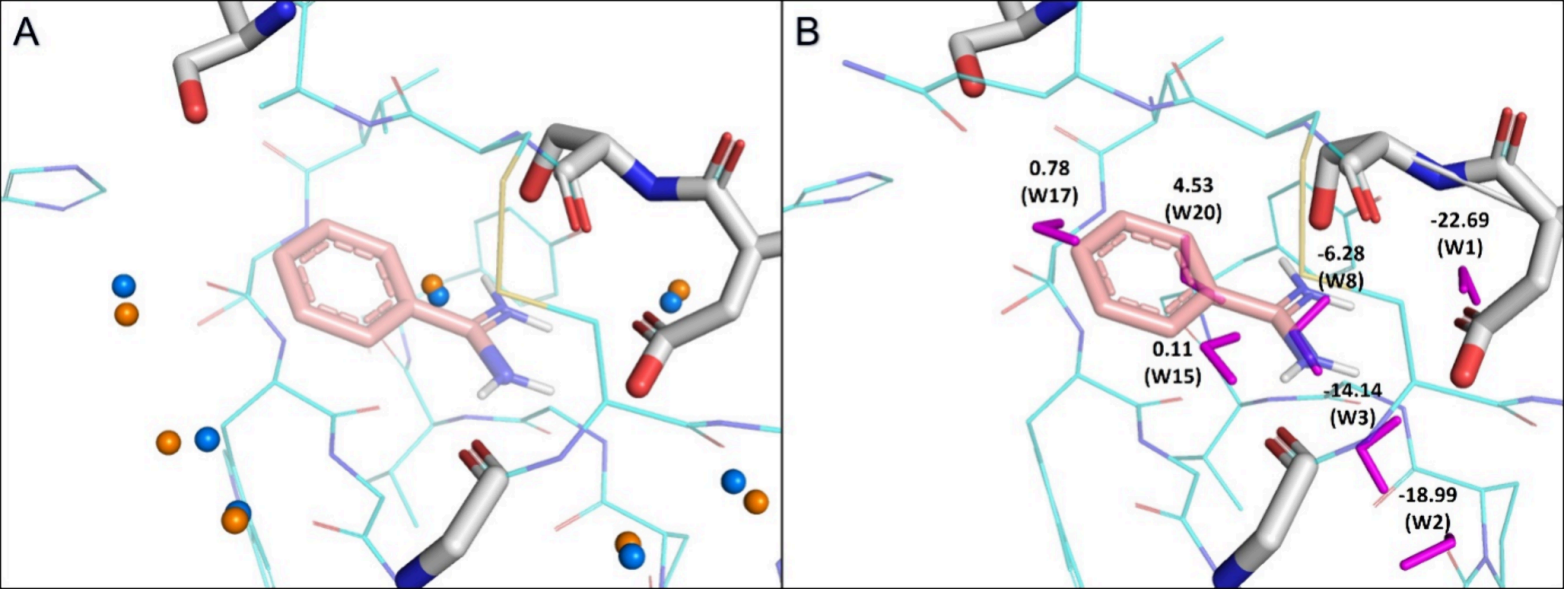
(A) Comparison of predicted (blue spheres) and experimental water positions (orange spheres) (PDB ID 5MO0).^67^ (B) Calculation of individual water molecule binding enthalpies. The binding enthalpies of water molecules (kcal/mol) and their corresponding serial numbers are labeled (Table S4). **BA** is shown as brownish-red sticks. Water molecules are colored purple. Overlapping water molecules that are displaced by benzamidine are labeled W8, W15, W17, and W20. These water molecules are likely to leave the binding site. Staying, bridging water molecules are labeled W1, W2, and W3.

Benzamidine derivatives were placed into the apo target with MobyWat-predicted surface water molecules to check for overlapping positions (**BA**, Figure 4B). The overlapping water molecules are displaced by the binding of the derivatives. **BA** and **4-NH2-BA** displace the same water molecules, and **4-OCH3-BA** displaces an additional water, which is in good agreement with the previous findings using WaterFLAP. **4-CONH2-BA** displaces W4 (Table S4), an enthalpically favorable water, which might contribute to the weaker binding affinity of the compound compared to the unsubstituted **BA**, which does not displace W4. **4-CONH2-BA** displaces the most water molecules (Table S4), which explains its highest entropic contribution to binding, as observed in ITC measurements (Figure 1).

### Binding of Aromatic Sulfonamides to Carbonic Anhydrase II

**Figure 5:**
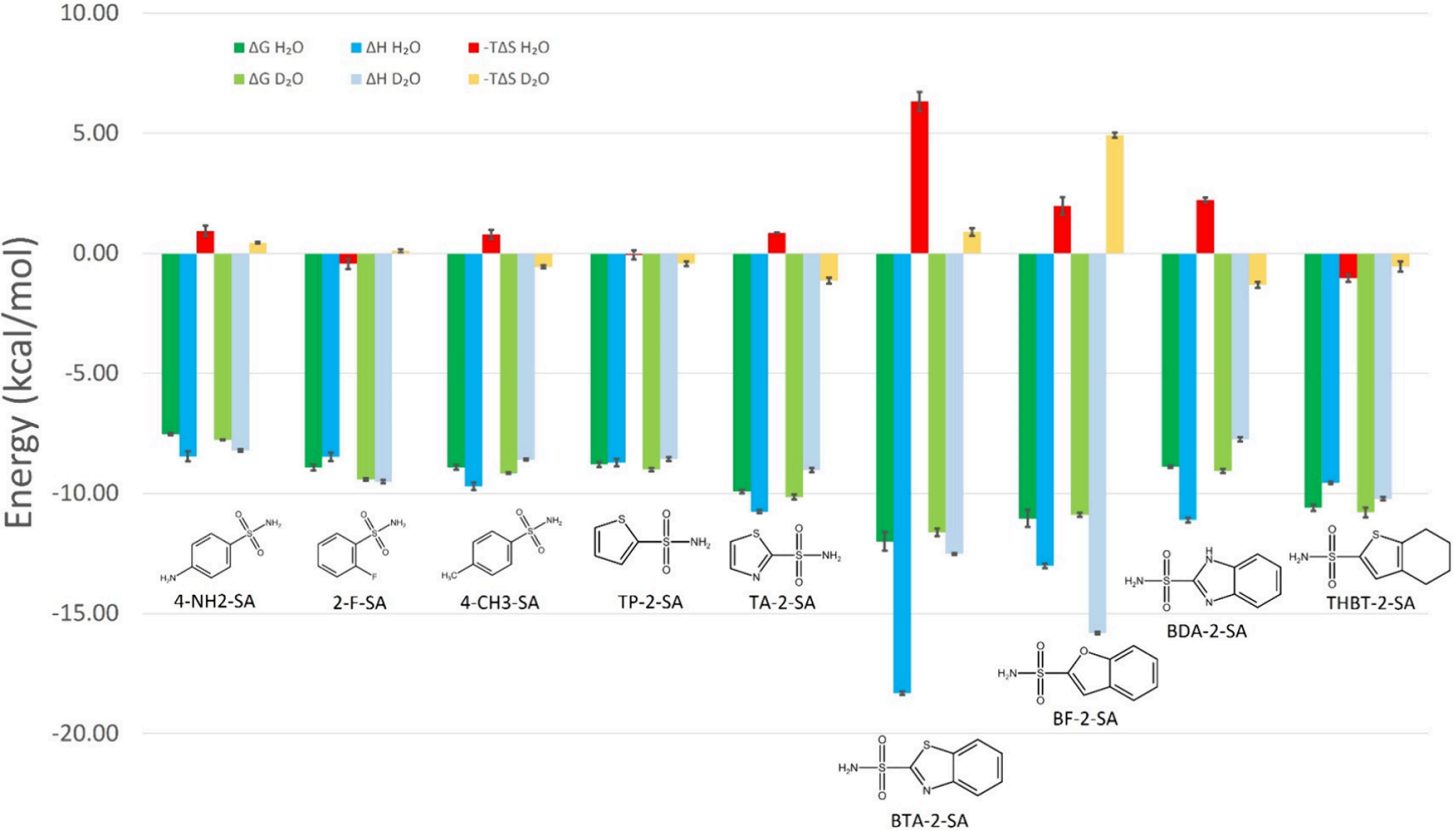
Thermodynamic signatures for the aromatic sulfonamides against carbonic anhydrase II in regular vs heavy water.

In this case, a series of nine aromatic and heteroaromatic sulfonamides were evaluated against carbonic anhydrase II. Table 2 shows the thermodynamic data acquired from ITC measurements, including the changes in enthalpy between heavy and light water (ΔΔ*H*_bind_), while Figure 5 shows a direct comparison of the “thermodynamic signatures” of the ligands between heavy water and light water.

**Table 2:**
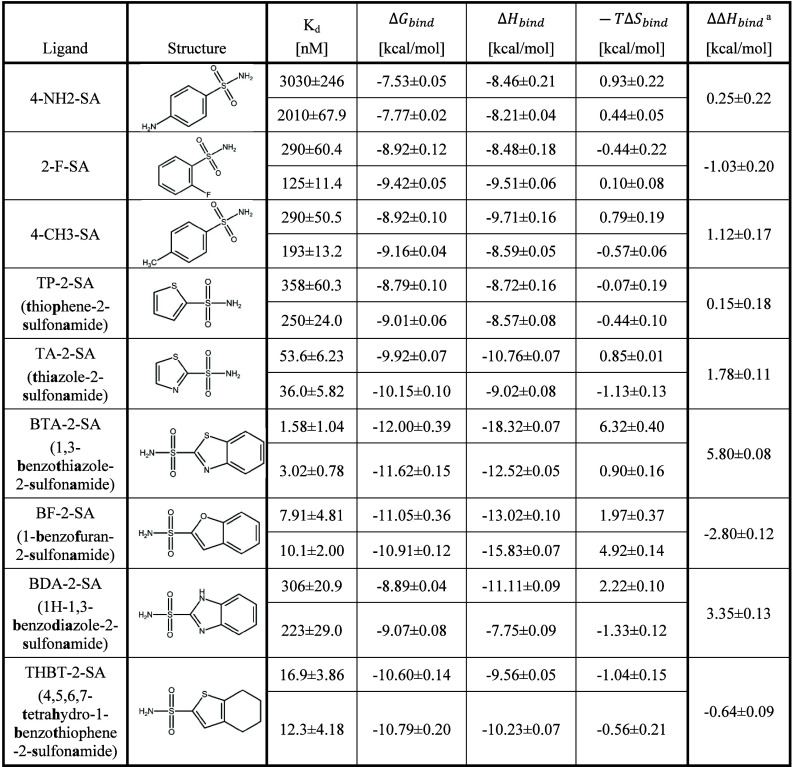
Binding Thermodynamics of Aromatic and Heteroaromatic Sulfonamides against Carbonic Anhydrase II in Light Water (Top) vs Heavy Water (Bottom)

a .

We obtained more exothermic binding in D_2_O than in H_2_O for three ligands (**2-F-SA**, **BF-2-SA**, and **THBT-2-SA**). This phenomenon is unusual, although it has been observed and interpreted as the consequence of different H-bond patterns for the solvated and bound species.^32,35^ More specifically, we suggest that water molecules participating in the solvation of the solutes before ligand binding have a more favorable binding enthalpy in D_2_O compared to H_2_O, and the difference compensates for the stronger D_2_O versus H_2_O binding when these water molecules are released to the bulk. In other words, the relative binding enthalpy of H_2_O and D_2_O molecules within the protein binding site is expected to be context-dependent. We note that the assignment of varying Δ*G*_bind_ values to bound water molecules is a useful model; there are several documented examples where water molecules with low and high Δ*G*_bind_ values were identified, and the displacement of the latter upon ligand binding was shown to favorably contribute to ligand affinity (binding free energy).^66,86,87^ Similarly, water molecules in the protein binding site may have varying Δ*H*_bind_ values, and their displacement by ligands may give different contributions in D_2_O and H_2_O.

**Figure 6:**
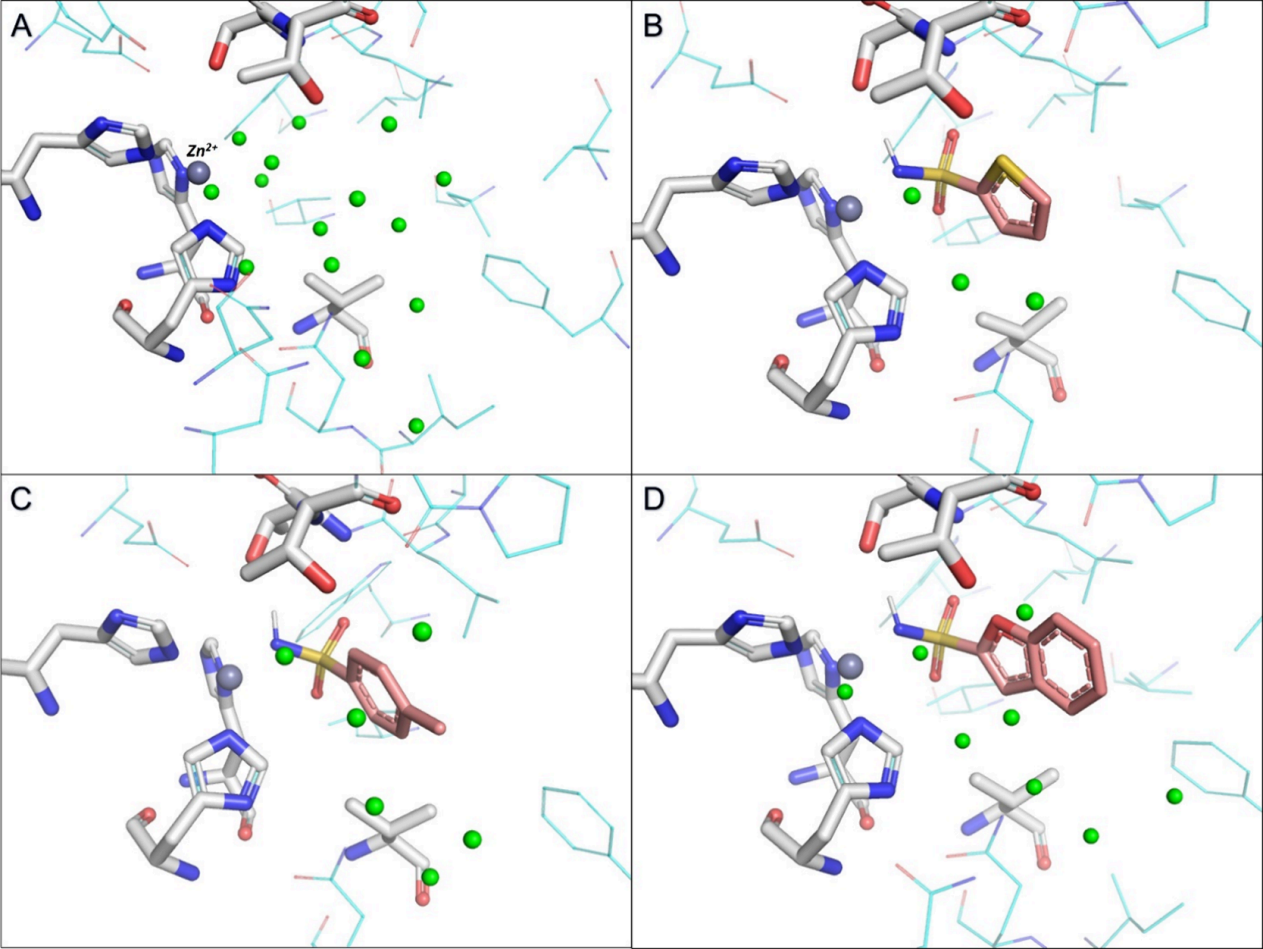
Water molecules (highlighted with green) in experimental structures of CAII. (A) X-ray structure of CAII in its apo form (PDB ID 3KS3).^88^ (B–D) X-ray structures of CAII complexed with (B) **TA-2-SA** (PDB ID 3S77),^45^ (C) **4-CH3-SA** (PDB ID 4YXI),^62^ and (D) **BF-2-SA** (PDB ID 3S71).^45^

Just as for trypsin, we first checked the water molecules in the experimental structures for the apo protein (PDB ID 3KS3)^88^ and for the ligand–protein complex structures, where they were available (all ligands except **THBT-2-SA**), and analyzed the corresponding ΔΔ*H*_bind_ values. The structures with the waters in and near the binding site for **TP-2-SA**, **4-CH3-SA**, and **BF-2-SA** are shown in Figure 6. For the rest of the ligands, please refer to Figure S3. Analysis of the experimental structures is based on the number of water molecules being in the proximity of the ligand. Within the binding site, and in its proximity, there are three water molecules near **TP-2-SA**, four water molecules near **4-NH2-SA**, six water molecules near **2-F-SA**, **4-CH3-SA**, and **BTA-2-SA**, seven water molecules near **TA-2-SA** and **BDA-2-SA**, and eight water molecules near **BF-2-SA**. The larger number of ordered water molecules in the complexes of ligands with fused rings compared to those with a single ring is reflected in the unfavorable binding entropy (negative *T*Δ*S*) of the former compounds. Likewise, we can detect a correlation between the ΔΔ*H*_bind_ values and the number of water molecules near the ligands: ligands with minimal ΔΔ*H*_bind_ values (**4-NH2-SA** and **TP-2-SA**) have the smallest amount of water molecules nearby (four for **4-NH2-SA** and three for **TP-2-SA**), ligands with ΔΔ*H*_bind_ values between +1 and +2 kcal/mol or −1 and −2 kcal/mol (**2-F-SA**, **4-CH3-SA**, and **TA-2-SA**) have six or seven water molecules nearby, while ligands with ΔΔ*H*_bind_ values less than −2 kcal/mol or more than +2 kcal/mol (**BTA-2-SA**, **BF-2-SA**, and **BDA-2-SA**) have either six, seven, or eight water molecules nearby. This result suggests a connection between the network of localized water molecules around the ligand and the magnitude of ΔΔ*H*_bind_ values acquired from the ITC measurements. This also correlates with the results acquired for trypsin (see above), where the ligands with larger ΔΔ*H*_bind_ values (**BA** and **4-NH2-BA**) have more water molecules in their proximity than **4-OCH3-BA** with a lower ΔΔ*H*_bind_ value.

The CAII ligands comprised two related series: five ligands containing a single aromatic ring (five- or six-membered) vs four ligands containing fused heteroaromatic rings. Inspecting the ITC results, we observe significantly larger differences in ΔΔ*H*_bind_ for the latter group. This is accompanied by an unfavorable entropy change upon binding (*T*Δ*S*_bind_ < 0 for **BTA-2-SA**, **BF-2-SA**, and **BDA-2-SA**) that suggests the presence of trapped water molecules in the binding site. This is in line with the increased number of water molecules around these ligands as observed in the X-ray structures (see above).

The ligands **4-NH2-SA**, **2-F-SA**, and **4-CH3-SA** are similar in structure, as all of them are benzenesulfonamides with an additional functional group. Compound **2-F-SA** has a negative ΔΔ*H*_bind_ value of −1.03 ± 0.20 kcal/mol that is opposite in sign to what is expected from the water network rearrangement, and an important contribution from solute–solute and solute–solvent H-bonds can be assumed.^21,32,35^ It is also worth noting that **2-F-SA** is a smaller, more “compact” molecule than the other two benzenesulfonamides, and it may have an effect on its water network perturbation and unique ΔΔ*H*_bind_. The affinity of **2-F-SA** could also be enhanced by a nontypical polar contact between the fluorine atom and the backbone of CAII, impacting its ability to displace waters in the binding site.^43^ Considering the other two benzenesulfonamides, it is interesting to see that **4-CH3-SA** with an apolar substituent has a higher ΔΔ*H*_bind_ compared to **4-NH2-SA** with a polar substituent, similarly to what was observed for substituted benzamidine-trypsin complexes (see **4-CH3-BA** versus **4-NH2-BA** and **4-OCH3-BA** versus **4-CONH2-BA** in Table 1).

A general observation for the ligands containing a heteroaromatic ring (**TP-2-SA**, **TA-2-SA**, **BTA-2-SA**, **BF-2-SA**, **BDA-2-SA**, and **THBT-2-SA**) is that compounds with two heteroatoms in their aromatic ring tend to have more positive ΔΔ*H*_bind_ values than those with only one heteroatom. **TA-2-SA** and **TP-2-SA** are both small ligands with a single five-membered aromatic ring and have positive ΔΔ*H*_bind_ values of different magnitudes (+1.78 ± 0.11 and +0.15 ± 0.18 kcal/mol for **TA-2-SA** and **TP-2-SA**, respectively), consistent with the number of heteroatoms (two in **TA-2-SA** vs one in **TP-2-SA**). The nitrogen in the thiazole ring can make H-bonds, while the sulfur atom in the thiophene ring is a softer nucleophile and a weaker H-bond acceptor in general.^89,90^ Examining their binding modes, **TA-2-SA** can establish a polar interaction with the T199 residue, which could explain its lower *K*_d_ compared to **TP-2-SA**. The interaction with this residue resulting in an increase in binding affinity has been observed previously in ref (91).

**BF-2-SA** is structurally similar to **THBT-2-SA**, as it also contains one heteroatom in its ring (oxygen instead of sulfur), with the additional difference that it has a fused benzene ring instead of a fused cyclohexane ring. The same phenomenon that compounds with a single heteroatom have lower ΔΔ*H*_bind_ values can be observed here (in fact, **BF-2-SA** has the most negative ΔΔ*H*_bind_ value out of all examined ligands, with −2.80 ± 0.12 kcal/mol). **BF-2-SA** can also establish a polar interaction with the T199 residue, resulting in its lower *K*_d_ value compared to **THBT-2-SA**.

On the other hand, **BDA-2-SA** is structurally similar to **BTA-2-SA**, with the sulfur atom being replaced with a nitrogen atom. It has a ΔΔ*H*_bind_ value of +3.35 ± 0.13 kcal/mol, which correlates with the above-mentioned observation regarding the number of heteroatoms. It is interesting to note that the affinity of **BDA-2-SA** is significantly worse than that of **BTA-2-SA**, even though both establish polar interactions with the T199 residue. A possible reason is that **BDA-2-SA** contains an NH-type H-bond donor pointing toward the apolar V121 residue, resulting in an unfavorable interaction with the protein.

### Water Network Analysis in Carbonic Anhydrase II by WaterFLAP

The quality of WaterFLAP prediction was investigated by comparing predicted water positions with those of good-quality X-ray diffraction (resolution ≤1 Å) and neutron diffraction structures (resolution ≤1.8 Å) (4Q49^92^ and 3KS3^88^). The X-ray diffraction structure has 13 water molecules in the binding site, nine of which have a predicted water in its 1.5 Å proximity, while the neutron diffraction structure has eight water molecules in the binding site, seven of which have a predicted water in its 1.5 Å proximity (Figure S2). Similarly to what was observed for trypsin, WaterFLAP predicted a denser water network, containing more water molecules compared to the X-ray and neutron diffraction structures also for CAII.

**Figure 7:**
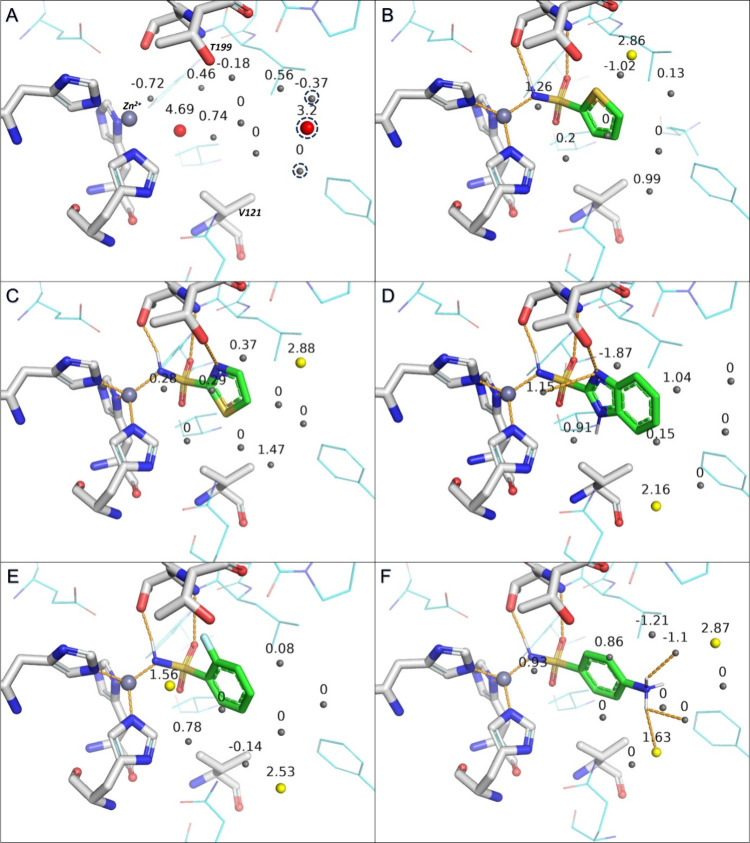
Water molecules predicted by WaterFLAP inside the binding site of CAII, labeled with their calculated Δ*G* values. Interactions are shown as orange dashed lines. (A) Empty binding site of CAII filled with water molecules. There are two “very unhappy” (red, Δ*G* ≥ 3 kcal/mol) and nine bulk-like (gray) waters inside the binding site. These water molecules are displaced upon ligand binding, with the circled water molecules only displaced by the four ligands containing fused heteroaromatic rings (**BTA-2-SA**, **BF-2-SA**, **BDA-2-SA**, and **THBT-2-SA**). The T199 and V121 residues are labeled. (B) Binding mode of **TP-2-SA** in CAII, with one “unhappy” and seven bulk-like water molecules in its proximity. (C) Binding mode of **TA-2-SA** in CAII, with one “unhappy” and eight bulk-like water molecules in its proximity and an H-bond with the T199 side chain. (D) Binding mode of **BDA-2-SA** in CAII, with one “unhappy” and eight bulk-like water molecules in its proximity and an H-bond with a bulk-like water molecule. (E) Binding mode of **2-F-SA** in CAII, with two “unhappy” and six bulk-like water molecules in its proximity. (F) Binding mode of **4-NH2-SA** in CAII, with one “unhappy” and nine bulk-like water molecules in its proximity and three H-bonds with two bulk-like waters and with the “unhappy” water molecule.

Based on the WaterFLAP results, the binding site contains nine bulk-like and two “very unhappy” (Δ*G* ≥ 3 kcal/mol) waters (Figure 7A), meaning that ligand binding should be very favorable in general. Positionally matching structural water molecules with these two “very unhappy” water molecules can also be found within the good-quality X-ray diffraction structure (Figure S2A). All ligands here displace all except three of the aforementioned waters (one “very unhappy” and two bulk-like waters, circled in Figure 7A), which are only displaced by the ligands containing fused heteroaromatic rings (**BTA-2-SA**, **BF-2-SA**, **BDA-2-SA**, and **THBT-2-SA**). The highly beneficial contribution of water displacement to the binding free energy is in line with the observed high affinities (*K*_d_ < 3.1 μM). Examining their experimental or predicted binding modes (for **THBT-2-SA**), ligands with a single heteroatom (**TP-2-SA**, **BF-2-SA**, and **THBT-2-SA**) expose their heteroatom toward the T199 residue, and the hydrophobic part of the ring points toward the V121 residue. These interactions contribute to the stabilization of binding poses, and the hydrophobic part of the ring pointing toward the V121 is better suited for displacing the “very unhappy” waters of the binding site and establishing a local hydrophobic contact with V121.

**TP-2-SA** and **TA-2-SA** contain five-membered heteroaromatic rings without substituents, and both of them displace the “very unhappy” water molecule with a Δ*G* value of +4.69 kcal/mol from the binding site, increasing their affinity. Nonetheless, both of them host one “unhappy” water molecule in their respective binding poses, with Δ*G* values of +2.86 kcal/mol for **TP-2-SA** (Figure 7B) and +2.88 kcal/mol for **TA-2-SA** (Figure 7C). The almost identical Δ*G* values suggest that the water networks near these ligands are approximately the same, which in turn means that the difference between their *K*_d_ values (358 nM for **TA-2-SA** and 53.6 nM for **TP-2-SA**) cannot be explained with the water network. Instead, we propose that the ability of **TA-2-SA** to establish a polar interaction with the T199 residue could be a discriminating factor here.

In comparison to **TP-2-SA**, **THBT-2-SA** has an additional, fused cyclohexane ring, which can displace the “unhappy” water in the structure of **TP-2-SA** (in addition to both “very unhappy” waters detected in the empty binding site), explaining the significant increase in affinity (*K*_d_ = 16.9 nM vs 358 nM) (Figure S4B).

Similarly, **BTA-2-SA** adds a fused ring to the core structure of **TA-2-SA**, displacing the “unhappy” water in the structure of **TA-2-SA** (in addition to both “very unhappy” waters detected in the empty binding site), again resulting in a significant increase in affinity compared to **TA-2-SA** (*K*_d_ = 1.58 nM vs 53.6 nM). **BTA-2-SA** also has a lower *K*_d_ than **THBT-2-SA**, which could be explained with its ability to interact with the T199 residue, similarly to the case of **TA-2-SA** compared to **TP-2-SA**. **BTA-2-SA** shows the highest binding enthalpy gain in H_2_O versus D_2_O (ΔΔ*H*_bind_ = 5.8 ± 0.08 kcal/mol). We suggest that it is the consequence of having a large number of trapped water molecules in the complex; moreover, two out of these waters are associated with high binding free energies: one with a Δ*G* value of +6.15 kcal/mol (most positive value out of all the examined waters in CAII) and one with +2.23 kcal/mol (Figure S4D). These observations indicate special ligand–water interactions that are reflected in the increased effect of the solvent isotopic substitution.

Unlike **BTA-2-SA** and **THBT-2-SA**, there are no measured single-ring analogs for **BDA-2-SA** and **BF-2-SA**. As they are also ligands with fused heteroaromatic rings, they also displace both “very unhappy” waters in the binding site, accounting for the general increase in their binding affinity, which correlates with **BF-2-SA** having the second strongest *K*_d_ (7.91 nM). For the predicted water molecules near **BF-2-SA**, please refer to Figure S4C. To find an explanation for the unexpectedly weak *K*_d_ of **BDA-2-SA**, we can compare the waters hosted by the four ligands (**BTA-2-SA**, **BDA-2-SA**, **BF-2-SA**, and **THBT-2-SA**). All of them host “unhappy” water molecules, but **BDA-2-SA** can also establish an H-bond with one of the waters labeled as bulk-like but having a relatively high calculated Δ*G* of +1.15 kcal/mol (Figure 7D). Similarly to the cases of **4-CONH2-BA** and **4-OCH3-BA** for trypsin, this could result in a further decrease in binding affinity due to limiting the ability of the water molecule to escape from a thermodynamically unfavorable state (positive Δ*G*). This, combined with the previously discussed structural feature of **BDA-2-SA** (NH-type H-bond donor pointing toward the apolar V121 residue), could explain the observed increase in *K*_d_ compared to the other ligands with fused heteroaromatic rings.

**2-F-SA**, **4-NH2-SA**, and **4-CH3-SA** are ligands containing substituted benzene rings. **2-F-SA** is the most “compact” molecule among all nine ligands. Analyzing the waters hosted by **2-F-SA**, we can find two “unhappy” waters with calculated Δ*G* values of +1.56 and +2.53 kcal/mol and six bulk-like waters (Figure 7E). In the *para*-position of **2-F-SA**, we can only find low-energy bulk-like waters, meaning that substituents at that position should not affect the *K*_d_ value significantly. This correlates with **4-CH3-SA** having the same mean *K*_d_ as **2-F-SA** (*K*_d_ = 290 nM); however, it is not the case for **4-NH2-SA** (*K*_d_ = 3030 nM). Analyzing the binding mode of **4-CH3-SA** (Figure S4A) and **4-NH2-SA** (Figure 7F), we observe the (protonated) amine group in **4-NH2-SA** establishing H-bonds with one “unhappy” and two bulk-like waters. As in the cases of **4-OCH3-BA**, **4-CONH2-BA**, and **BDA-2-SA**, H-bonding to thermodynamically unfavorable waters could be the reason for an unexpected increase in *K*_d_. The lower affinity of **4-NH2-SA** could also result from the more drastic desolvation penalties associated with the protonated state of the amino substituent.^43^

Similarly to the calculation performed for trypsin, an assessment of the free energy change of water upon ligand binding was obtained. Data are presented in Table S2. No correlation between ligand binding-associated water free energy change (Δ*G*_waters_) and either Δ*G*_bind_ or ΔΔ*H*_bind_ values was found similarly to the case of trypsin (see above).

### Water Network Analysis in Carbonic Anhydrase II by MobyWat

The void-free binding site of the apo carbonic anhydrase includes 14 surface water molecules based on MobyWat prediction, which is approximately twice as much as the waters assigned in the X-ray and neutron diffraction experimental structures (4Q49 and 3KS3). MobyWat predicted the interface water positions with a more than 80% success rate (SR) in most of the cases (Table S6), using the experimentally assigned waters as references.

**Figure 8:**
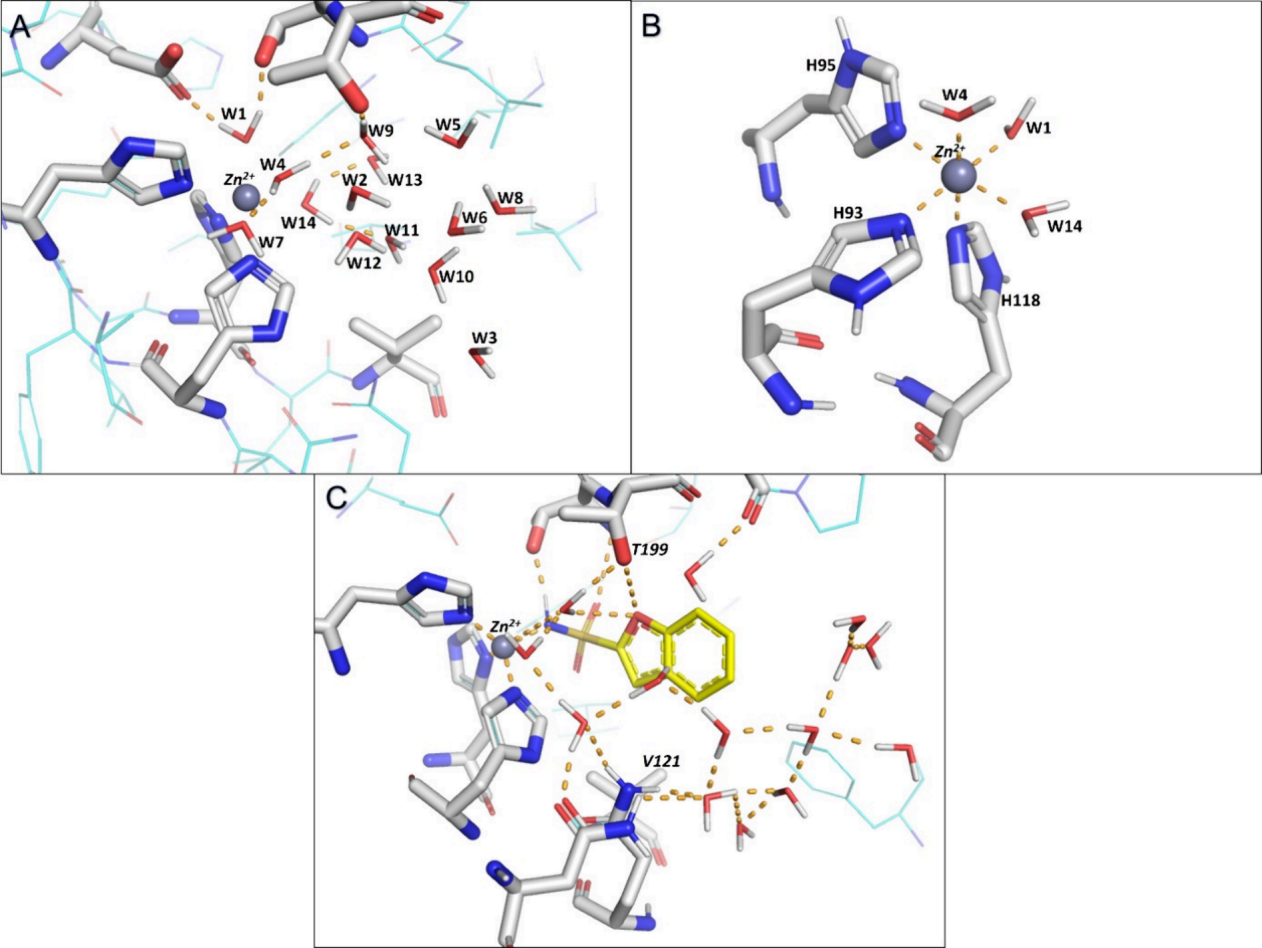
Water molecules predicted by MobyWat. Interactions are shown as dashed lines. (A) Empty binding site of CAII filled with water molecules. Δ*H*_bind_ values of each water molecule are included in Table S5. (B) Central six-coordinated Zn^2+^ ion inside apo CAII. (C) Water network predicted by MobyWat in the proximity of **BF-2-SA**.

The Δ*H*_bind_ of the individual water molecules to the apo target was also calculated as it was described in methods. In the ligand-free structure, MobyWat predicted 3 waters (W1, W4, and W14) around the Zn^2+^ ion that together with the three target histidines (H93, H95, and H118) resulted in a six-coordinated central Zn^2+^ ion (Figure 8B).

W14 with a positive Δ*H*_bind_ of +7.15 kcal/mol is located in a hydrophobic pocket (Figure 8A) including several apolar target residues such as V120, L139, V141, L196, and V205; thus, the displacement of W14 will contribute favorably to the binding enthalpy of all of the studied sulfonamides. In contrast, W1 with a Δ*H*_bind_ of −26.01 kcal/mol has a bridging role between the central Zn^2+^ ion and the E105 and T198 target residues; therefore, its displacement would result in a positive (unfavorable) contribution to the ligand binding enthalpy (Figure 8A).

All ligands displaced the two enthalpically most unfavorable waters (W13 and W14), out of which one (W14) was coordinated by the Zn^2+^ ion (Figure 8A). Ligands can be divided into two groups according to which Zn^2+^-coordinated water remained (W1 or W4) after ligand binding. **4-NH2-SA**, **2-F-SA**, **4-CH3-SA**, and **TP-2-SA** displaced W4 while **TA-2-SA**, **BTA-2-SA**, **BDA-2-SA**, and **THBT-2-SA** displaced W1 upon ligand binding. Only **BF-2-SA** displaced all three waters from the Zn^2+^-coordination and the highest number of waters in general (Table S5), leading to a highly stable tetrahedral coordination sphere with an extra water molecule coordinating the metal ion, as well as the most extensive water network rearrangement (Figure 8C). These result in a major impact on the binding thermodynamics of **BF-2-SA**, reflected in the lowest ΔΔ*H*_bind_ value (Table 2) and high −*T*Δ*S* values in both heavy and light water (Figure 5). These findings are in good agreement with previous work.^93^ Furthermore, a quantum chemical study showed that even such a fifth water ligand can considerably contribute to the stability of the aqua complexes of Zn^2+^.^94^

**TA-2-SA** differs from **TP-2-SA** only with a N atom in the heterocycle that leads to an order of magnitude stronger affinity for **TA-2-SA** compared to **TP-2-SA** (Table 2). The structural explanation of this result could be the stabilizing interaction between the thiazole group of **TA-2-SA** and W4 coordinated by Zn^2+^, while **TP-2-SA** is fixed loosely into the apolar subpocket through its thiophene heterocycle, without any water molecules or target residues contributing to ligand stabilization. This is supported by the fact that the ΔΔ*H*_bind_, as well as the affinity of **TP-2-SA**, is much lower than that of **TA-2-SA**, reflecting the minimal effect of waters on ligand binding for the former (and a considerable effect for the latter, see Table 2).

**BTA-2-SA** contains a benzyl ring coupled to **TA-2-SA**; however, the binding thermodynamic properties (*K*_d_ and ΔΔ*H*_bind_) vary significantly. Both ligands create two H-bonds with T198 through the sulfonamide moiety as described in a previous study.^45^ The N atom of the condensed ring of **BTA-2-SA** is attached directly to T199 that is thermodynamically more stable than the W4 water-coupled Zn^2+^ interaction of **TA-2-SA**, which may have a crucial role in ligand binding/stabilization, as indicated by the high ΔΔ*H*_bind_ value.

**BDA-2-SA** differs from **BTA-2-SA** only in the second heteroatom (N instead of S) in the condensed heterocycle. **BDA-2-SA** is stabilized mainly by apolar interactions, and the benzimidazole ring is connected to none of the target residues, leading to a relatively loose binding. One of the N atoms of **BDA-2-SA** (that is located in the same place as in **BTA-2-SA** and **TA-2-SA**) is not linked to the W4 water; furthermore, the other N atom connects to one water molecule entrapped between the apolar subpocket and the ligand, which could further weaken the apolar interactions. All these structural aspects could explain the lowest affinity for **BDA-2-SA** of these three ligands (Table 2).

Despite of the structural similarity of **TA-2-SA**, **BTA-2-SA**, and **BDA-2-SA**, the differences of the water network upon ligand binding led to the observed high variance in thermodynamics. For the series of benzenesulfonamides (**4-NH2-SA**, **2-F-SA**, and **4-CH3-SA**), largely the same conclusions can be reached as with WaterFLAP: stabilization of a thermodynamically unfavorable water by the (protonated) amine group of **4-NH2-SA** contributes to its surprisingly low affinity.

## Discussion

Our results show that the computational modeling methods WaterFLAP and MobyWat provide sufficiently good explanations to get a deeper understanding of the structure and net thermodynamics of the associated water networks upon ligand binding. ITC measurements performed in D_2_O and H_2_O provided an experimental way to get an insight into the effect of the water network during ligand binding, and these results served also as a reference point for the computational methods. Earlier works can be found that discuss ITC results, binding mode analysis, and water network analysis for trypsin and CAII. Here, we aim to place our observations into the wider context of these efforts.

The binding of benzamidine-type ligands against trypsin was thoroughly studied over the years by the Klebe lab and other research groups in their efforts toward a deeper understanding of ligand binding thermodynamics, including the question of solvent effects. Two landmark papers were published on this model system in 2001. Dullweber and his colleagues examined relatively larger benzamidines vs trypsin using ITC and concluded that the local water structure presumably contributes to the heat capacity change^95^ and also observed the enthalpy–entropy compensation effect. The group followed up their work later by a comprehensive characterization of further analogs by ITC, X-ray crystallography, and molecular dynamics simulations,^96^ with both works reporting on the phenomenon of enthalpy–entropy compensation. The other important paper from 2001 is the work of Talhout and Engberts, in which the series of small benzamidines studied herein were first characterized with ITC.^44^ The authors primarily studied the temperature-dependence of the thermodynamics of ligand binding, observing a correlation between the polarity of the *para*-substituent and the inhibitory potency of the ligand and connecting this observation to bulk solvation effects. Members of this simpler benzamidine series have constituted the basis for further investigations. Gopal and his colleagues examined the binding of *para*-aminobenzamidine (**4-NH2-BA**) to trypsin in different water/methanol mixtures using ITC, highlighting the importance of solvation effects in binding thermodynamics while also observing the enthalpy–entropy compensation effect.^97^ Based on newly reported neutron diffraction structures and ITC-based thermodynamic binding signatures of unsubstituted benzamidine against trypsin, Schiebel and his colleagues highlighted the importance of water displacement and conformational flexibility for ligand binding.^67^

Our work complements these efforts by characterizing the binding thermodynamics of five smaller benzamidine analogs with ITC in both light and heavy water, taking inspiration from the approach introduced in the early 1990s in the works of Connelly et al.^20^ and Chervenak and Toone.^21^ The net thermodynamic signatures provided reference points for the validation of the water network prediction algorithms WaterFLAP and MobyWat and allowed us to observe the characteristics of ligand binding in this model system. Notably, the presence of a thermodynamically favorable (“happy”) water molecule in the binding site and its expulsion by the ligands explain the modest affinities (*K*_d_ values near or above 10 μM).

As for the second model system, a surprisingly early contribution by Binford and his colleagues (a calorimetric study of the binding of sulfonamides against human and bovine carbonic anhydrase isoforms) dates back to 1974 (no compounds from the present work were studied).^98^ A small series of fluorinated benzenesulfonamide ligands (including **2-F-SA**) vs bovine CAII were studied by Krishnamurthy with ITC, finding that the effects of fluorination upon ligand binding originated from three main sources: the Zn^2+^-sulfonamide interaction, the protein–ligand hydrogen bonds, and the contacts between the phenyl ring and CAII.^99^ In 2009, Scott performed ITC measurements for several benzenesulfonamides, including **2-F-SA**, **4-CH3-SA**, and **4-NH2-SA**, and discussed about the binding thermodynamics changing significantly between ligands with very similar substitutions, proposing water molecule displacement as an explanation for some cases.^43^ The other series in this work, heterocyclic aromatic sulfonamides, were studied most prominently by the Whitesides lab. Snyder and his colleagues used ITC with CAII and **TA-2-SA**, **TP-2-SA**, **BF-2-SA**, **BTA-2-SA**, **BDA-2-SA**, and **THBT-2-SA** to examine hydrophobic interactions thermodynamically and found that the differences in binding between ligands come from changes in the number and organization of water molecules in the active site in addition to the release of structured waters.^45^ WaterMap was also used to estimate the contribution from water to the free energy of binding.^45^ Breiten used **BTA-2-SA** and its fluorinated analogs specifically to examine the phenomenon of enthalpy–entropy compensation using ITC and concluded on the significant role of the waters surrounding the bound ligands.^100^ In further contributions, the Whitesides lab used benzenesulfonamides with variable-length alkyl and fluoroalkyl side chains to study ligand interactions with the “hydrophobic wall” of CAII, highlighting the role of nonoptimally hydrogen-bonded water molecules that hydrate the binding cavity.^101^ In the meantime, Morkunaite used a different series of sulfonamides (none from this work) to study various contributing factors of ligand binding, like pH dependence^102^ and the role of specific carbonic anhydrase isoforms using ITC.^103^

Similarly to the case of trypsin, our work complements these previous efforts by providing a qualitative analysis using water prediction methods and validating the findings against experimental ITC data in light and heavy water for two series of sulfonamide-type CAII inhibitors, expanding the research about the importance of water networks and their effect on protein-ligand binding thermodynamics. Notably, the CAII binding site contains thermodynamically unfavorable (“unhappy”) waters, which is consistent with the low *K*_d_ values and with the higher affinity compared to the investigated trypsin complexes, where the displaced binding site water molecules are predominantly thermodynamically favorable. A connection was also found between ΔΔ*H*_bind_ and the number of heteroatoms in the aromatic ring.^79^

By examining the difference of Δ*H*_bind_ in regular versus heavy water, we can evaluate the effects of ligand binding upon the water network of the respective binding sites. The comparative analysis of water networks in the X-ray complex structures and the differences of the binding enthalpy in D_2_O versus H_2_O showed a tendency that the magnitude of ΔΔ*H*_bind_ increases with the number of water molecules in the proximity of the ligand. While the majority of the ligands bind with a more favorable enthalpy in H_2_O than in D_2_O, we found three CAII ligands and one trypsin ligand showing the opposite behavior. This cannot be explained by the stabilization of the unbound state in D_2_O due to the uniformly 10% stronger H-bonds.^21,24^ We propose that the binding enthalpy of H_2_O (Δ*H*_bind_^H2O^) may vary in the binding site, and the enthalpy change (ΔΔ*H*_bind_) owing to the replacement of H_2_O by D_2_O may also vary depending on the protein binding site, as well as the structural features of the ligand. The variation of Δ*H*_bind_^H2O^ is analogous with the documented variation of Δ*G*_bind_^H2O^, the binding free energy of water molecules in the binding site. Additionally, there is a complex interplay of intrinsic processes that we cannot directly measure, best summarized as the vis-à-vis effect of enthalpy and entropy through their influence upon the conformational space. As such, to interpret the variation of ΔΔ*H*_bind_, we assume that the enthalpy difference of H-bonds before and after complex formation does not vary uniformly with the H_2_O to D_2_O change; rather, it is affected by the protein environment and the ligand. This assumption is in line with the different geometry dependence of the H-bond energy for H_2_O, compared to D_2_O.^104^

The findings from these measurements contribute to a deeper understanding of the thermodynamics of protein–ligand binding and have the potential to facilitate the identification of improved ligands in computer-aided drug design. In particular, a larger ΔΔ*H*_bind_ value can be considered as a “signaling light” for ligands whose on-target binding is affected more heavily by the local water network. Also, ligands that stabilize thermodynamically unfavorable water molecules in their binding pose were observed to lose some binding affinity in comparison to structurally closely related analogs. This gives a further dimension to the use of water network prediction algorithms during medicinal chemistry optimization, highlighting the importance of considering the water network that is formed upon ligand binding (in addition to the waters that are displaced during ligand binding).

## Conclusions

We investigated the impact of the water network on ligand–protein binding in two well-characterized model systems, relying on the solvent isotope effects when replacing regular water with heavy water. ITC measurements done in both H_2_O and D_2_O can be used to get an insight into the effect of the water network on the binding of a ligand. Our results, in combination with the available high-resolution protein–ligand complex structures, were used to validate two state-of-the-art computational water network prediction algorithms, WaterFLAP and MobyWat.

WaterFLAP and MobyWat were shown to predict the position of water molecules in accordance with the experimental structures. They assign Δ*G*_bind_ and Δ*H*_bind_ values, respectively, that help to interpret the net thermodynamic consequences of ligand binding. MobyWat uses explicit water molecular dynamics, which can predict all interactions, including those among water molecules that are not taken into account in methods focusing on solute–water interactions. Overall, water network prediction tools like WaterFLAP and MobyWat can be used to predict and score water molecules within the apo protein binding site and in the protein–ligand complexes to gain information about the thermodynamic stability of the water molecules to interpret ligand affinity variations and ultimately to guide further ligand optimization efforts. Notably, our results highlight the importance of accounting not only for the water molecules that are displaced upon ligand binding but also for the final water network that is formed during the process.

## Supporting Information Available

Structures (in SMILES format) and experimentally determined binding thermodynamic data of the studied trypsin and CAII inhibitors (XLSX)
Supplementary figures including the comparison of the predicted water positions by WaterFLAP and the experimental water positions from the X-ray diffraction and neutron diffraction structures in trypsin (Figure S1) and CAII (Figure S2); experimental water molecule positions in X-ray diffraction structures of CAII complexed with **2-F-SA**, **4-NH2-SA**, **BDA-2-SA**, **BTA-2-SA**, and **TA-2-SA** (Figure S3); predicted water molecule positions and their calculated Δ*G* values by WaterFLAP in CAII complexed with **4-CH3-SA**, **THBT-2-SA**, **BF-2-SA**, and **BTA-2-SA** (Figure S4); supplementary tables with WaterFLAP water perturbation analysis results for trypsin (Table S1) and CAII (Table S2); RMSD values of all protein atoms between apo and ligand-bound structures for trypsin and CAII (Table S3); calculated binding enthalpies of MobyWat-predicted water molecules and their displacement by ligands in trypsin (Table S4) and CAII (Table S5); success rates of water molecule prediction by MobyWat for trypsin and CAII (Table S6); ITC measurement conditions (Tables S7 and S8); ITC binding curves for all compounds in both H_2_O and D_2_O media (pages S14–S81) (PDF)
