# Supplementary material for: Effect of Water Networks On Ligand Binding: Computational Predictions vs Experiments
Source: J Chem Inf Model. 2024 Nov 22;64(23):8980–98. doi: 10.1021/acs.jcim.4c01291 (PMC11632780; doi:10.1021/acs.jcim.4c01291)
Supplement: Supplementary file 2 — ci4c01291_si_002.pdf [file ci4c01291_si_002.pdf]

The effect of water networks on ligand binding – computational predictions vs. experiments

Tibor Viktor Szalai,[1,2,3] Dávid Bajusz,[1,3] Rita Börzsei,[3,4] Balázs Zoltán Zsidó,[3,4] Janez Ilaš,[1,5] György G. Ferenczy,[1,3] Csaba Hetényi,[3,4] György M. Keserű[1,3,6,*]

[1] *Medicinal Chemistry Research Group, Drug Innovation Centre, HUN-REN Research Centre for Natural Sciences, Magyar tudósok krt. 2, 1117 Budapest, Hungary*

[2] *Department of Inorganic and Analytical Chemistry, Faculty of Chemical Technology and Biotechnology, Budapest University of Technology and Economics, Műegyetem rkp. 3., H-1111 Budapest, Hungary*

[3] *National Drug Research and Development Laboratory, Magyar tudósok krt. 2, 1117 Budapest, Hungary*

[4] *Pharmacoinformatics Unit, Department of Pharmacology and Pharmacotherapy, Medical School, University of Pécs, Szigeti út 12, H-7624 Pécs, Hungary*

[5] *Department of Pharmaceutical Chemistry, Faculty of Pharmacy, University of Ljubljana, Aškerčeva cesta 7, 1000 Ljubljana, Slovenia*

[6] *Department of Organic Chemistry and Technology, Faculty of Chemical Technology and Biotechnology, Budapest University of Technology and Economics, Műegyetem rkp. 3., H-1111 Budapest, Hungary*

*Corresponding author email: keseru.gyorgy@ttk.hu

Table of contents

1. Structural waters and waters predicted by WaterFLAP 2

2. MobyWat results 10

3. ITC results 13

3.1. ITC measurement conditions 13

3.2. ITC results for trypsin measurements in H_2O 15

3.3. ITC results for trypsin measurements in D_2O 27

3.4. ITC results for CAII measurements in H_2O 37

3.5. ITC results for CAII measurements in D_2O 64

4. References 83

1. Structural waters and waters predicted by WaterFLAP

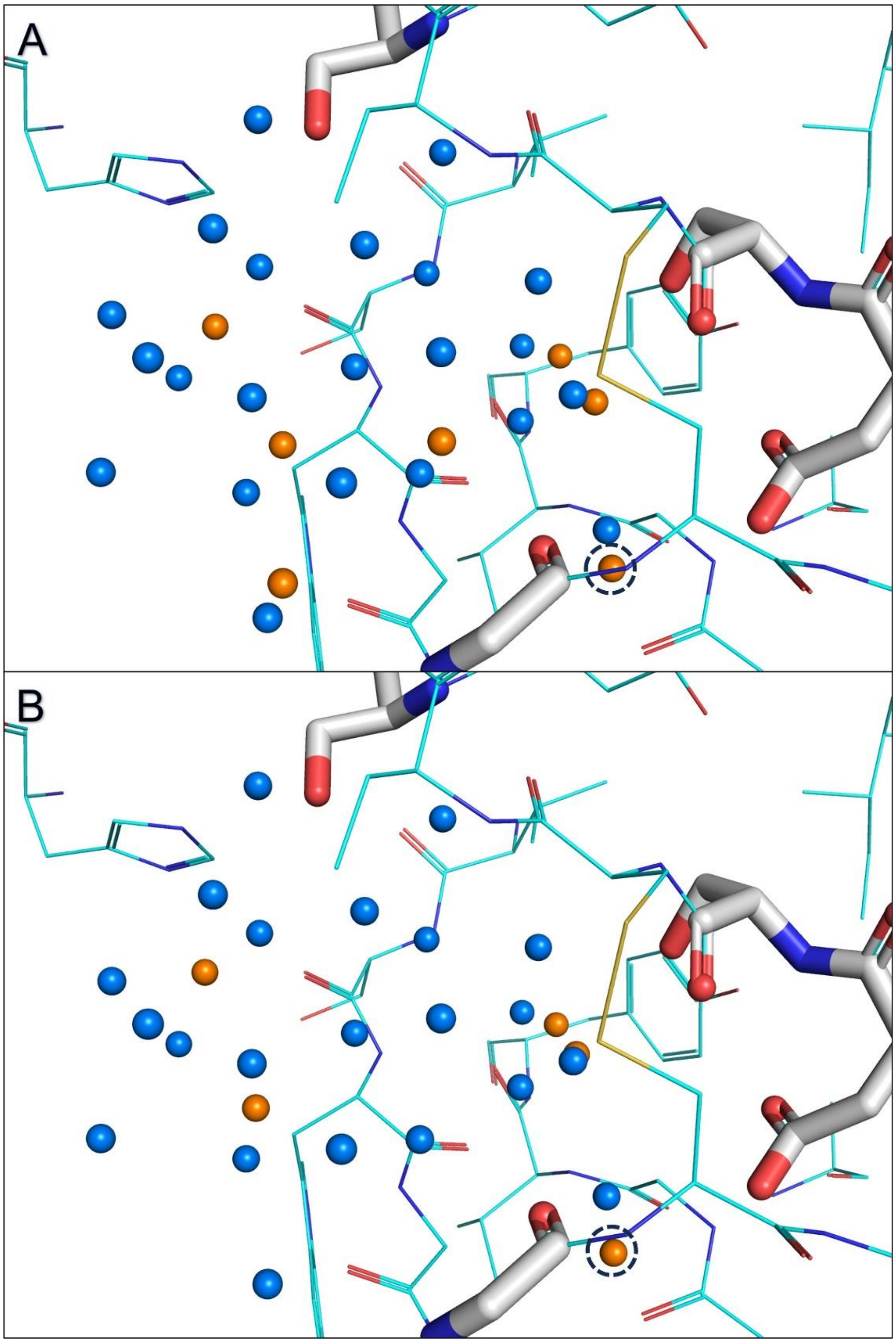

Supplementary Figure S1. Water molecules inside the binding site of trypsin. Water molecules predicted by WaterFLAP are highlighted with blue, structural waters are highlighted with orange. All

structural waters have a predicted water molecule in a 1.5 Å proximity. Circled structural water molecules are positionally correlating to the 'happy' water molecule predicted by WaterFLAP inside the trypsin binding site (Figure 3A).**(A)** Structural waters in the X-ray diffraction structure (PDB ID: 5MNF).[3] **(B)** Structural waters in the neutron diffraction structure (PDB ID: 5MNZ).[3]

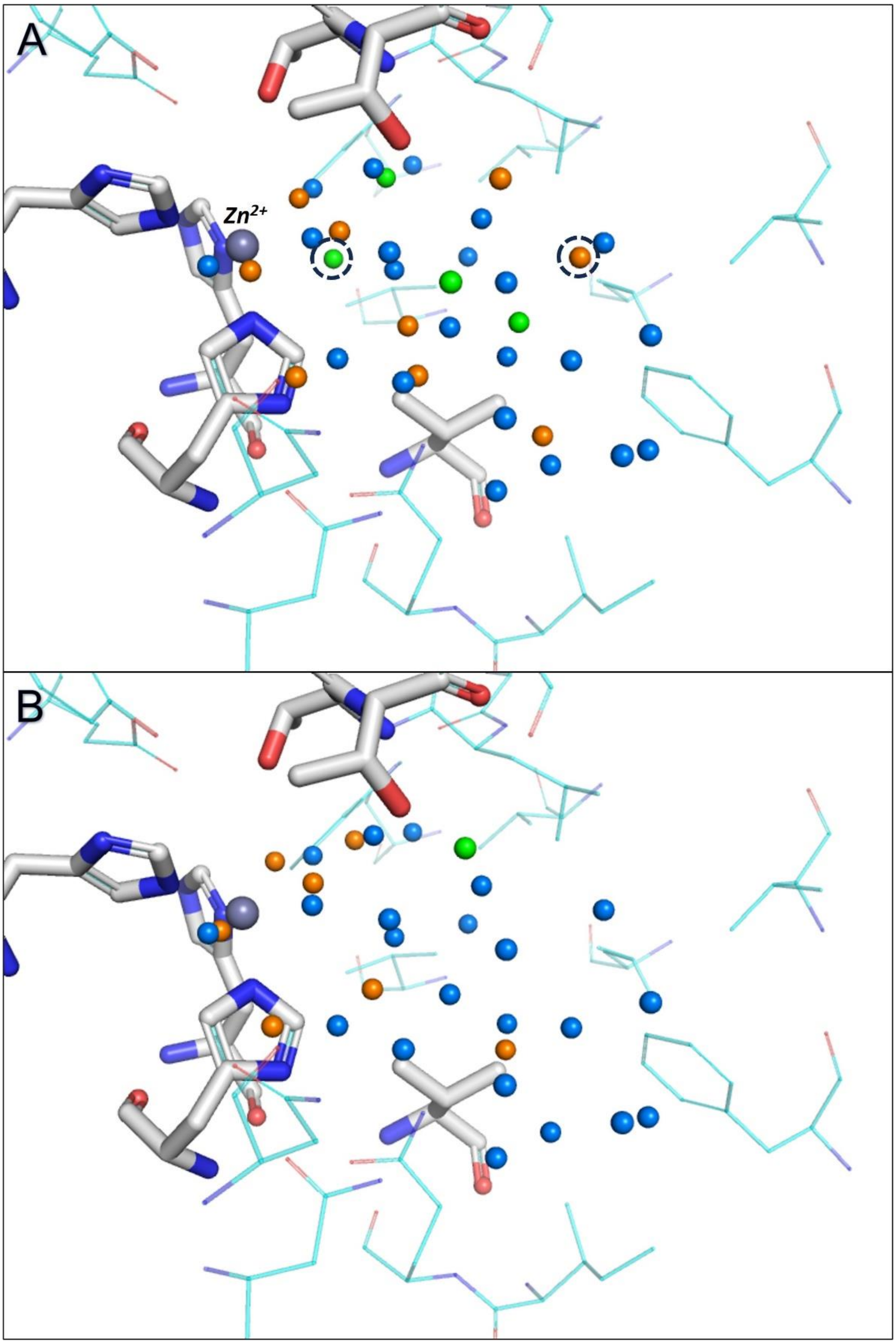

Supplementary Figure S2. Water molecules inside the binding site of carbonic anhydrase isozyme II (CAII). Water molecules predicted by WaterFLAP are highlighted with blue, structural waters are

highlighted with green and orange, with orange coloring meaning that the structural water molecule has a predicted water molecule in a 1.5 Å proximity. **(A)** Structural waters in the X-ray diffraction structure (PDB ID: 3KS3).[1] Circled structural water molecules are positionally correlating to the 'very unhappy' water molecules predicted by WaterFLAP inside the CAII binding site (Figure 7A). **(B)** Structural waters in the neutron diffraction structure (PDB ID: 4Q49).[2]

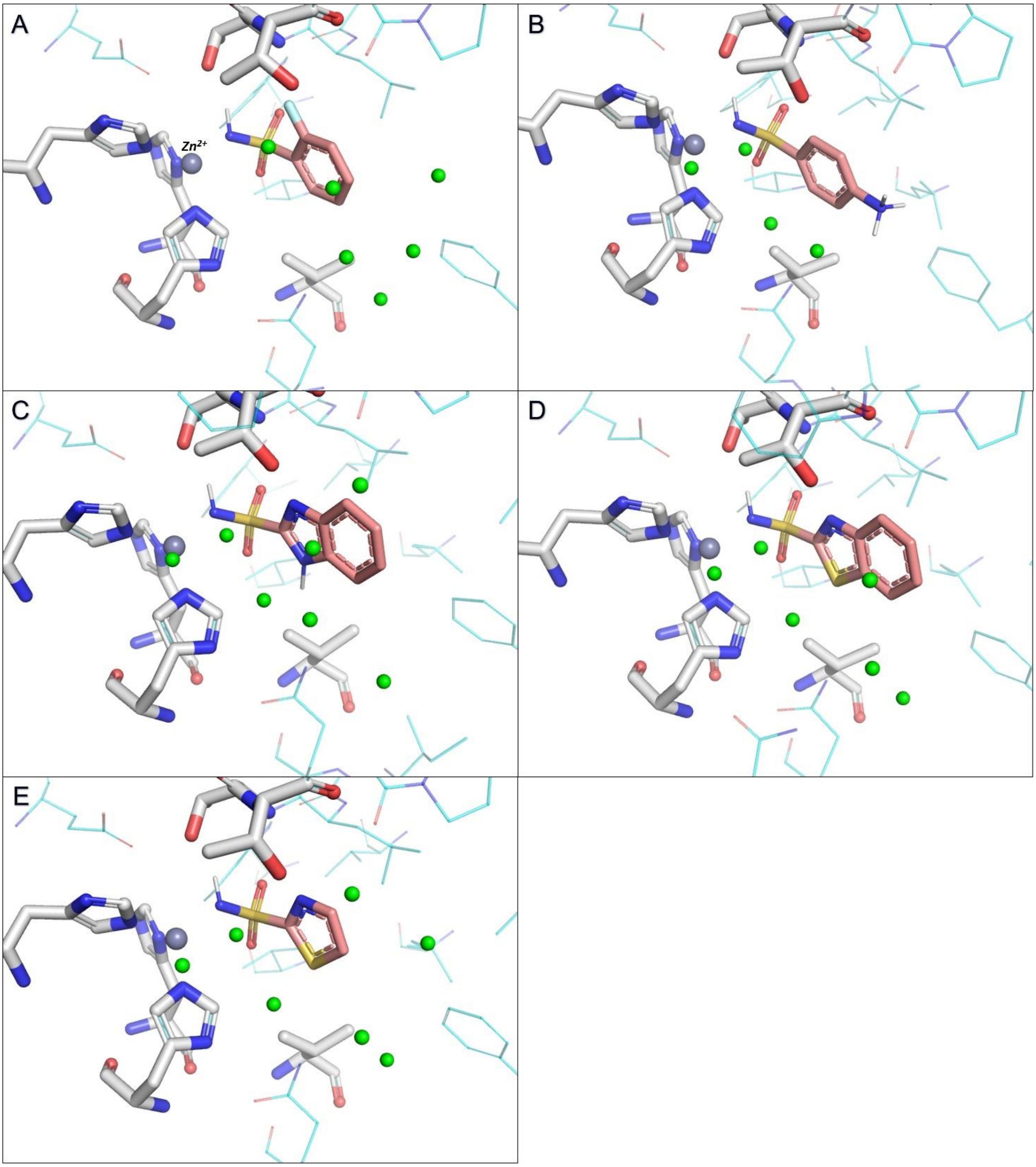

Supplementary Figure S3. Water molecules (highlighted with green) in experimental structures of CAII. X-ray structures of CAII complexed with **(A)** 2-F-SA (PDB ID: 2WEG),[4] **(B)** 4-NH2-SA (PDB ID: 6RL9),[5] **(C)** BDA-2-SA (PDB ID: 3S72), **(D)** BTA-2-SA (PDB ID: 3S73), **(E)** TA-2-SA (PDB ID: 3S77).[6]

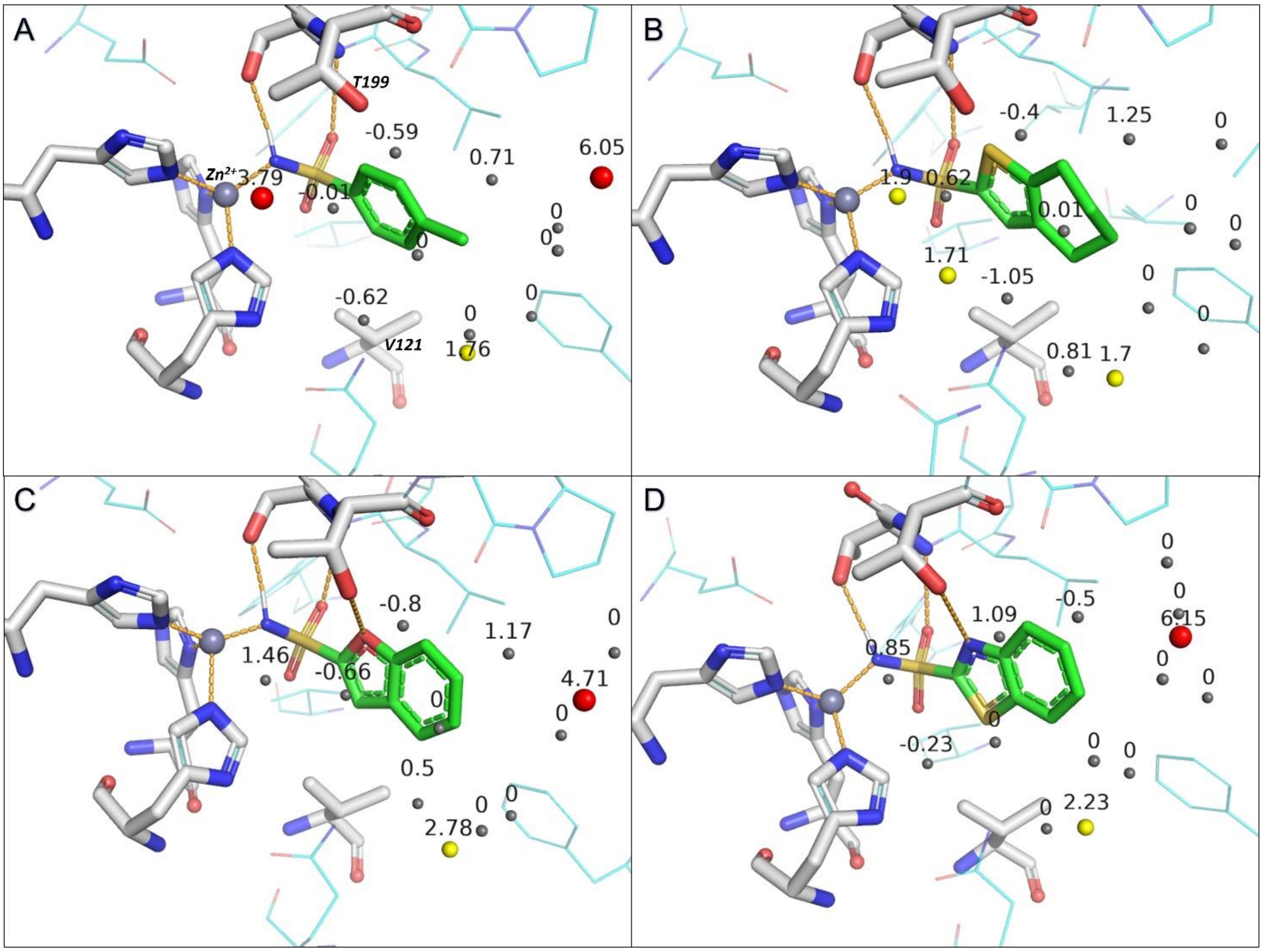

Supplementary Figure S4. Water molecules predicted by WaterFLAP inside the binding site of CAII, with all waters labelled with their calculated ΔG values from WaterFLAP. Hydrogen bonds are shown as orange dashed lines. Binding modes in CAII for (**A**) 4-CH3-SA, (**B**) THBT-2-SA, (**C**) BF-2-SA, (**D**) BTA-2-SA.

Table S1. Results from the water perturbation analysis with WaterFLAP compared with the $\Delta\Delta H_{bind}^{ITC}$ and $\Delta G_{bind}^{ITC,H_2O}$ values acquired from ITC for trypsin. $\sum_i^{perturbed} \Delta\Delta G_{bind,i}$ is the sum of the $\Delta\Delta G_{bind}$ values of the water molecules near the binding site after water perturbation analysis, $\sum_j^{displaced} \Delta G_{bind,j}$ is the sum of the ΔG_{bind} values of the displaced water molecules upon ligand binding. $\Delta\Delta H_{bind}^{ITC}$ values larger than 1 kcal/mol are in bold.

Ligand	$\sum_i^{perturbed} \Delta\Delta G_{bind,i}$ [kcal/mol]	$-\sum_j^{displaced} \Delta G_{bind,j}$ [kcal/mol]	ΔG_{waters}* [kcal/mol]	$\Delta G_{bind}^{ITC,H_2O}$ [kcal/mol]	$\Delta\Delta H_{bind}^{ITC}$ [kcal/mol]
BA	1.64	3.00	2.32	-6.39±0.03	0.42±0.16
4-NH2-BA	-3.21	2.67	-0.27	-6.92±0.05	0.46±0.21
4-CONH2-BA	-1.62	2.67	0.53	-5.62±0.11	0.00±1.14
4-CH3-BA	1.04	2.67	1.86	-6.70±0.03	**1.42±0.14**
4-OCH3-BA	7.18	2.67	4.93	-6.06±0.03	-0.09±0.44

*$\Delta G_{waters} = \frac{1}{2}\left(\sum_i^{perturbed} \Delta\Delta G_{bind,i} - \sum_j^{displaced} \Delta G_{bind,j}\right)$

Table S2. Results from the water perturbation analysis with WaterFLAP compared with the $\Delta\Delta H_{bind}^{ITC}$ and $\Delta G_{bind}^{ITC,H_2O}$ values acquired from ITC for CAII. $\sum_i^{perturbed} \Delta\Delta G_{bind,i}$ is the sum of the $\Delta\Delta G_{bind}$ values of the water molecules near the binding site after water perturbation analysis, $\sum_j^{displaced} \Delta G_{bind,j}$ is the sum of the ΔG_{bind} values of the displaced water molecules upon ligand binding. $\Delta\Delta H_{bind}^{ITC}$ values larger than 1 kcal/mol (in absolute value) are in bold.

Ligand	$\sum_i^{perturbed} \Delta\Delta G_{bind,i}$ [kcal/mol]	$-\sum_j^{displaced} \Delta G_{bind,j}$ [kcal/mol]	ΔG_{waters}* [kcal/mol]	$\Delta G_{bind}^{ITC,H_2O}$ [kcal/mol]	$\Delta\Delta H_{bind}^{ITC}$ [kcal/mol]
4-NH2-SA	-1.08	-8.38	-4.73	-7.53±0.05	0.25±0.22
2-F-SA	9.01	-8.38	0.32	-8.92±0.12	**-1.03±0.20**
4-CH3-SA	4.68	-8.38	-1.85	-8.92±0.10	**1.12±0.17**
TP-2-SA	4.67	-8.38	-1.86	-8.79±0.10	0.15±0.18
TA-2-SA	-0.09	-8.38	-4.24	-9.92±0.07	**1.78±0.11**
BTA-2-SA	7.02	-8.23	-0.61	-12.00±0.39	**5.80±0.08**
BF-2-SA	-2.88	-16.11	-9.50	-11.05±0.36	**-2.80±0.12**
BDA-2-SA	7.29	-8.23	-0.47	-8.89±0.04	**3.35±0.13**
THBT-2-SA	5.91	-16.11	-5.10	-10.60±0.14	-0.64±0.09

*$\Delta G_{waters} = \frac{1}{2}\left(\sum_i^{perturbed} \Delta\Delta G_{bind,i} - \sum_j^{displaced} \Delta G_{bind,j}\right)$

Table S3. All protein atom RMSD between apo and ligand bound structures.

Trypsin; reference 5MNZ		Carbonic anhydrase II; reference 3GZ0	
PDB code	RMSD (Å)	PDB code	RMSD (Å)
5MO0	0.068	2WEG	0.328
7WA2	0.157	6RL9	0.356
3GY4	0.177	4YXI	0.254
1S0R	0.283	3S71	0.359
		3S72	0.312
		3S73	0.337
		3S77	0.378
		3S78	0.386

2. MobyWat results

Table S4. The calculated binding enthalpies of MobyWat-predicted individual water molecules to~~The binding enthalpies of individual water molecules to~~ trypsin and their displacement by **BA** derivatives.

Water ID	ΔH_b (kcal/mol)	Water serial number	BA	4-NH2-BA	4-CONH2-BA	4-CH3-BA	4-OCH3-BA
226	-22.69	W1					
225	-18.99	W2					
228	-14.14	W3	X[a]	X	X	X	X
227	-13.90	W4			X		
234	-9.15	W5					
231	-7.01	W6	X	X	X	X	X
236	-6.43	W7	X	X		X	X
230	-6.26	W8	X	X	X	X	X
243	-5.73	W9	X	X	X	X	X
233	-5.10	W10			X		
237	-2.74	W11					
240	-2.28	W12					
235	-0.21	W13			X		
242	0.07	W14					
229	0.11	W15	X	X	X	X	X
241	0.75	W16			X	X	X
238	0.78	W17	X	X	X	X	X
239	1.26	W18					
244	1.63	W19			X		
232	4.53	W20	X	X	X	X	X
Number of displaced waters			8	8	12	9	9

[a] A water molecule was considered as displaced when it was within 1.75 Å of a ligand atom. The displacement is marked with an X.

Table S5. The calculated binding enthalpies of MobyWat-predicted individual water molecules to ~~The binding enthalpies of individual water molecules to~~ CAII ~~calculated by MobyWat~~ and their displacement by **SA** derivatives.

Water ID	ΔHb (kcal/mol)	Water serial number	4-NH2-SA	2-F-SA	4-CH3-SA	TP-2-SA	TA-2-SA	BTA-2-SA	BF-2-SA	BDA-2-SA	THBT-2-SA
262	-26.01	W1					X	X	X	X	X
268	-4.90	W2									
273	-2.24	W3	X[a]				X				
264	-2.05	W4	X	X	X	X			X		
271	-0.93	W5	X	X	X			X	X	X	X
274	-0.16	W6			X			X	X	X	X
266	0.17	W7									
270	0.71	W8	X	X	X			X	X	X	X
265	1.41	W9	X	X	X		X	X	X	X	X
269	1.54	W10	X	X	X	X	X	X	X	X	X
267	3.73	W11	X	X	X	X	X	X	X	X	X
272	4.23	W12			X	X	X	X	X		X
275	6.96	W13	X	X	X	X	X	X	X	X	X
263	7.15	W14	X	X	X	X	X	X	X	X	X
Number of displaced waters			9	8	10	6	8	10	11	9	10

[a] A water molecule was considered as displaced when it was within 1.75 Å of a ligand atom. The displacement is marked with an X.

Table S6. Calculated success rates for interface and surface water prediction by MobyWat for the holo and apo carbonic anhydrase and trypsin systems.

Ligand	Success rate (%)
4-NH2-SA	100
2-F-SA	80
4-CH3-SA	100
TP-2-SA	83.33
TA-2-SA	75*
BTA-2-SA	100
BF-2-SA	87.5
BDA-2-SA	100
BA	100
4-NH2-BA	100
4-OCH3-BA	83.3
Apo trypsin	87.5
Apo carbonic anhydrase	100

*The apparently lower success rate is based on the comparison with only 4 interface waters in the experimental structures used as references, and therefore, missing a single water position resulted in a 25 % drop of SR in this case.

3. ITC results

3.1. ITC measurement conditions

Table S7. Conditions for ITC measurements with CAII

Measurements done in H_2O with CAII					
Ligand	Ligand concentration [µM]	Protein concentration [µM]	Injection program [µl]	Time between injections [s]	Number of measurements
4-NH2-SA	1000	100	0.4 + 18×2	180	3
2-F-SA	500	50	0.4 + 18×2	180	4
4-CH3-SA	500	50	0.4 + 18×2	180	3
TP-2-SA	500	50	0.4 + 18×2	180	3
TA-2-SA	500	50	0.4 + 18×2	180	3
BTA-2-SA	1000	100	0.4 + 24×1	180	3
BF-2-SA	500, 1000	50, 100	0.4 + 18×2	180	3
BDA-2-SA	500	50	0.4 + 18×2	180	3
THBT-2-SA	500	50	0.4 + 18×2	180	2
**Measurements done in D_2O with CAII**					
Ligand	Ligand concentration [µM]	Protein concentration [µM]	Injection program [µl]	Time between injections [s]	Number of measurements
4-NH2-SA	1000	84.8, 86.1	0.4 + 18×2	150	2
2-F-SA	500	46.5	0.4 + 18×2	150	2
4-CH3-SA	500	46.5	0.4 + 18×2	150	2
TP-2-SA	500	46.5, 55.1	0.4 + 18×2	180	2
TA-2-SA	500	55.1	0.4 + 18×2	180	2
BTA-2-SA	999	129	0.4 + 24×1.3	180	2
BF-2-SA	500	55.1	0.4 + 18×2	150	2
BDA-2-SA	500	55.9	0.4 + 18×2	150	3
THBT-2-SA	500	55.1	0.4 + 18×2	180	2

Table S8. Conditions for trypsin ITC measurements

**Measurements done in H_2O with trypsin**					
Ligand	Ligand concentration [mM]	Protein concentration [µM]	Injection program [µl]	Time between injections [s]	Number of measurements
BA	5	150	1 + 2×2 + 18×5	300	2
4-NH2-BA	5	150	1 + 2×2 + 18×5	300	2
4-CONH2-BA	5	150	1 + 2×2 + 18×5	300	4
4-CH3-BA	5	150	1 + 2×2 + 18×5	300	2
4-OCH3-BA	5	150	1 + 2×2 + 18×5	300	2
**Measurements done in D_2O with trypsin**					
Ligand	Ligand concentration [mM]	Protein concentration [µM]	Injection program [µl]	Time between injections [s]	Number of measurements
BA	5	150	1 + 8×5 + 16×10 + 4×20	300	2
4-NH2-BA	5	150	1 + 2×2 + 18×5	300	2
4-CONH2-BA	5	150	1 + 8×5 + 16×10 + 4×20	300	2
4-CH3-BA	5	150	1 + 2×2 + 18×5	300	2
4-OCH3-BA	5	150	1 + 8×5 + 16×10 + 4×20	300	2

3.2. ITC results for trypsin measurements in H_2O

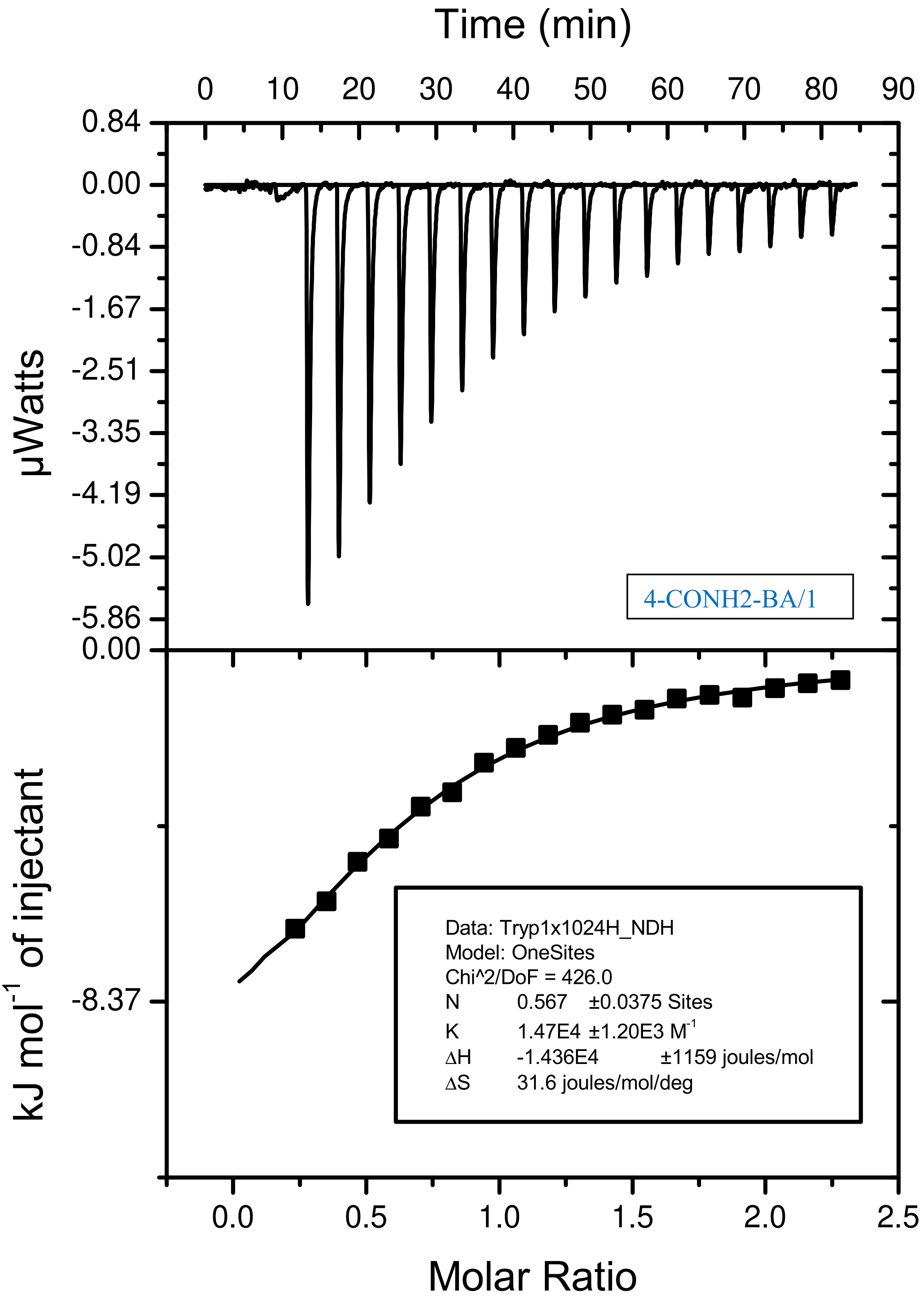

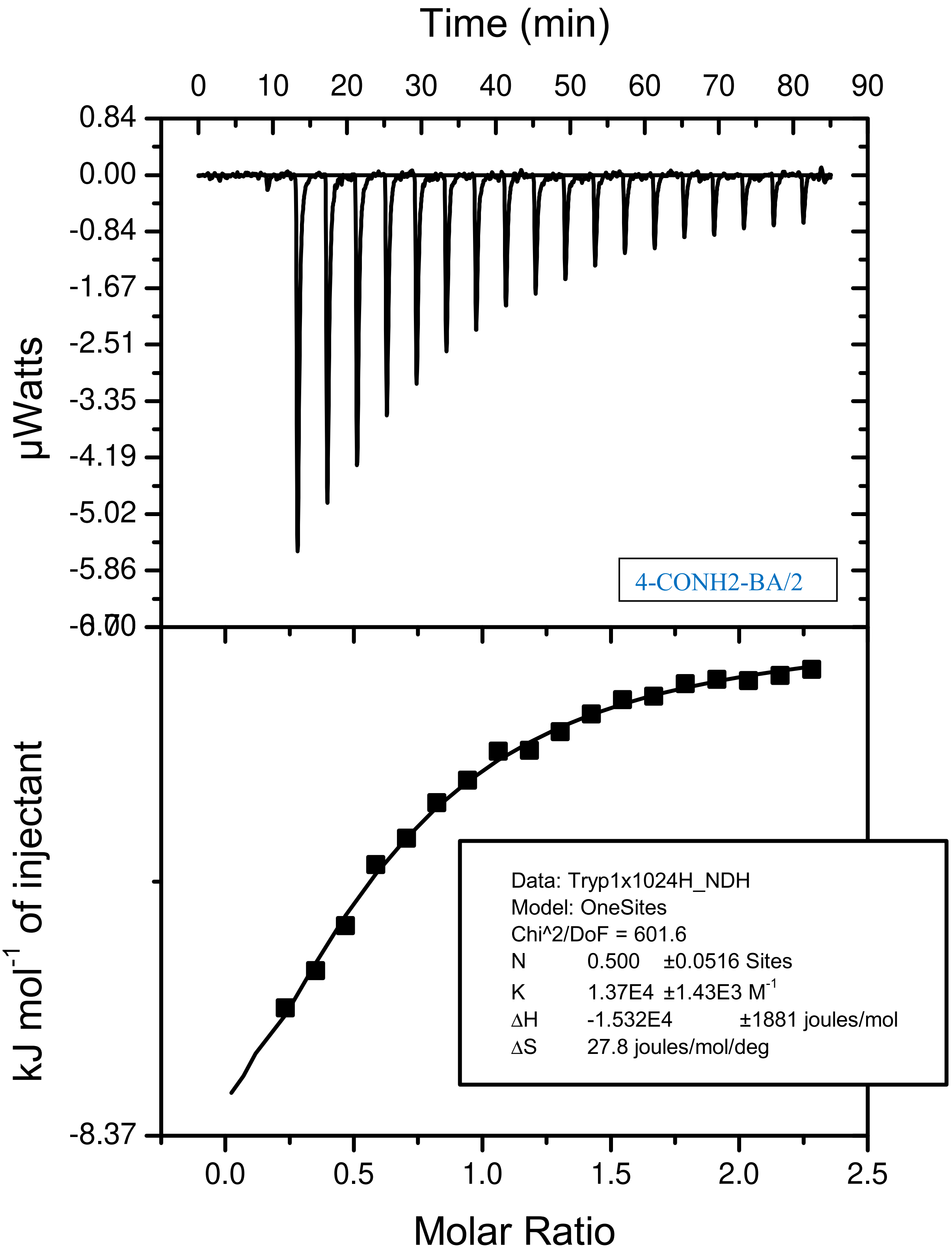

Time (min)
0
10
20
30
40
50
60
70
80
90
0.84
0.00
-0.84
-1.67
-2.51
-3.35
-4.19
-5.02
-5.86
-6.70
μWatts
4-CONH2-BA/2
kJ mol-1 of injectant
-8.37
Data: Tryp1x1024H_NDH
Model: OneSites
Chi^2/DoF = 601.6
N 0.500 ±0.0516 Sites
K 1.37E4 ±1.43E3 M-1
ΔH -1.532E4 ±1881 joules/mol
ΔS 27.8 joules/mol/deg
0.0
0.5
1.0
1.5
2.0
2.5
Molar Ratio

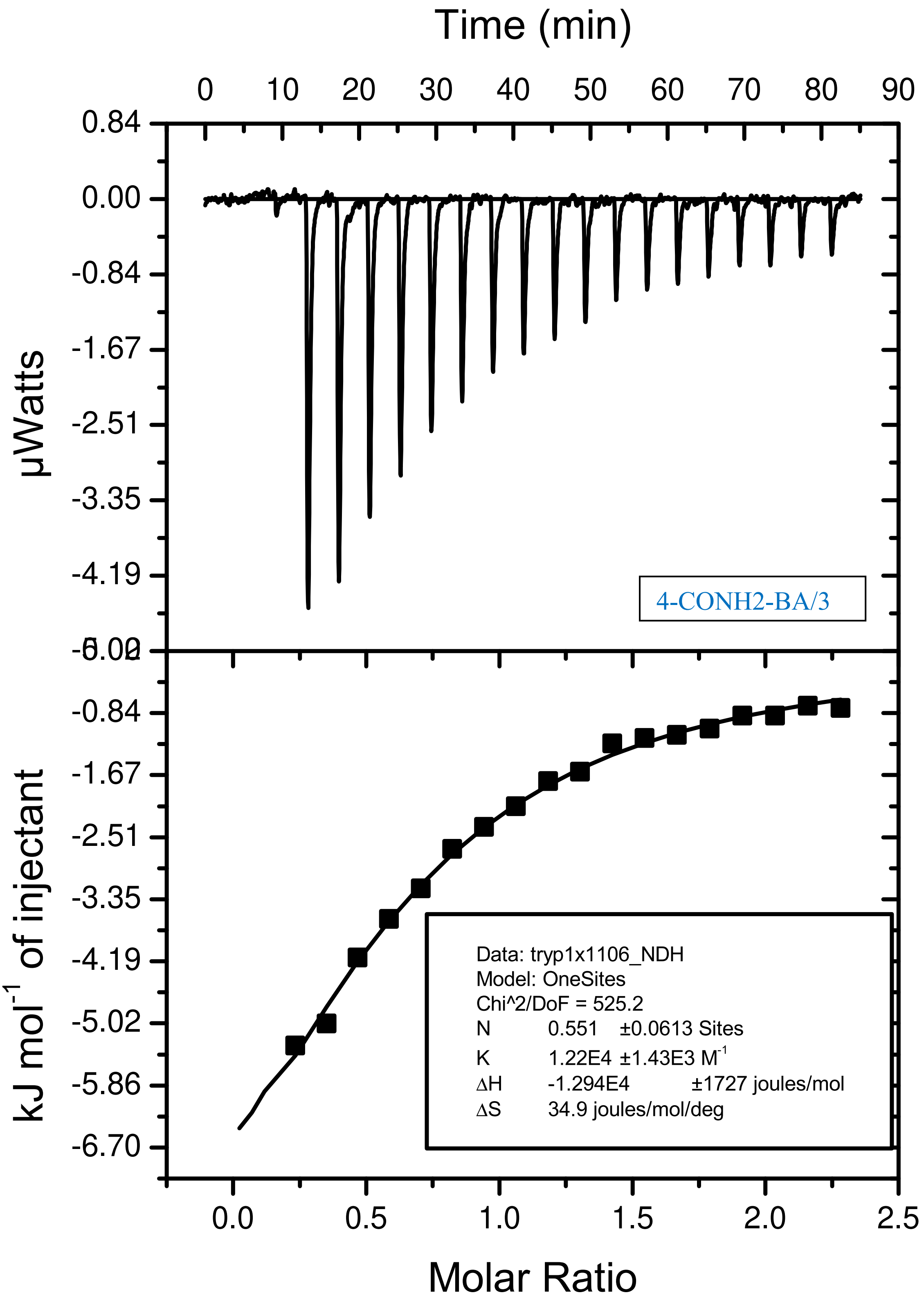

Time (min)
0
10
20
30
40
50
60
70
80
90
0.84
0.00
-0.84
-1.67
-2.51
-3.35
-4.19
-5.02
μWatts
4-CONH2-BA/3
-0.84
-1.67
-2.51
-3.35
-4.19
-5.02
-5.86
-6.70
kJ mol-1 of injectant
Data: tryp1x1106_NDH
Model: OneSites
Chi^2/DoF = 525.2
N 0.551 ±0.0613 Sites
K 1.22E4 ±1.43E3 M-1
ΔH -1.294E4 ±1727 joules/mol
ΔS 34.9 joules/mol/deg
0.0
0.5
1.0
1.5
2.0
2.5
Molar Ratio

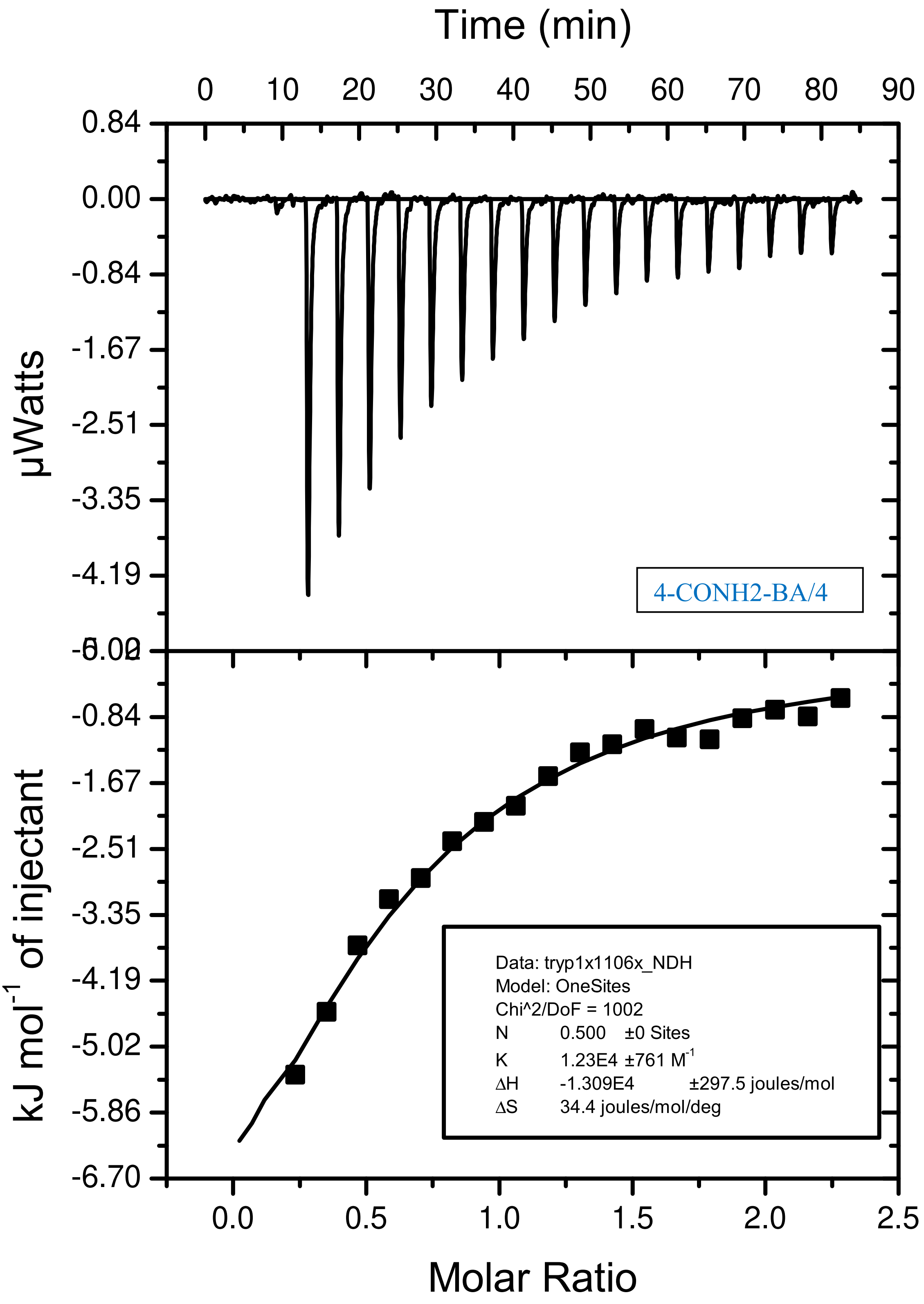

Time (min)
0 10 20 30 40 50 60 70 80 90
µWatts
0.84
0.00
-0.84
-1.67
-2.51
-3.35
-4.19
-5.02
4-CONH2-BA/4
kJ mol-1 of injectant
-0.84
-1.67
-2.51
-3.35
-4.19
-5.02
-5.86
-6.70
Data: tryp1x1106x_NDH
Model: OneSites
Chi^2/DoF = 1002
N 0.500 ±0 Sites
K 1.23E4 ±761 M-1
ΔH -1.309E4 ±297.5 joules/mol
ΔS 34.4 joules/mol/deg
0.0 0.5 1.0 1.5 2.0 2.5
Molar Ratio

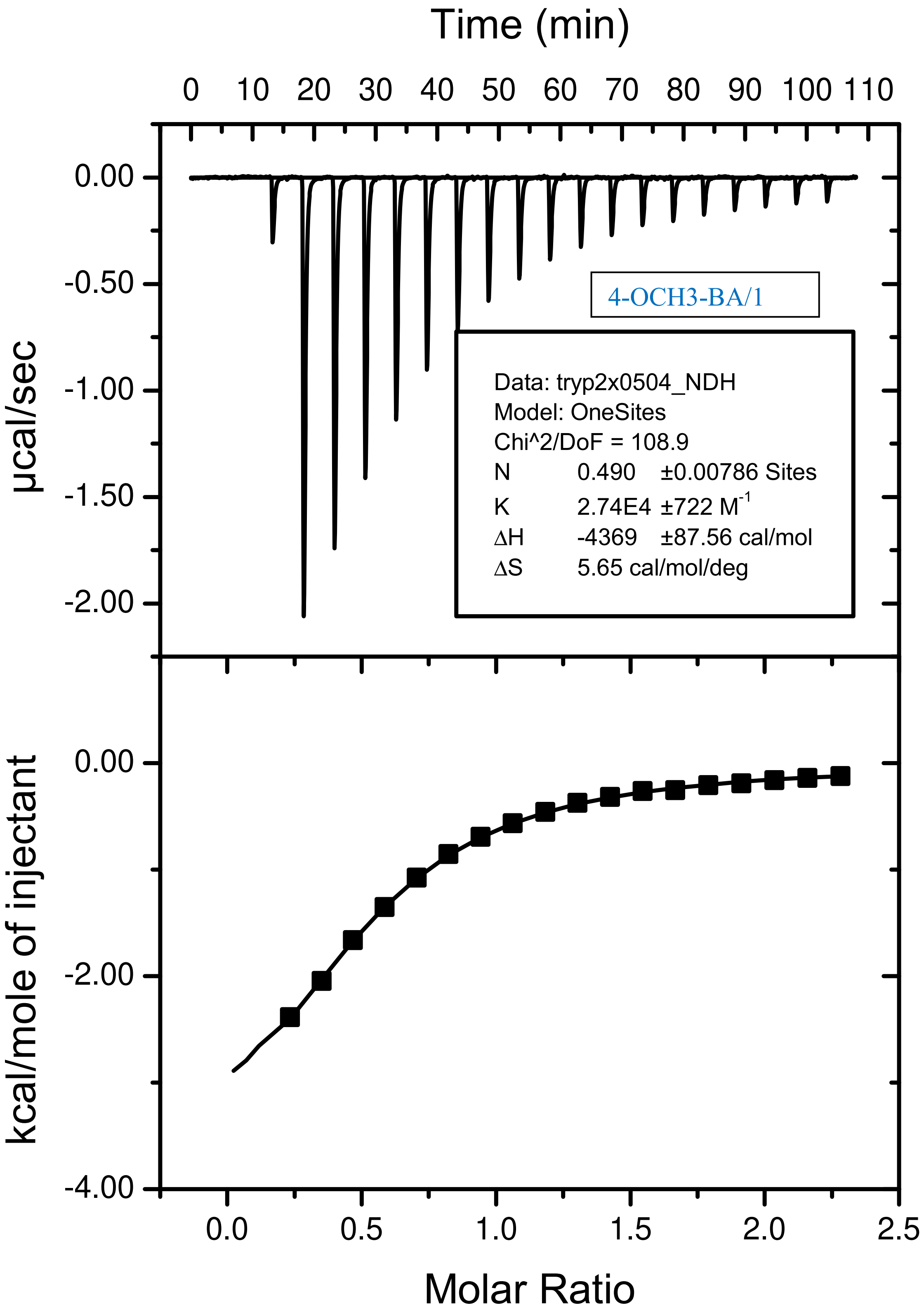
Time (min)
0 10 20 30 40 50 60 70 80 90 100 110
0.00
-0.50
-1.00
-1.50
-2.00
µcal/sec
4-OCH3-BA/1
Data: tryp2x0504_NDH
Model: OneSites
Chi^2/DoF = 108.9
N 0.490 ±0.00786 Sites
K 2.74E4 ±722 M-1
ΔH -4369 ±87.56 cal/mol
ΔS 5.65 cal/mol/deg
0.00
-2.00
-4.00
kcal/mole of injectant
0.0 0.5 1.0 1.5 2.0 2.5
Molar Ratio

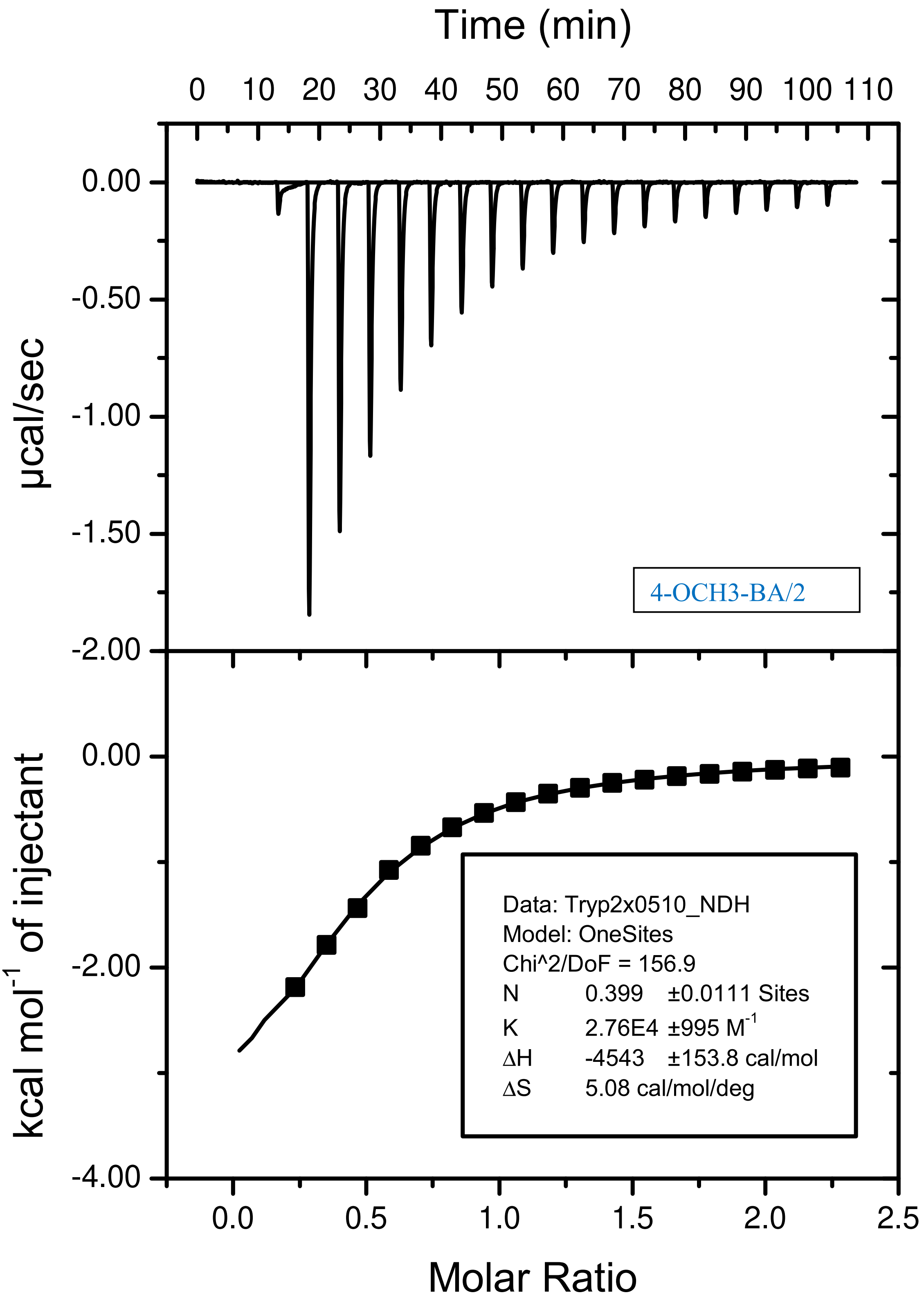

Time (min)
0 10 20 30 40 50 60 70 80 90 100 110
µcal/sec
0.00
-0.50
-1.00
-1.50
-2.00
4-OCH3-BA/2
kcal mol-1 of injectant
0.00
-2.00
-4.00
Data: Tryp2x0510_NDH
Model: OneSites
Chi^2/DoF = 156.9
N 0.399 ±0.0111 Sites
K 2.76E4 ±995 M-1
ΔH -4543 ±153.8 cal/mol
ΔS 5.08 cal/mol/deg
0.0 0.5 1.0 1.5 2.0 2.5
Molar Ratio

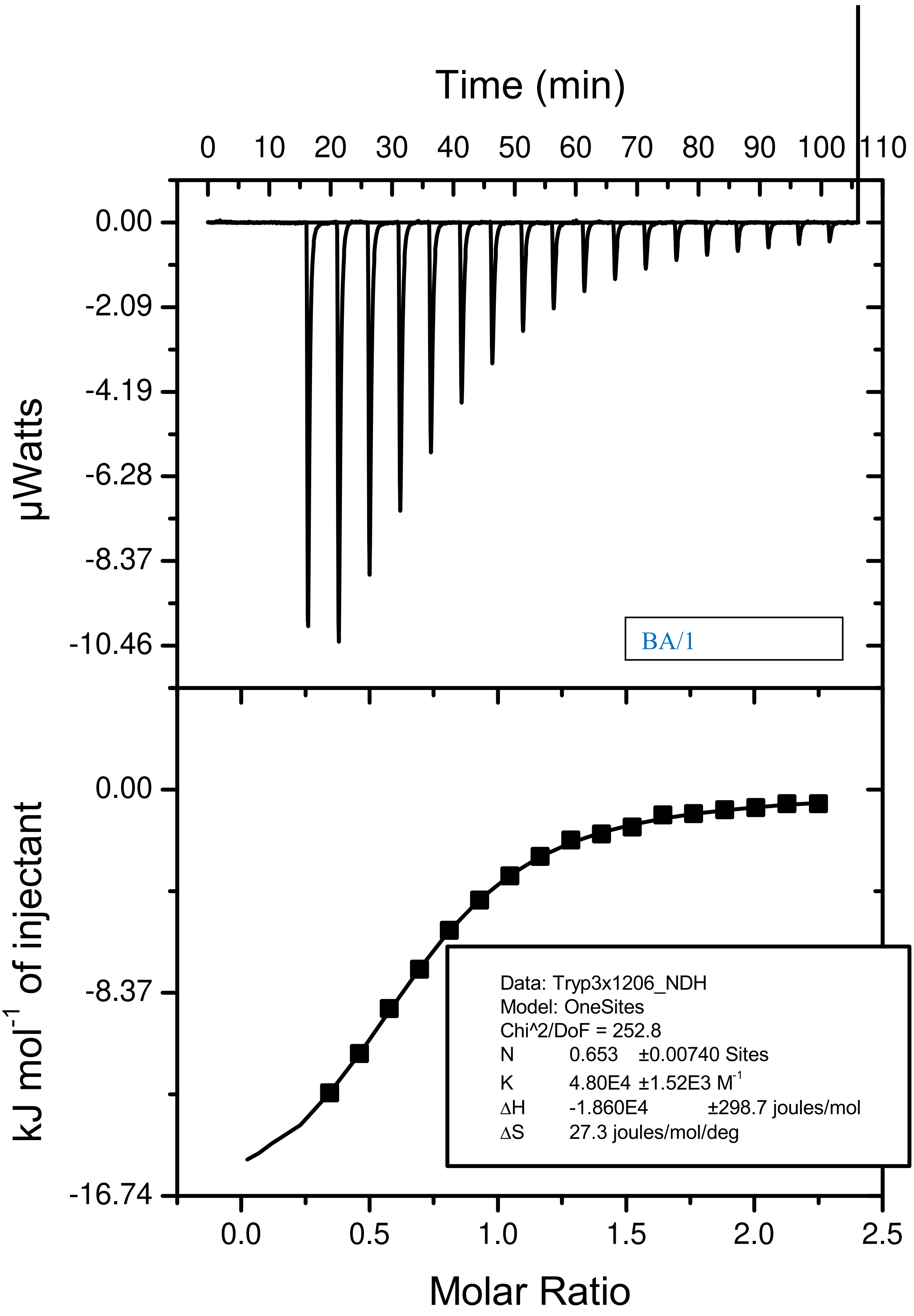

Time (min)
0 10 20 30 40 50 60 70 80 90 100 110
µWatts
0.00
-2.09
-4.19
-6.28
-8.37
-10.46
BA/1
kJ mol-1 of injectant
0.00
-8.37
-16.74
Data: Tryp3x1206_NDH
Model: OneSites
Chi^2/DoF = 252.8
N 0.653 ±0.00740 Sites
K 4.80E4 ±1.52E3 M-1
ΔH -1.860E4 ±298.7 joules/mol
ΔS 27.3 joules/mol/deg
0.0 0.5 1.0 1.5 2.0 2.5
Molar Ratio

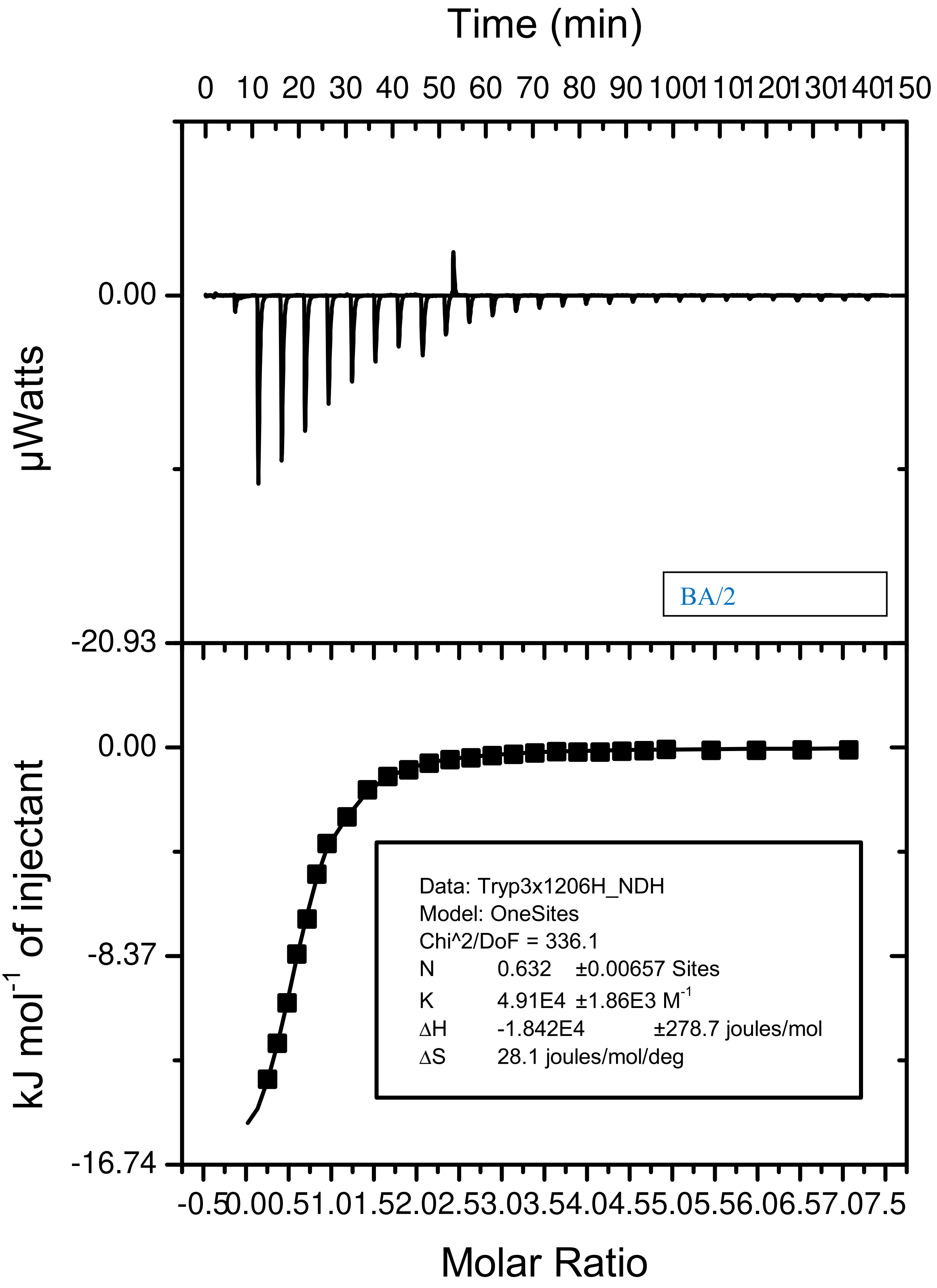

Time (min)
0 10 20 30 40 50 60 70 80 90 100 110 120 130 140 150
0.00
-20.93
µWatts
BA/2
0.00
-8.37
-16.74
kJ mol-1 of injectant
Data: Tryp3x1206H_NDH
Model: OneSites
Chi^2/DoF = 336.1
N 0.632 ±0.00657 Sites
K 4.91E4 ±1.86E3 M-1
ΔH -1.842E4 ±278.7 joules/mol
ΔS 28.1 joules/mol/deg
-0.5 0.0 0.5 1.0 1.5 2.0 2.5 3.0 3.5 4.0 4.5 5.0 5.5 6.0 6.5 7.0 7.5
Molar Ratio

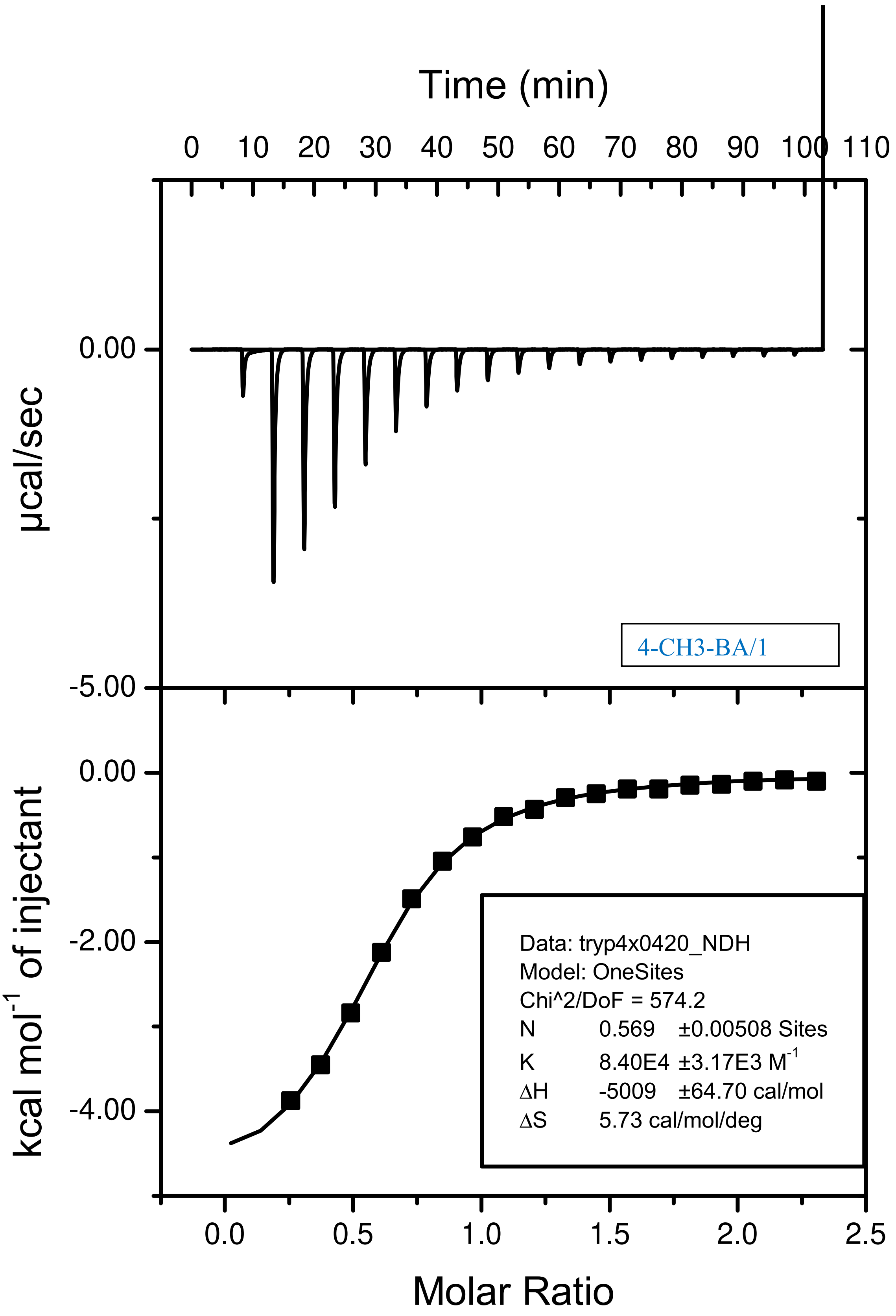
Time (min)
0 10 20 30 40 50 60 70 80 90 100 110
0.00
-5.00
µcal/sec
4-CH3-BA/1
0.00
-2.00
-4.00
kcal mol-1 of injectant
Data: tryp4x0420_NDH
Model: OneSites
Chi^2/DoF = 574.2
N 0.569 ±0.00508 Sites
K 8.40E4 ±3.17E3 M-1
ΔH -5009 ±64.70 cal/mol
ΔS 5.73 cal/mol/deg
0.0 0.5 1.0 1.5 2.0 2.5
Molar Ratio

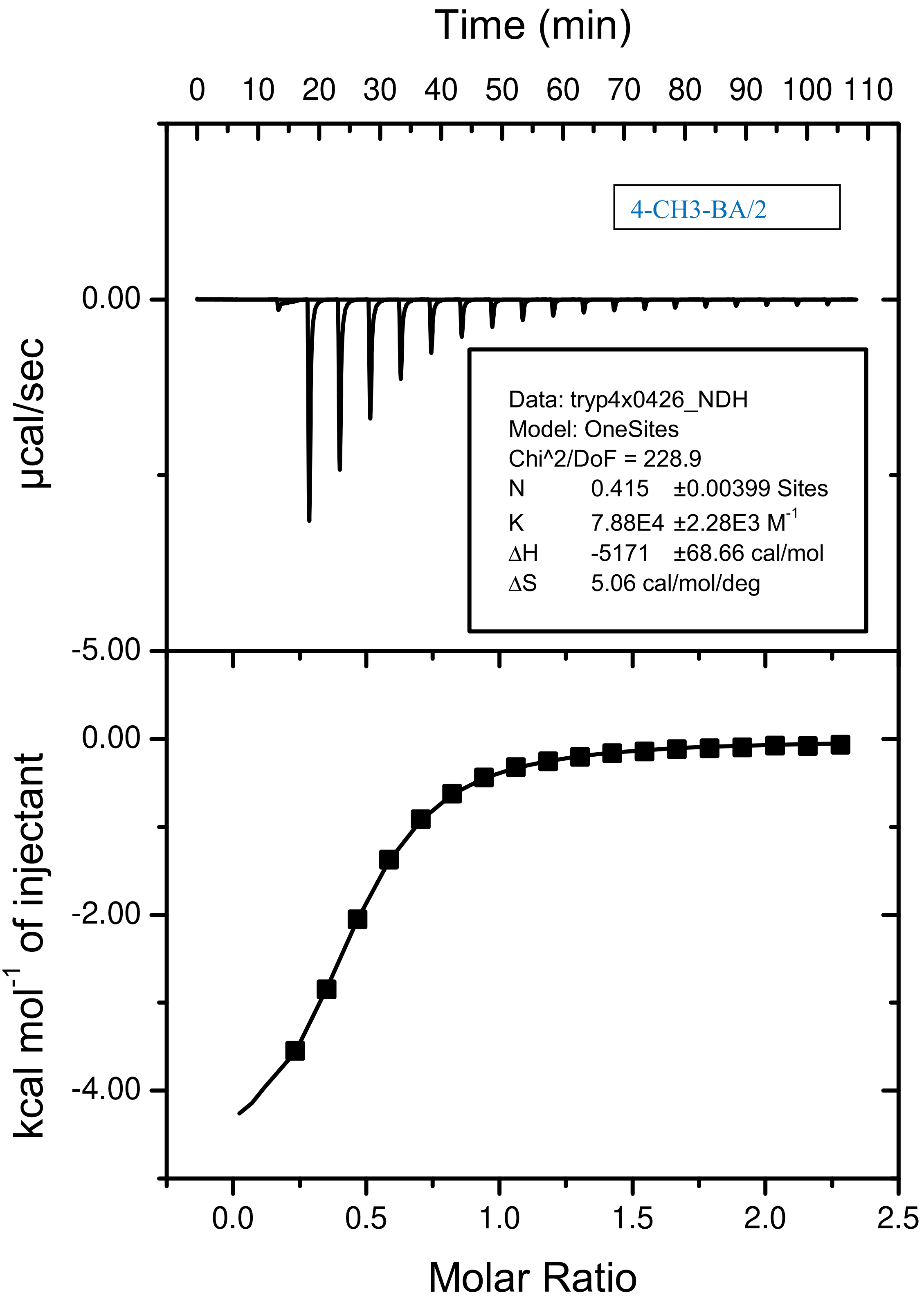
Time (min)
0 10 20 30 40 50 60 70 80 90 100 110
4-CH3-BA/2
0.00
-5.00
µcal/sec
Data: tryp4x0426_NDH
Model: OneSites
Chi^2/DoF = 228.9
N 0.415 ±0.00399 Sites
K 7.88E4 ±2.28E3 M-1
ΔH -5171 ±68.66 cal/mol
ΔS 5.06 cal/mol/deg
0.00
-2.00
-4.00
kcal mol-1 of injectant
0.0 0.5 1.0 1.5 2.0 2.5
Molar Ratio

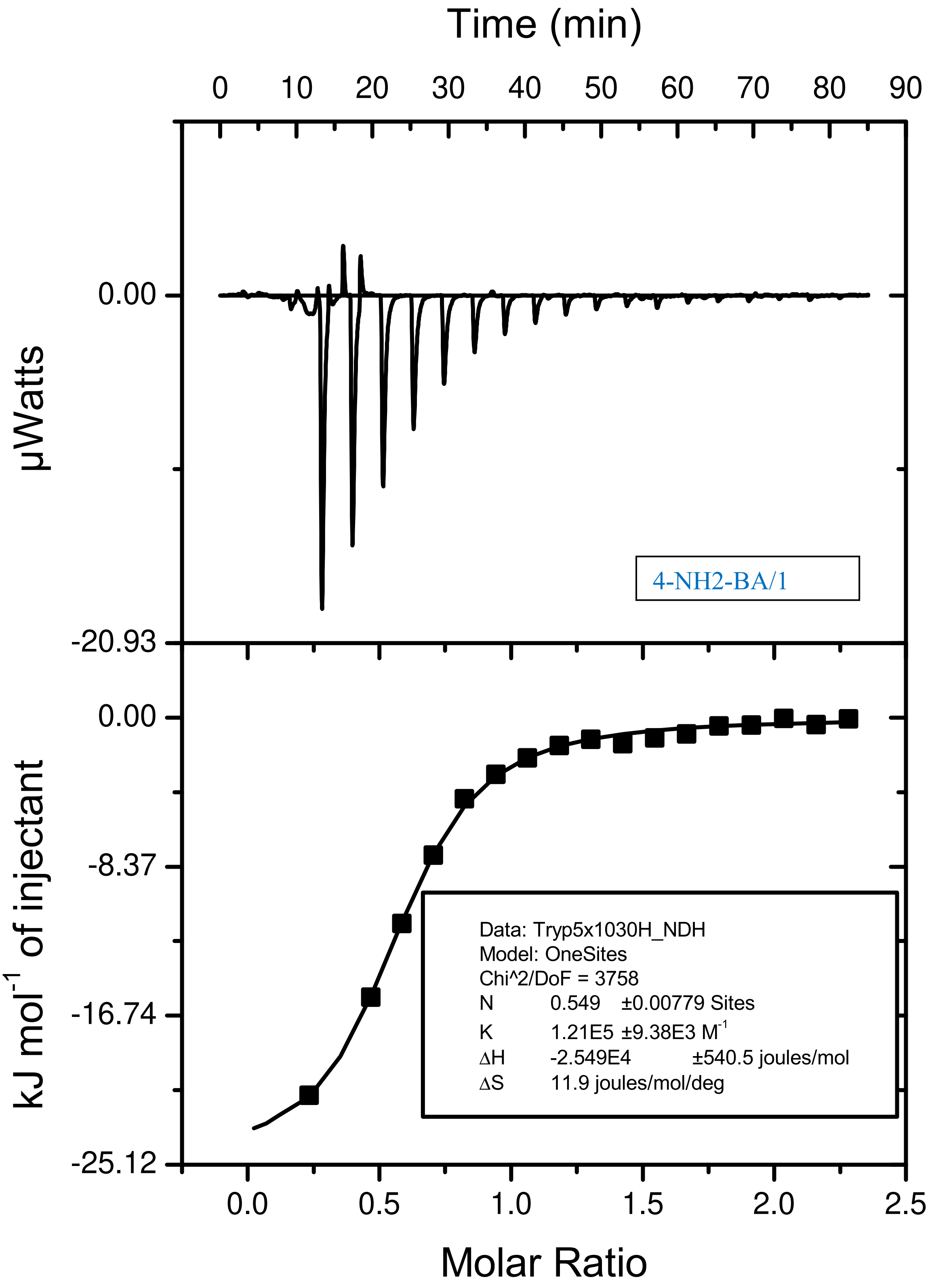
Time (min)
0
10
20
30
40
50
60
70
80
90
0.00
-20.93
μWatts
4-NH2-BA/1
0.00
-8.37
-16.74
-25.12
kJ mol-1 of injectant
Data: Tryp5x1030H_NDH
Model: OneSites
Chi^2/DoF = 3758
N 0.549 ±0.00779 Sites
K 1.21E5 ±9.38E3 M-1
ΔH -2.549E4 ±540.5 joules/mol
ΔS 11.9 joules/mol/deg
0.0
0.5
1.0
1.5
2.0
2.5
Molar Ratio

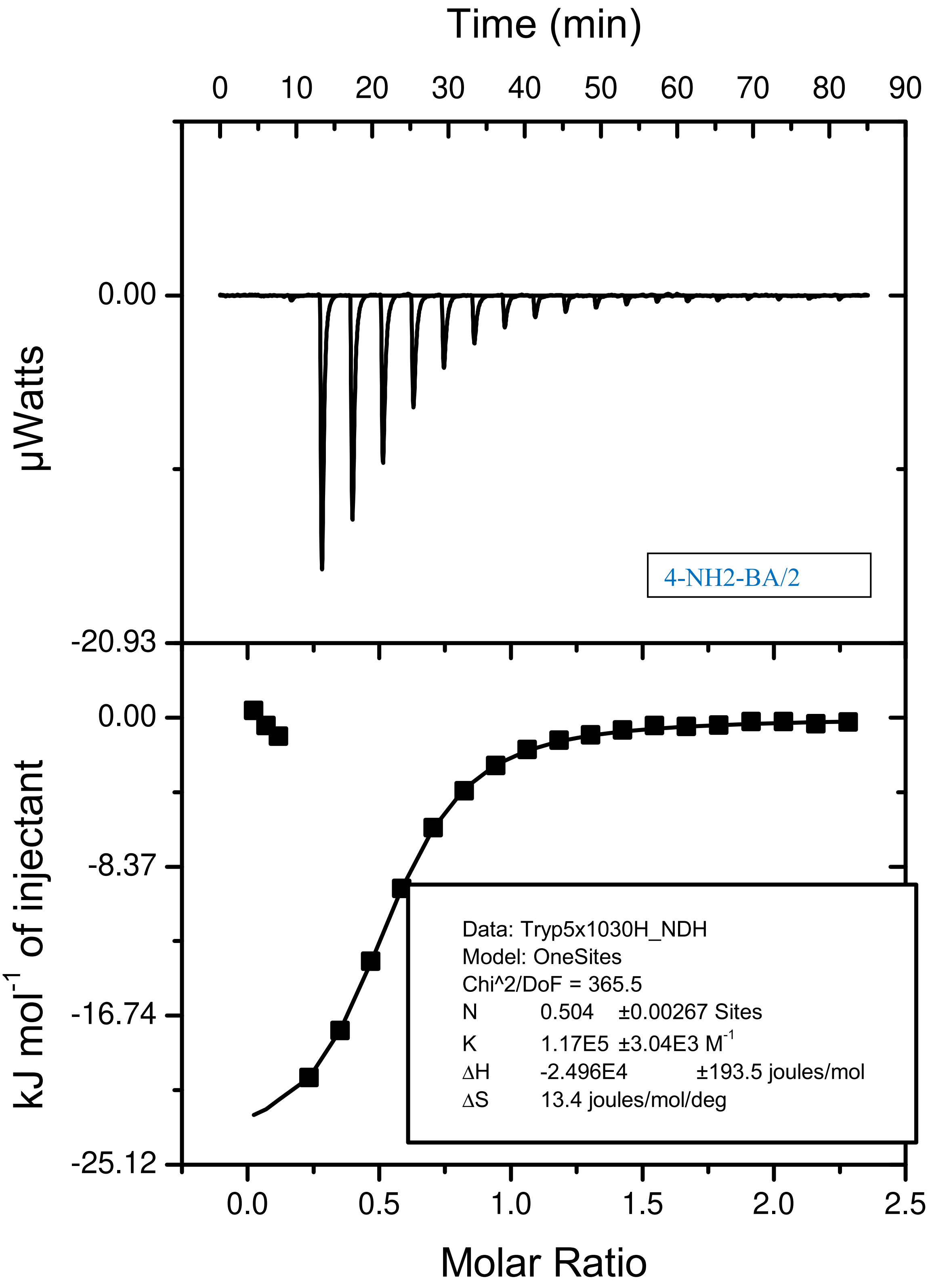

Time (min)
0
10
20
30
40
50
60
70
80
90
0.00
µWatts
-20.93
4-NH2-BA/2
0.00
-8.37
-16.74
-25.12
kJ mol-1 of injectant
0.0
0.5
1.0
1.5
2.0
2.5
Molar Ratio
Data: Tryp5x1030H_NDH
Model: OneSites
Chi^2/DoF = 365.5
N 0.504 ±0.00267 Sites
K 1.17E5 ±3.04E3 M-1
ΔH -2.496E4 ±193.5 joules/mol
ΔS 13.4 joules/mol/deg

3.3. ITC results for trypsin measurements in D_2O

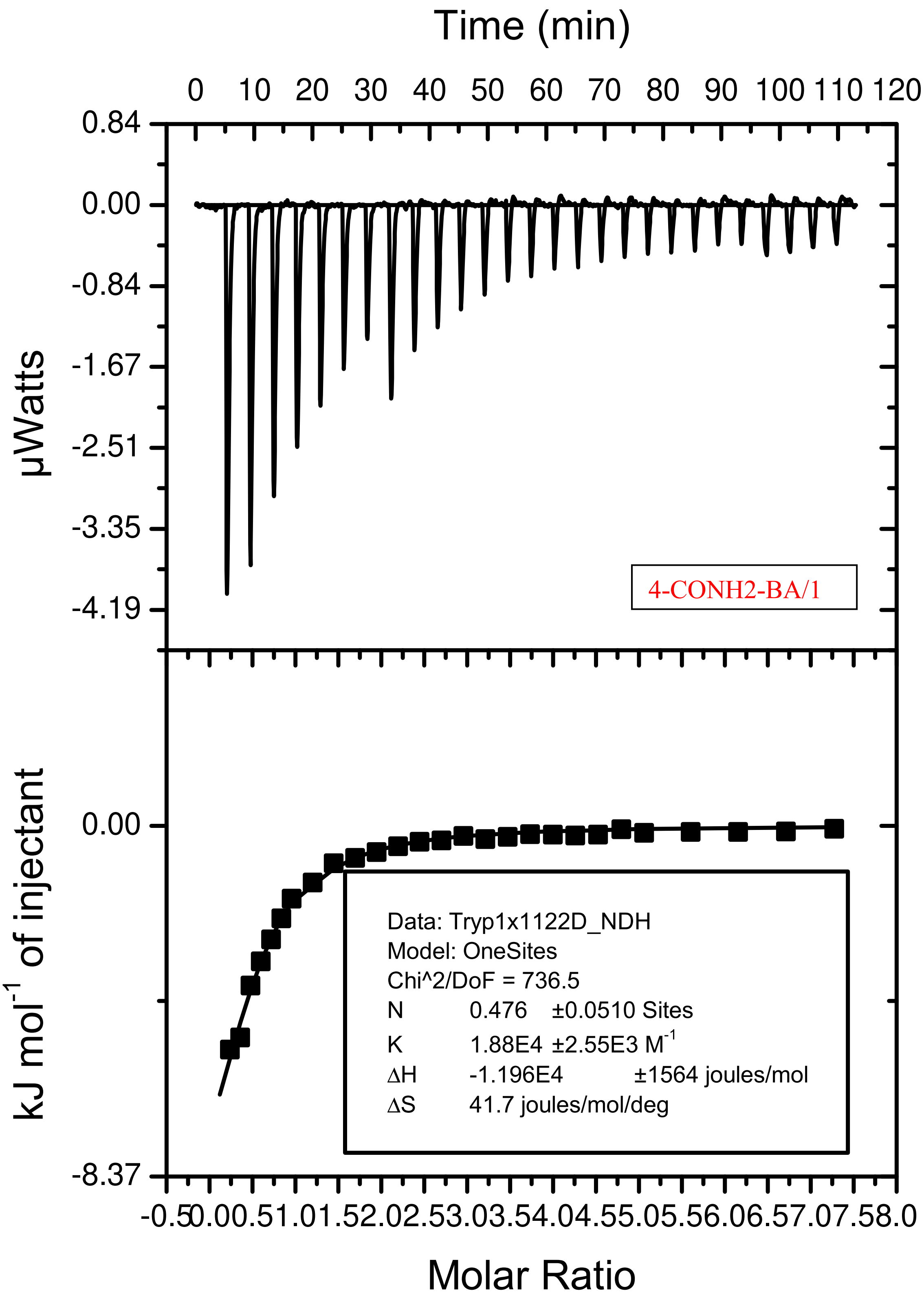

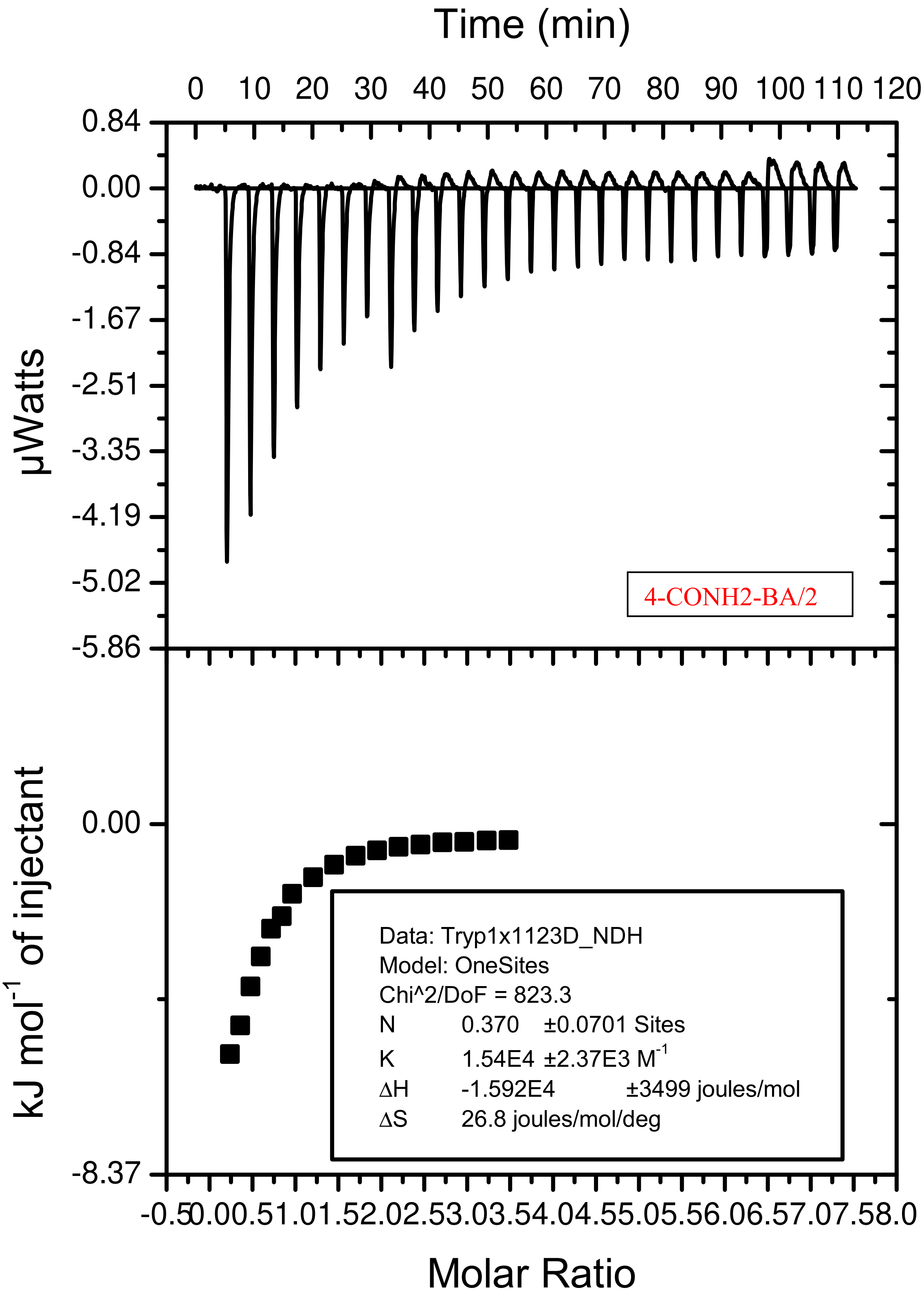

Time (min)
0 10 20 30 40 50 60 70 80 90 100 110 120
0.84
0.00
-0.84
-1.67
-2.51
-3.35
-4.19
-5.02
-5.86
µWatts
4-CONH2-BA/2
0.00
-8.37
kJ mol-1 of injectant
Data: Tryp1x1123D_NDH
Model: OneSites
Chi^2/DoF = 823.3
N 0.370 ±0.0701 Sites
K 1.54E4 ±2.37E3 M-1
ΔH -1.592E4 ±3499 joules/mol
ΔS 26.8 joules/mol/deg
-0.5 0.0 0.5 1.0 1.5 2.0 2.5 3.0 3.5 4.0 4.5 5.0 5.5 6.0 6.5 7.0 7.5 8.0
Molar Ratio

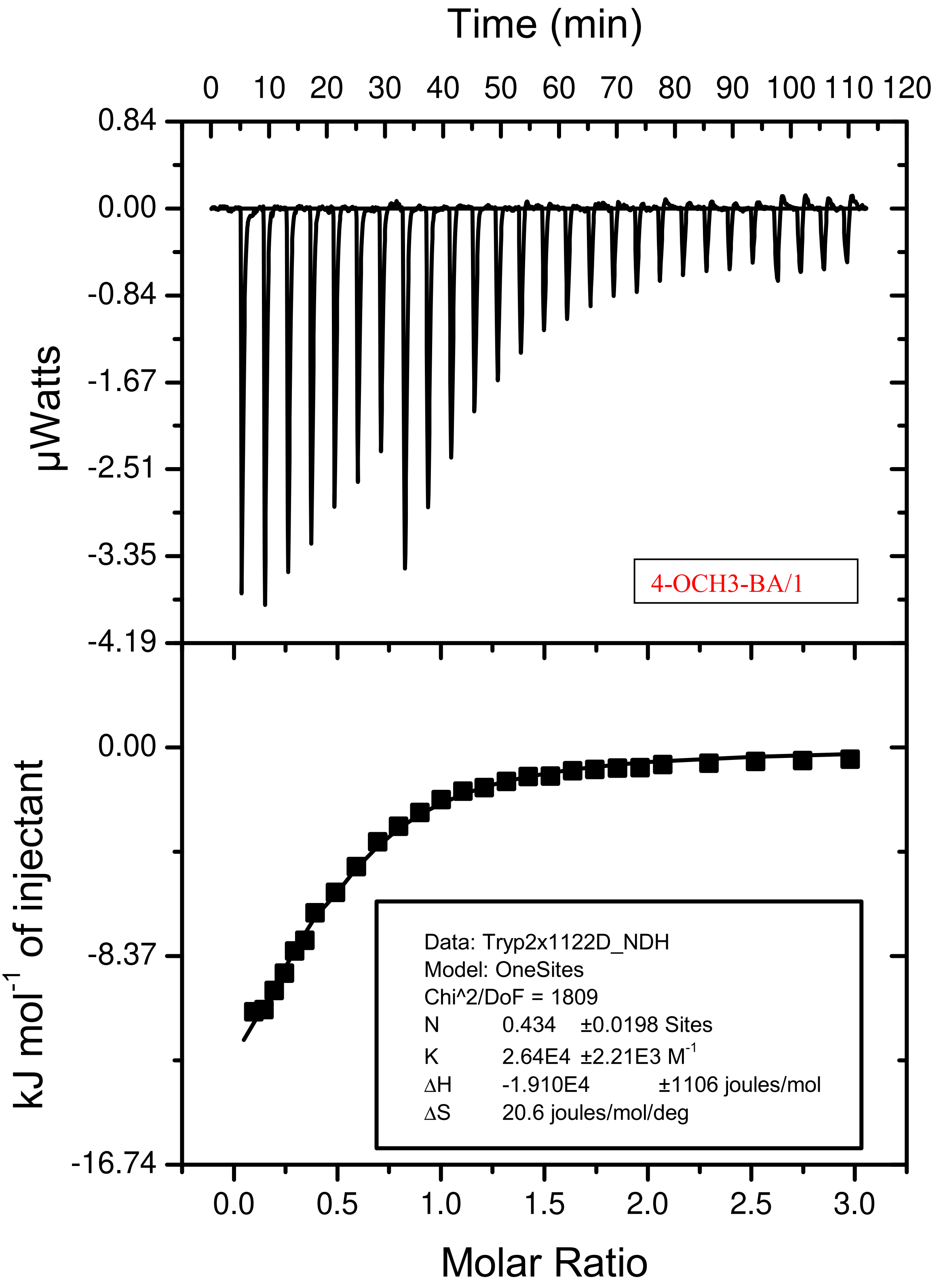
Time (min)
0 10 20 30 40 50 60 70 80 90 100 110 120
0.84
0.00
-0.84
-1.67
-2.51
-3.35
-4.19
μWatts
4-OCH3-BA/1
kJ mol-1 of injectant
0.00
-8.37
-16.74
Data: Tryp2x1122D_NDH
Model: OneSites
Chi^2/DoF = 1809
N 0.434 ±0.0198 Sites
K 2.64E4 ±2.21E3 M-1
ΔH -1.910E4 ±1106 joules/mol
ΔS 20.6 joules/mol/deg
0.0 0.5 1.0 1.5 2.0 2.5 3.0
Molar Ratio

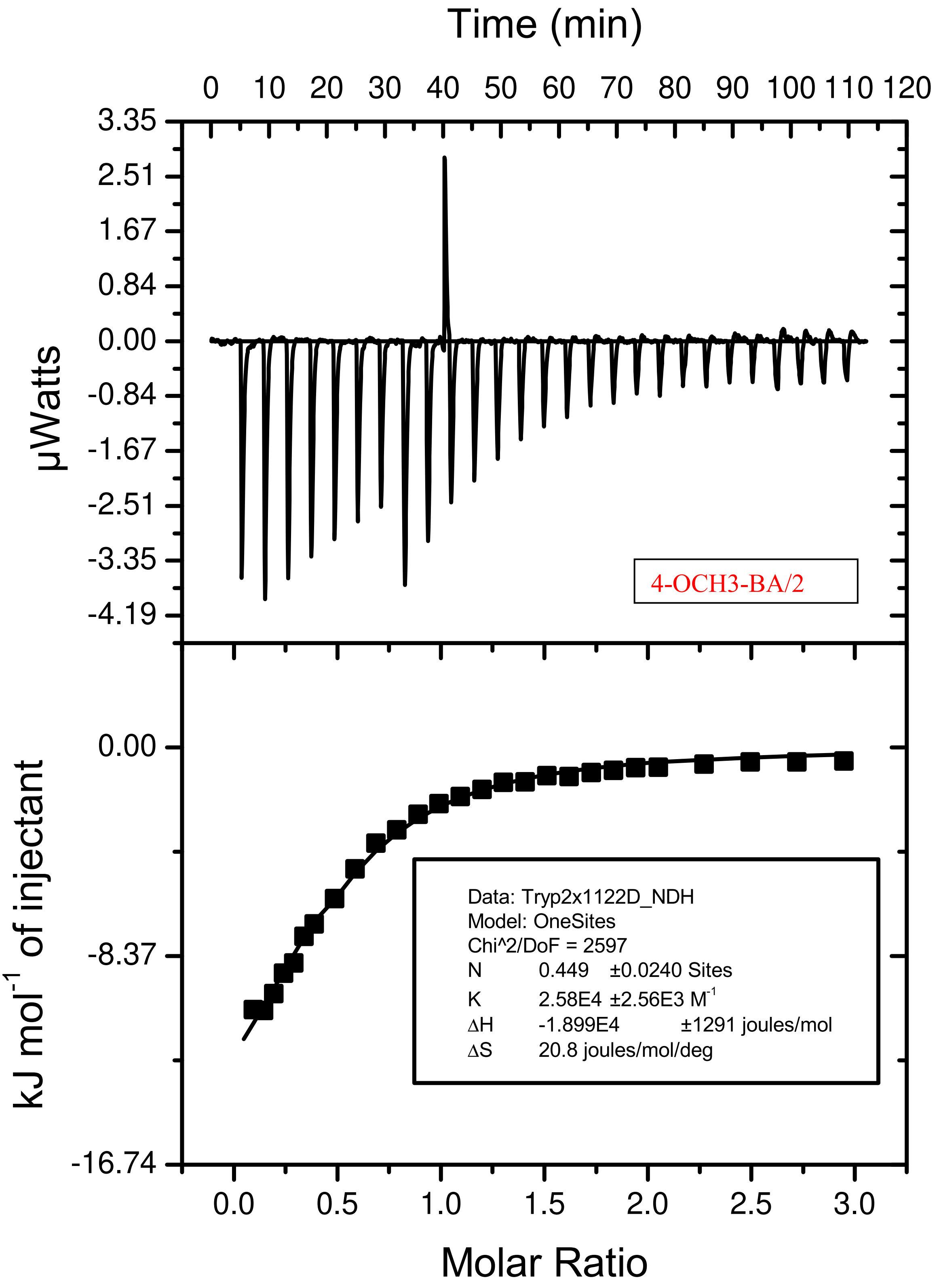
Time (min)
0 10 20 30 40 50 60 70 80 90 100 110 120
μWatts
3.35
2.51
1.67
0.84
0.00
-0.84
-1.67
-2.51
-3.35
-4.19
4-OCH3-BA/2
kJ mol-1 of injectant
0.00
-8.37
-16.74
Data: Tryp2x1122D_NDH
Model: OneSites
Chi^2/DoF = 2597
N 0.449 ±0.0240 Sites
K 2.58E4 ±2.56E3 M-1
ΔH -1.899E4 ±1291 joules/mol
ΔS 20.8 joules/mol/deg
0.0 0.5 1.0 1.5 2.0 2.5 3.0
Molar Ratio

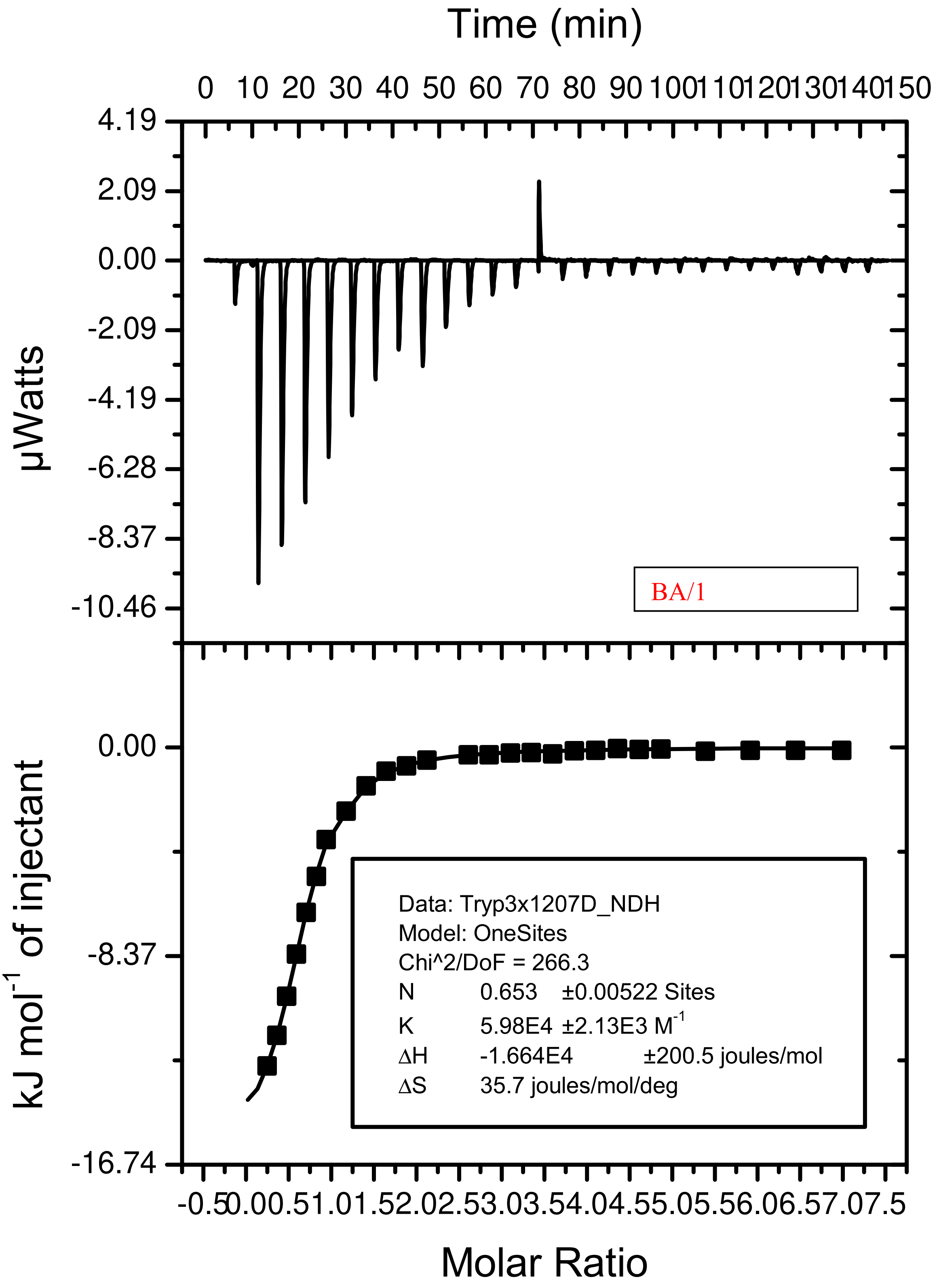
Time (min)
0 10 20 30 40 50 60 70 80 90 100 110 120 130 140 150
4.19
2.09
0.00
-2.09
-4.19
-6.28
-8.37
-10.46
μWatts
BA/1
0.00
-8.37
-16.74
kJ mol-1 of injectant
Data: Tryp3x1207D_NDH
Model: OneSites
Chi^2/DoF = 266.3
N 0.653 ±0.00522 Sites
K 5.98E4 ±2.13E3 M-1
ΔH -1.664E4 ±200.5 joules/mol
ΔS 35.7 joules/mol/deg
-0.5 0.0 0.5 1.0 1.5 2.0 2.5 3.0 3.5 4.0 4.5 5.0 5.5 6.0 6.5 7.0 7.5
Molar Ratio

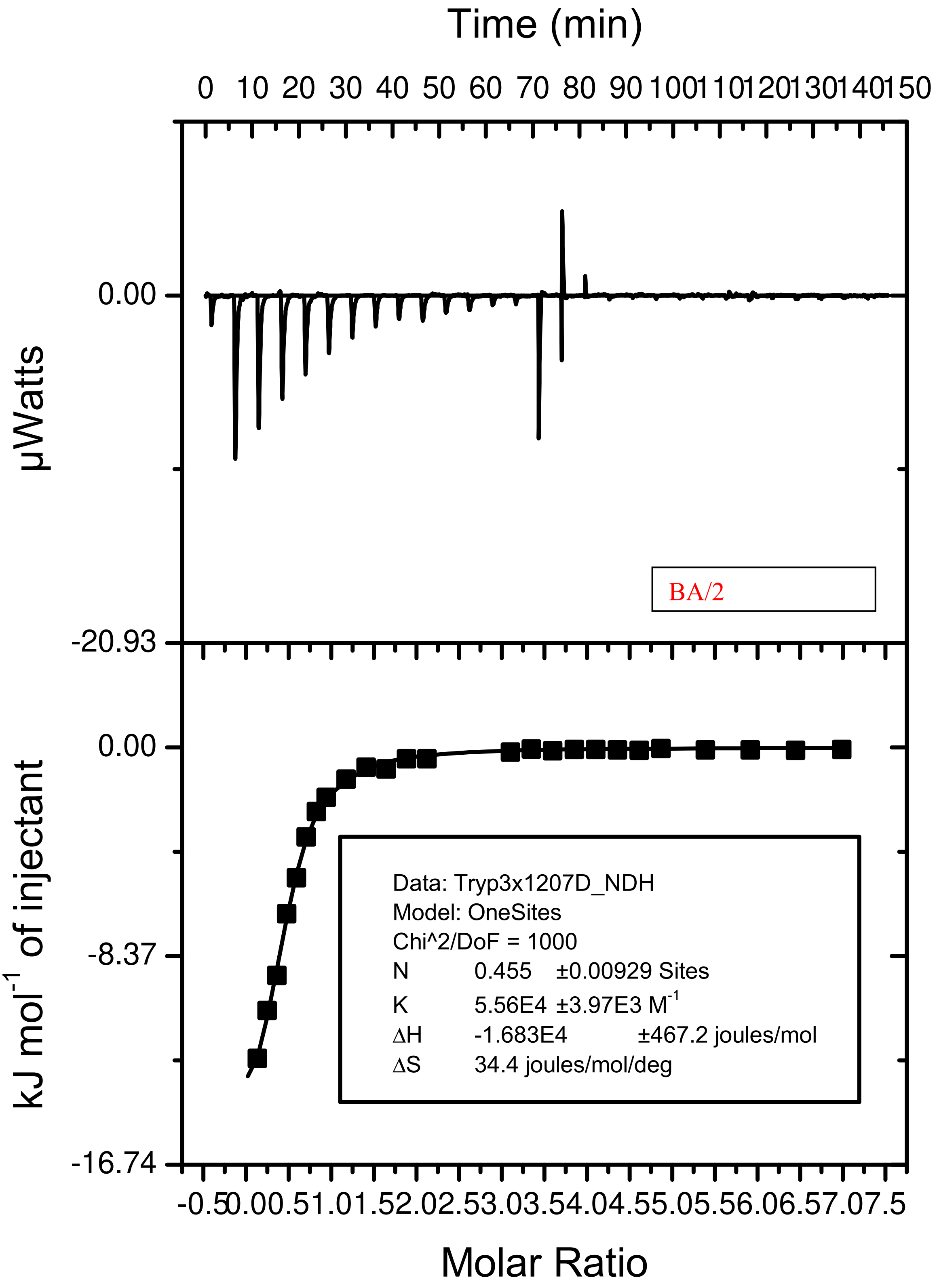
Time (min)
0 10 20 30 40 50 60 70 80 90 100 110 120 130 140 150
µWatts
0.00
-20.93
BA/2
kJ mol-1 of injectant
0.00
-8.37
-16.74
Data: Tryp3x1207D_NDH
Model: OneSites
Chi^2/DoF = 1000
N 0.455 ±0.00929 Sites
K 5.56E4 ±3.97E3 M-1
ΔH -1.683E4 ±467.2 joules/mol
ΔS 34.4 joules/mol/deg
-0.5 0.0 0.5 1.0 1.5 2.0 2.5 3.0 3.5 4.0 4.5 5.0 5.5 6.0 6.5 7.0 7.5
Molar Ratio

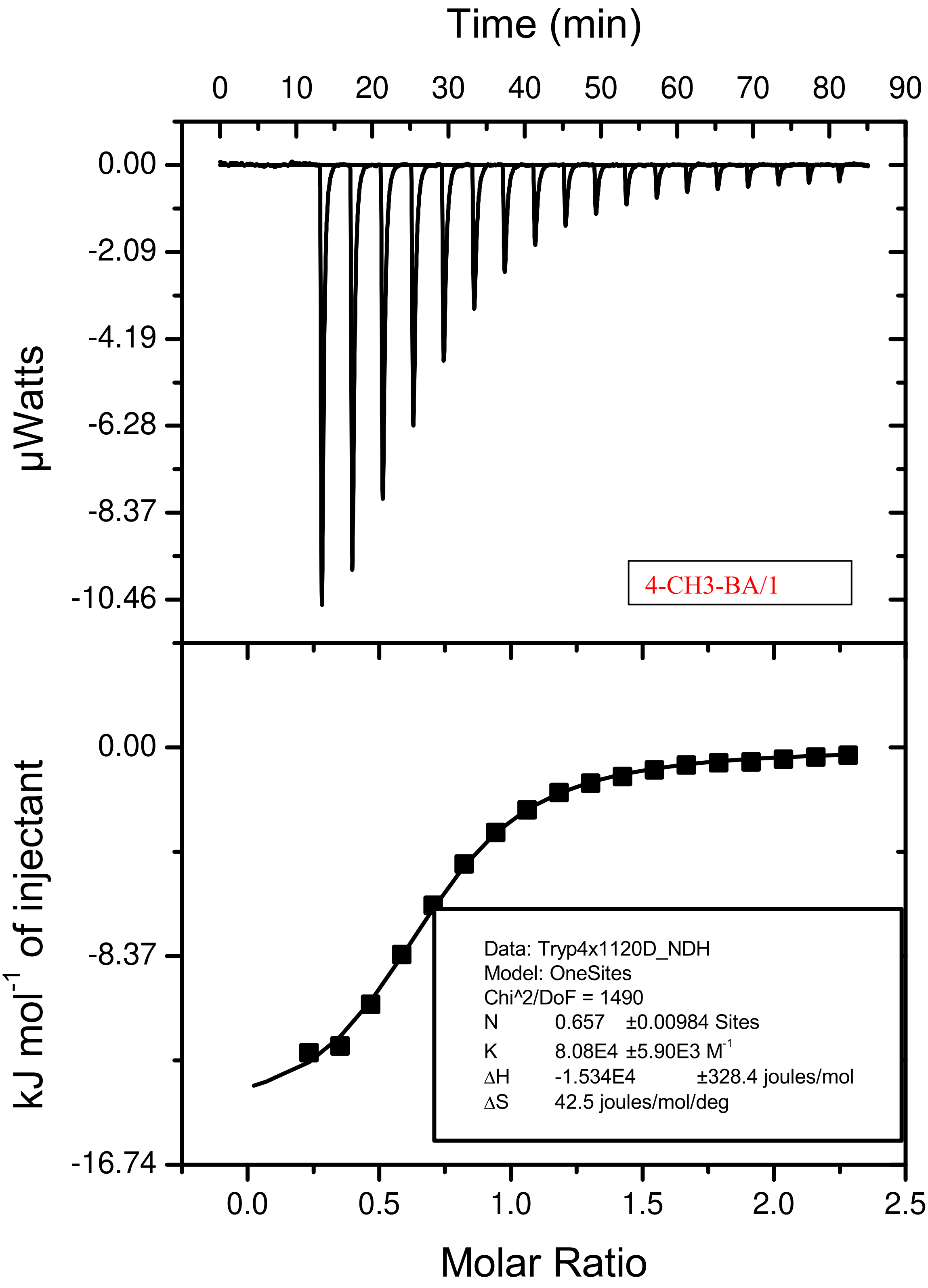

Time (min)
0 10 20 30 40 50 60 70 80 90
0.00
-2.09
-4.19
-6.28
-8.37
-10.46
μWatts
4-CH3-BA/1
0.00
-8.37
-16.74
kJ mol-1 of injectant
Data: Tryp4x1120D_NDH
Model: OneSites
Chi^2/DoF = 1490
N 0.657 ±0.00984 Sites
K 8.08E4 ±5.90E3 M-1
ΔH -1.534E4 ±328.4 joules/mol
ΔS 42.5 joules/mol/deg
0.0 0.5 1.0 1.5 2.0 2.5
Molar Ratio

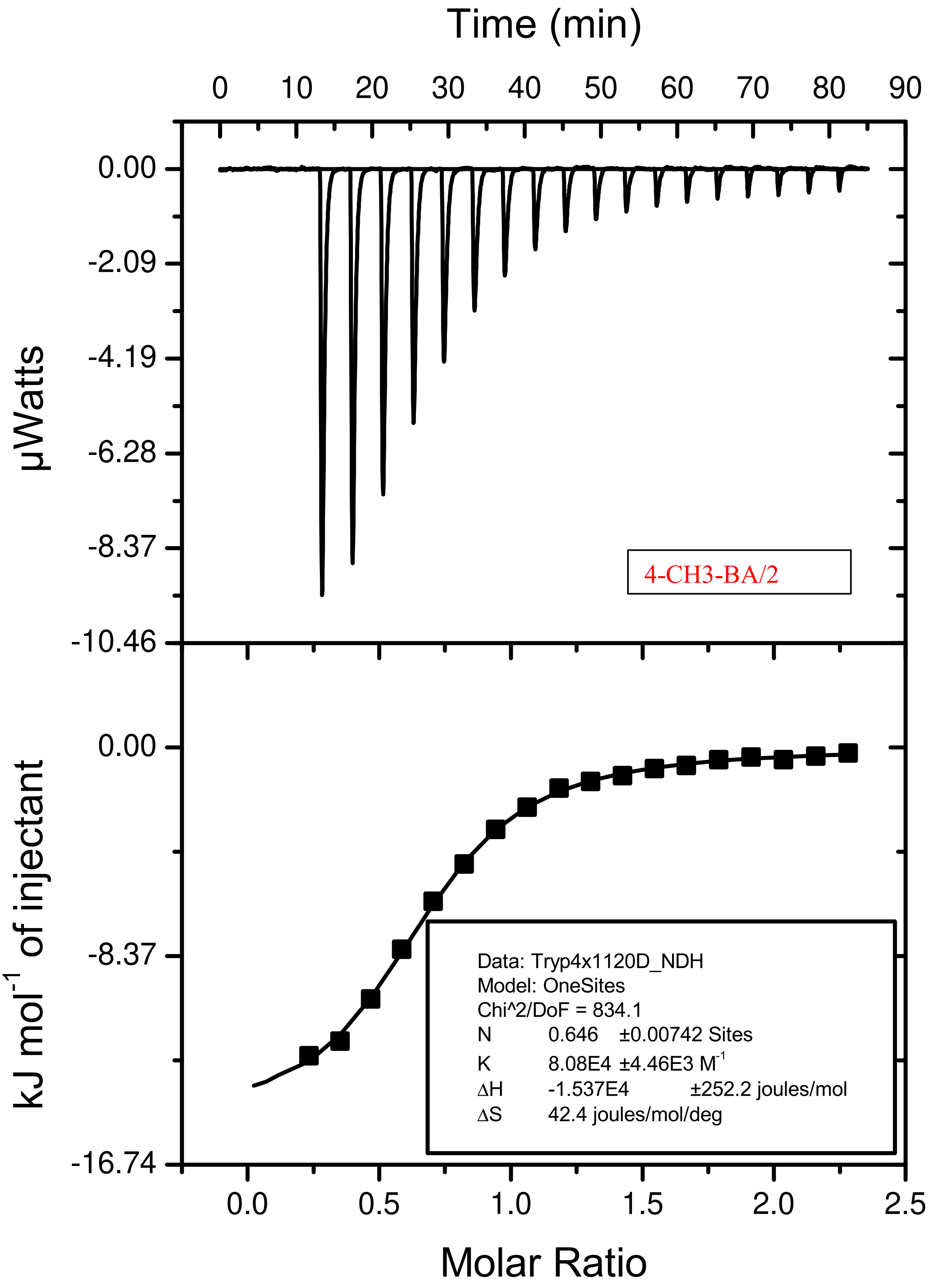
Time (min)
0
10
20
30
40
50
60
70
80
90
0.00
-2.09
-4.19
-6.28
-8.37
-10.46
μWatts
4-CH3-BA/2
0.00
-8.37
-16.74
kJ mol-1 of injectant
Data: Tryp4x1120D_NDH
Model: OneSites
Chi^2/DoF = 834.1
N 0.646 ±0.00742 Sites
K 8.08E4 ±4.46E3 M-1
ΔH -1.537E4 ±252.2 joules/mol
ΔS 42.4 joules/mol/deg
0.0
0.5
1.0
1.5
2.0
2.5
Molar Ratio

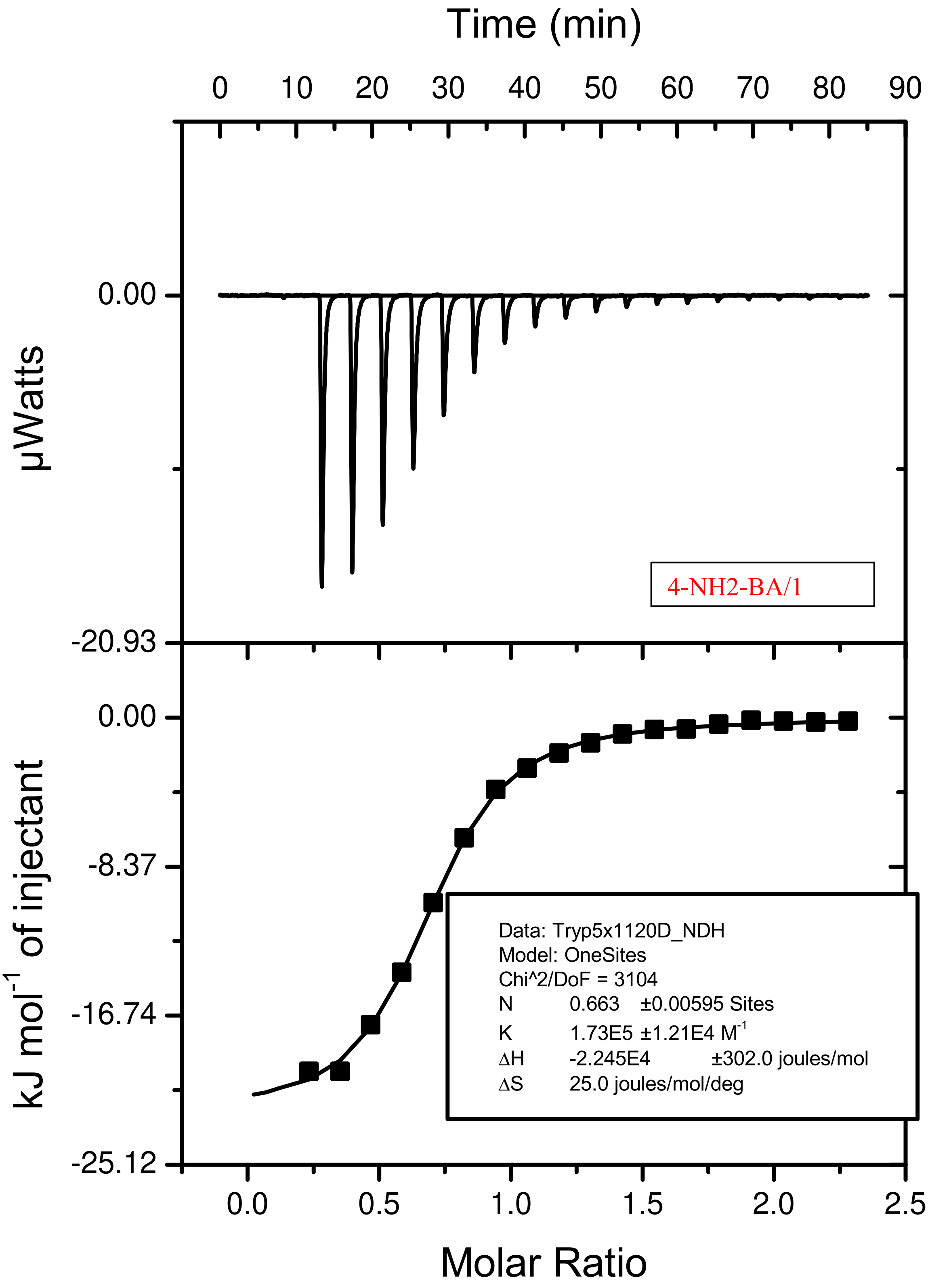

Time (min)
0 10 20 30 40 50 60 70 80 90
µWatts
0.00
-20.93
4-NH2-BA/1
kJ mol-1 of injectant
0.00
-8.37
-16.74
-25.12
Data: Tryp5x1120D_NDH
Model: OneSites
Chi^2/DoF = 3104
N 0.663 ±0.00595 Sites
K 1.73E5 ±1.21E4 M-1
ΔH -2.245E4 ±302.0 joules/mol
ΔS 25.0 joules/mol/deg
0.0 0.5 1.0 1.5 2.0 2.5
Molar Ratio

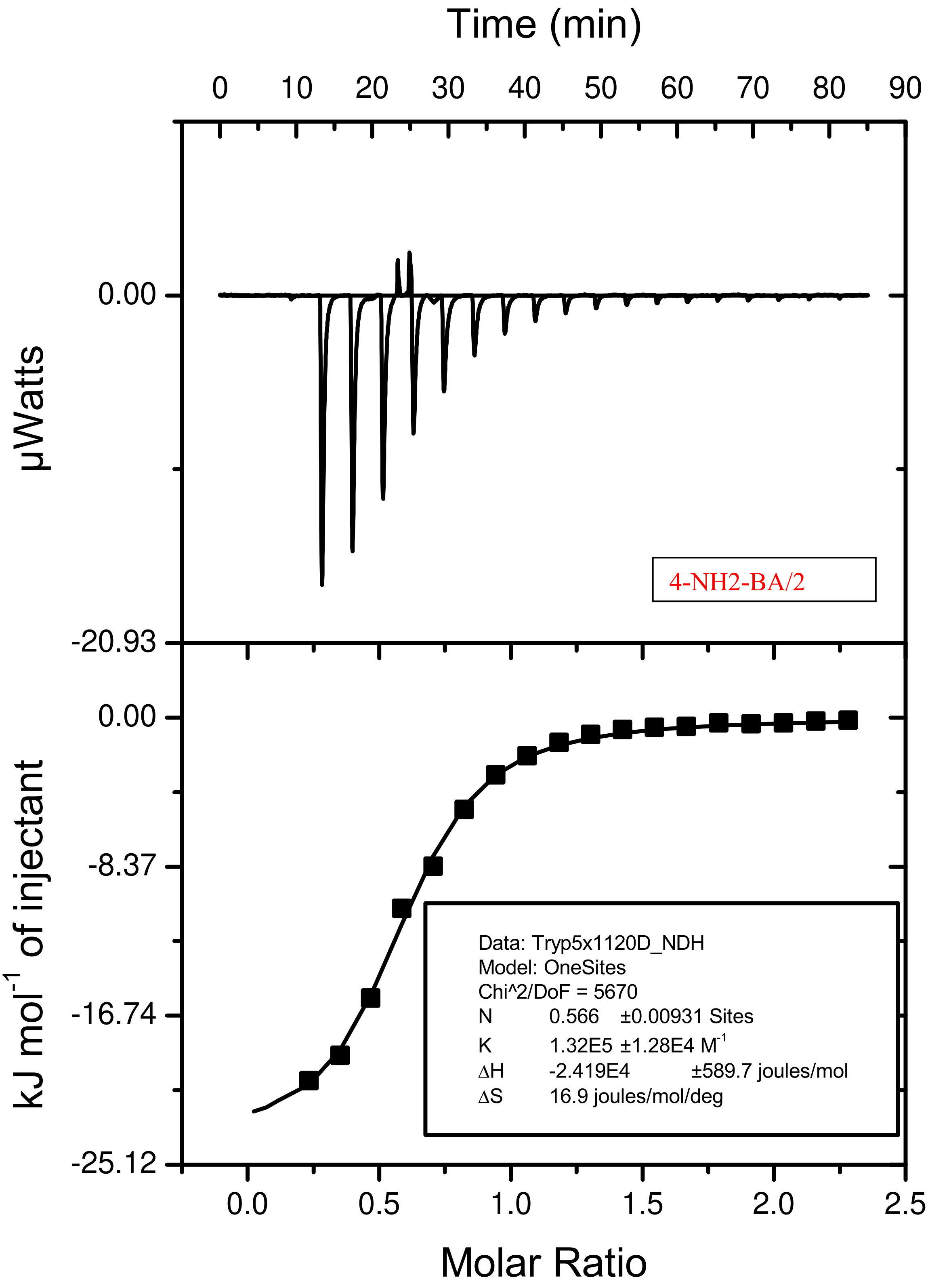
Time (min)
0 10 20 30 40 50 60 70 80 90
μWatts
0.00
-20.93
4-NH2-BA/2
kJ mol-1 of injectant
0.00
-8.37
-16.74
-25.12
Data: Tryp5x1120D_NDH
Model: OneSites
Chi^2/DoF = 5670
N 0.566 ±0.00931 Sites
K 1.32E5 ±1.28E4 M-1
ΔH -2.419E4 ±589.7 joules/mol
ΔS 16.9 joules/mol/deg
0.0 0.5 1.0 1.5 2.0 2.5
Molar Ratio

3.4. ITC results for CAII measurements in H_2O

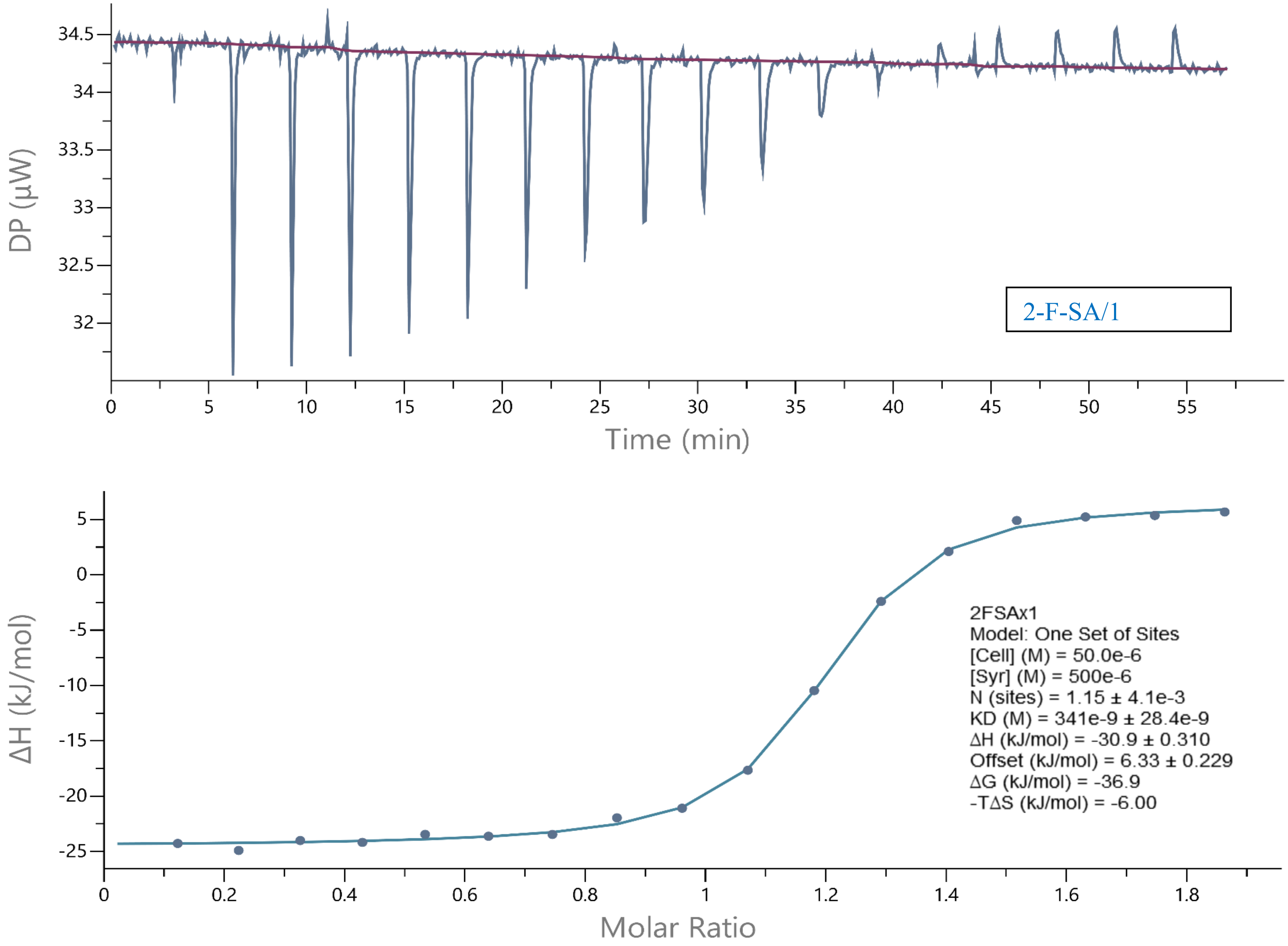

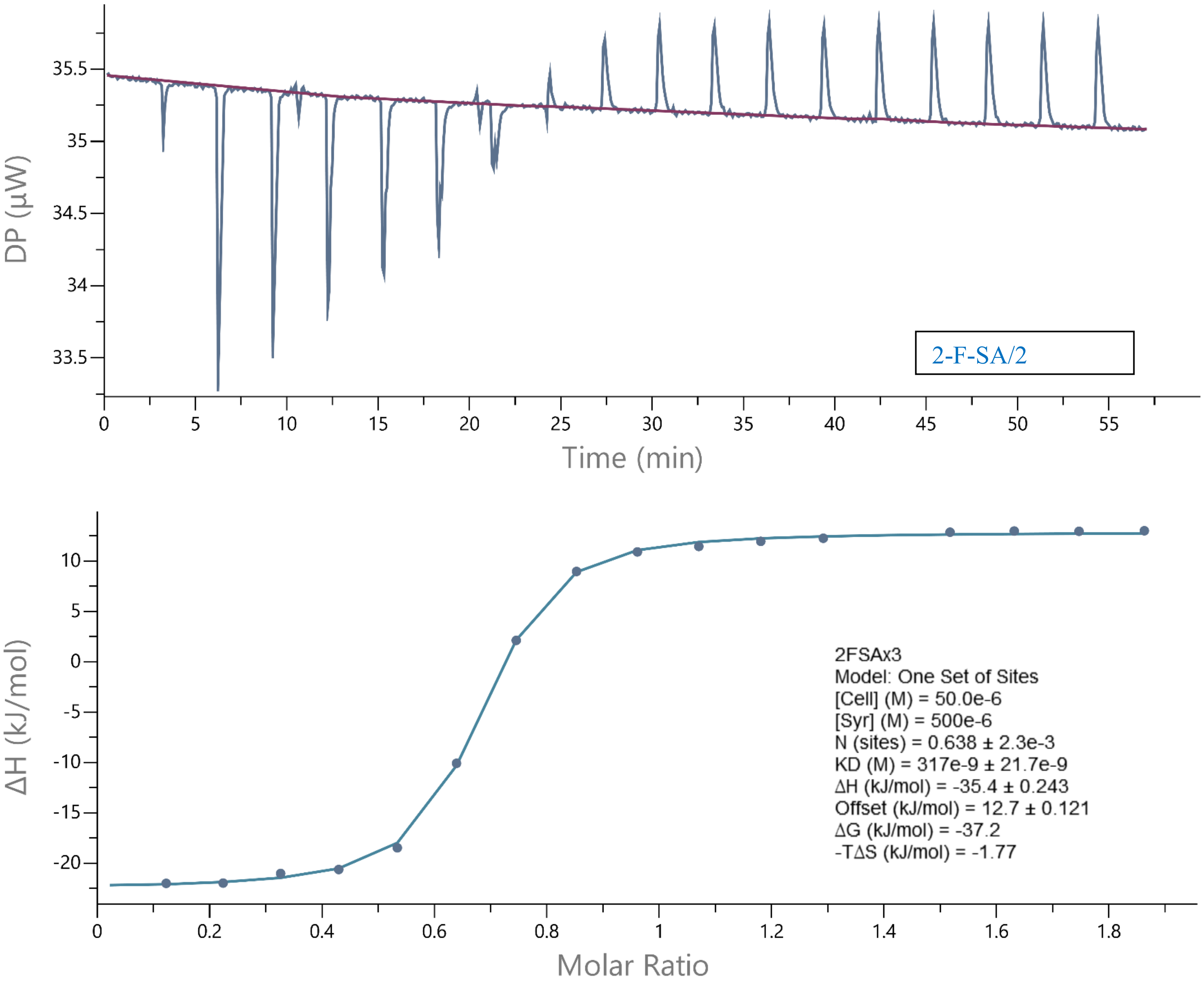

DP (µW)
35.5
35
34.5
34
33.5
0
5
10
15
20
25
30
35
40
45
50
55
Time (min)
2-F-SA/2
ΔH (kJ/mol)
10
5
0
-5
-10
-15
-20
0
0.2
0.4
0.6
0.8
1
1.2
1.4
1.6
1.8
Molar Ratio
2FSAx3
Model: One Set of Sites
[Cell] (M) = 50.0e-6
[Syr] (M) = 500e-6
N (sites) = 0.638 ± 2.3e-3
KD (M) = 317e-9 ± 21.7e-9
ΔH (kJ/mol) = -35.4 ± 0.243
Offset (kJ/mol) = 12.7 ± 0.121
ΔG (kJ/mol) = -37.2
-TΔS (kJ/mol) = -1.77

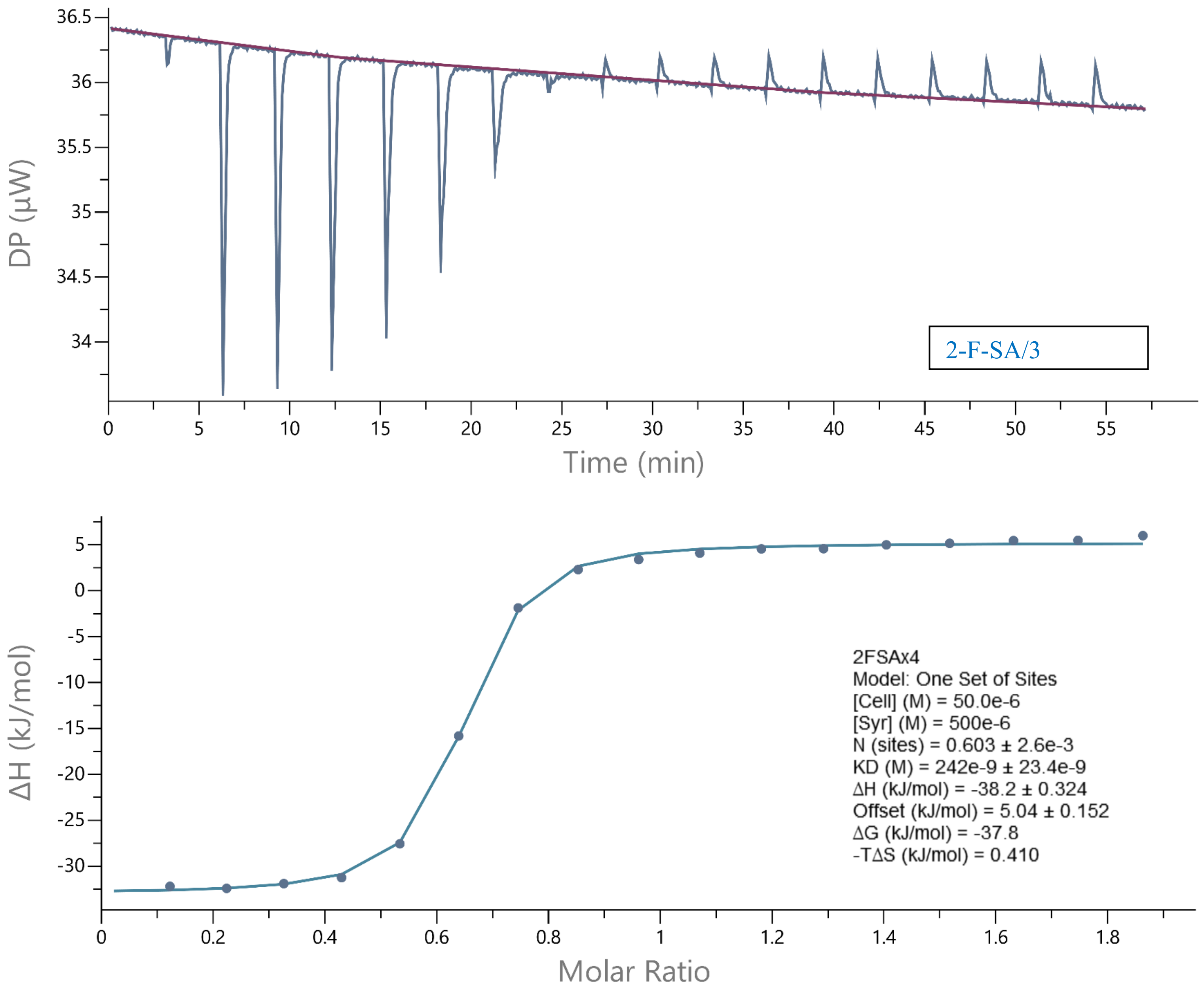
2-F-SA/3
DP (μW)
Time (min)
ΔH (kJ/mol)
Molar Ratio
2FSAx4
Model: One Set of Sites
[Cell] (M) = 50.0e-6
[Syr] (M) = 500e-6
N (sites) = 0.603 ± 2.6e-3
KD (M) = 242e-9 ± 23.4e-9
ΔH (kJ/mol) = -38.2 ± 0.324
Offset (kJ/mol) = 5.04 ± 0.152
ΔG (kJ/mol) = -37.8
-TΔS (kJ/mol) = 0.410

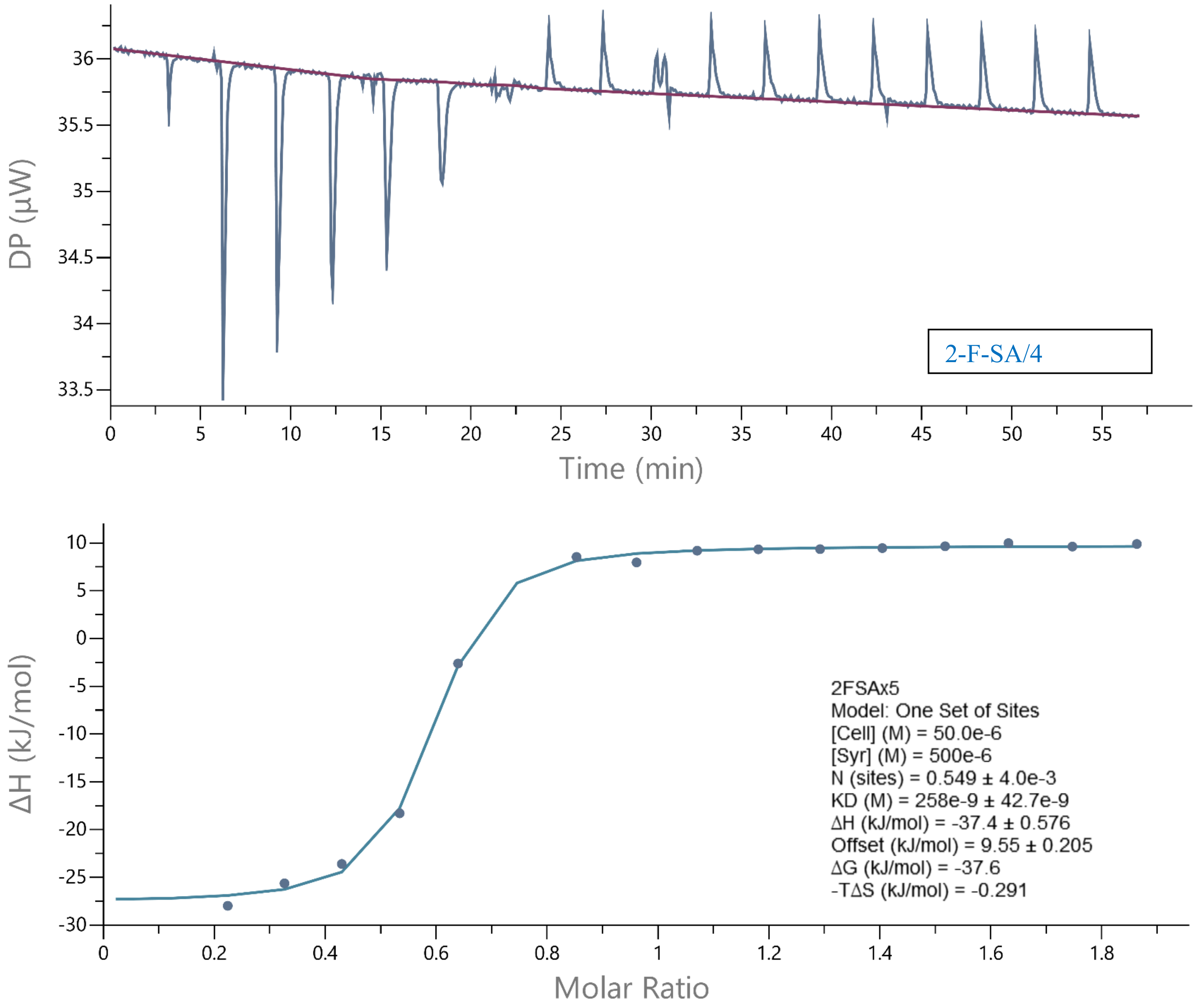
DP (μW)
36
35.5
35
34.5
34
33.5
0 5 10 15 20 25 30 35 40 45 50 55
Time (min)
2-F-SA/4
ΔH (kJ/mol)
10
5
0
-5
-10
-15
-20
-25
-30
0 0.2 0.4 0.6 0.8 1 1.2 1.4 1.6 1.8
Molar Ratio
2FSAx5
Model: One Set of Sites
[Cell] (M) = 50.0e-6
[Syr] (M) = 500e-6
N (sites) = 0.549 ± 4.0e-3
KD (M) = 258e-9 ± 42.7e-9
ΔH (kJ/mol) = -37.4 ± 0.576
Offset (kJ/mol) = 9.55 ± 0.205
ΔG (kJ/mol) = -37.6
-TΔS (kJ/mol) = -0.291

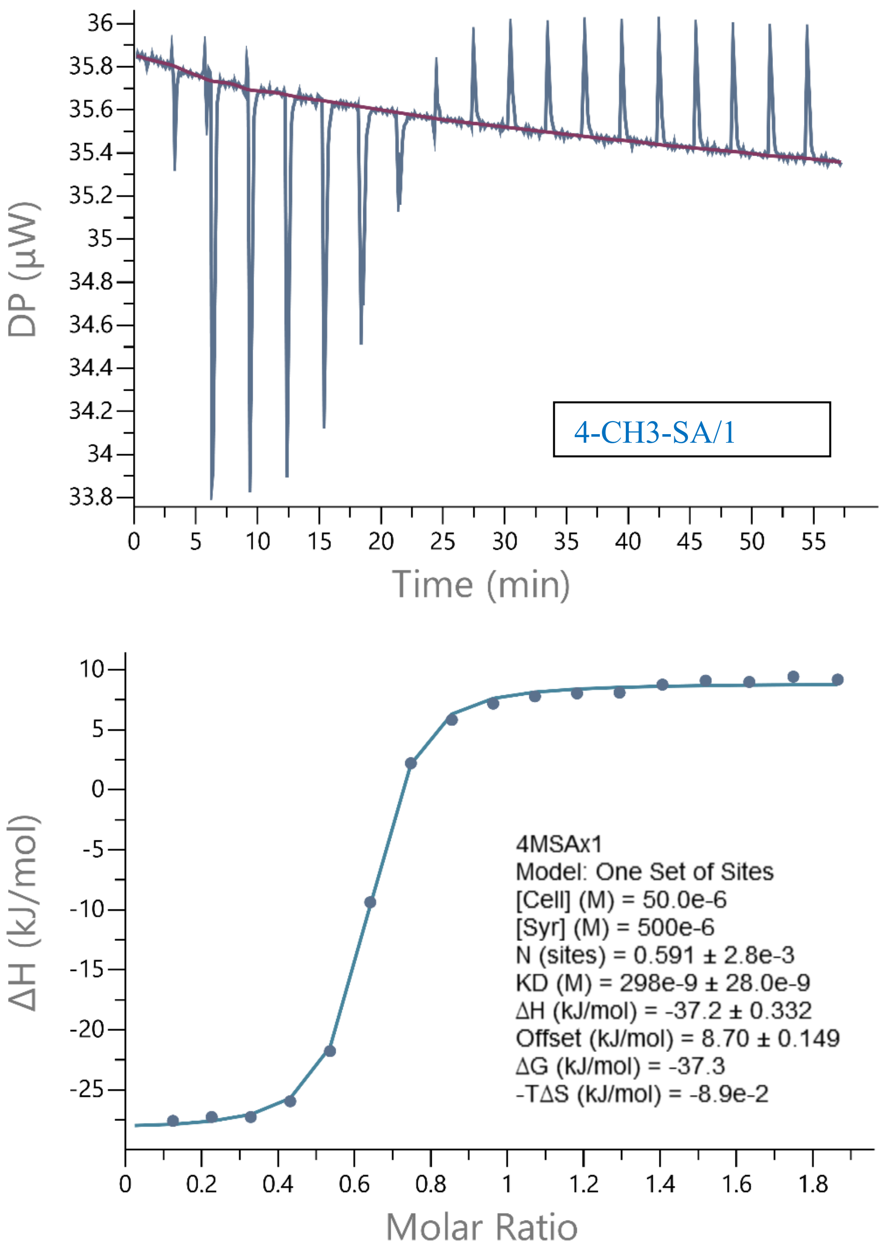
36
35.8
35.6
35.4
35.2
35
34.8
34.6
34.4
34.2
34
33.8
DP (μW)
0 5 10 15 20 25 30 35 40 45 50 55
Time (min)
4-CH3-SA/1
10
5
0
-5
-10
-15
-20
-25
ΔH (kJ/mol)
0 0.2 0.4 0.6 0.8 1 1.2 1.4 1.6 1.8
Molar Ratio
4MSAx1
Model: One Set of Sites
[Cell] (M) = 50.0e-6
[Syr] (M) = 500e-6
N (sites) = 0.591 ± 2.8e-3
KD (M) = 298e-9 ± 28.0e-9
ΔH (kJ/mol) = -37.2 ± 0.332
Offset (kJ/mol) = 8.70 ± 0.149
ΔG (kJ/mol) = -37.3
-TΔS (kJ/mol) = -8.9e-2

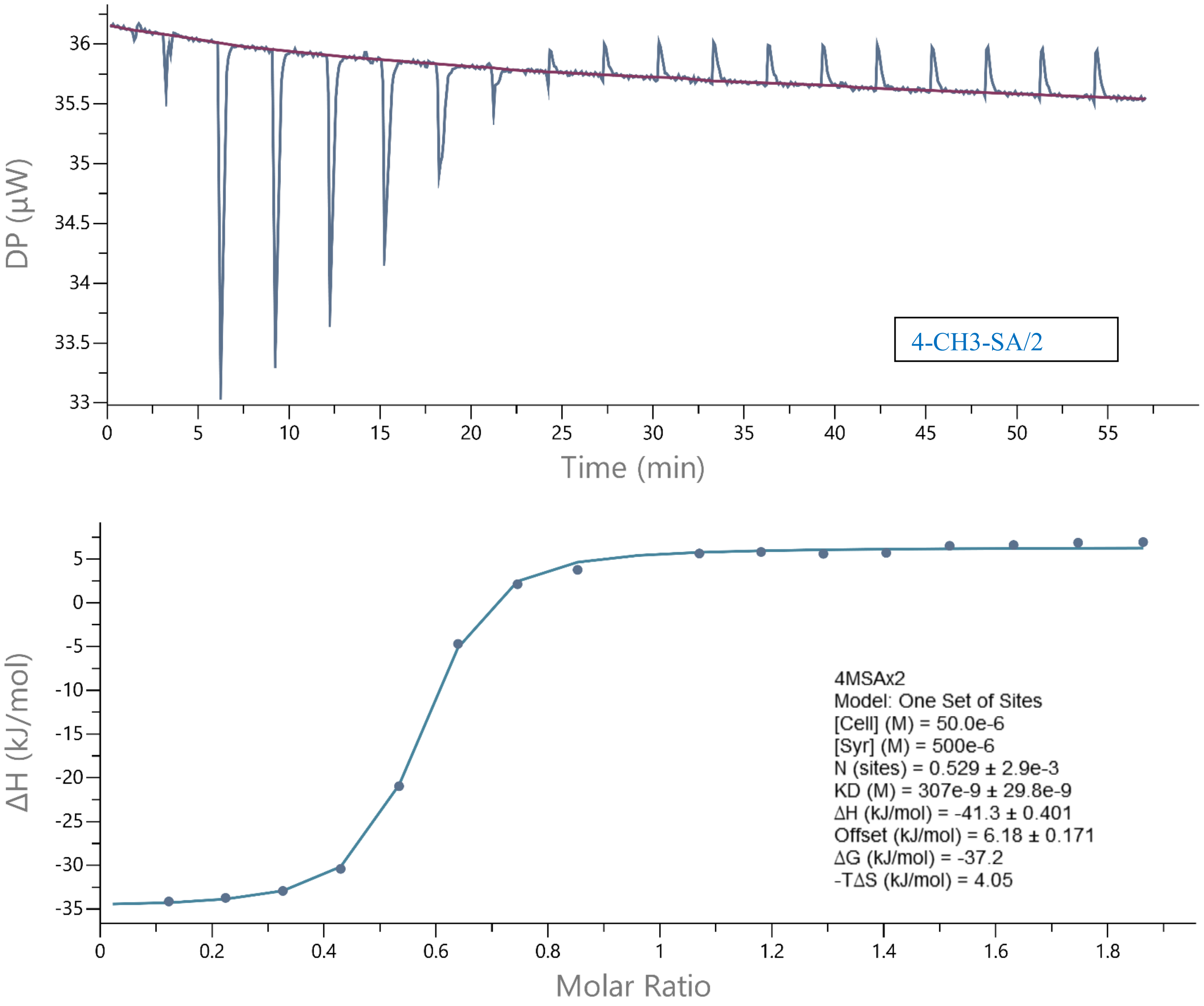

DP (μW)
36
35.5
35
34.5
34
33.5
33
0
5
10
15
20
25
30
35
40
45
50
55
Time (min)
4-CH3-SA/2
ΔH (kJ/mol)
5
0
-5
-10
-15
-20
-25
-30
-35
0
0.2
0.4
0.6
0.8
1
1.2
1.4
1.6
1.8
Molar Ratio
4MSAx2
Model: One Set of Sites
[Cell] (M) = 50.0e-6
[Syr] (M) = 500e-6
N (sites) = 0.529 ± 2.9e-3
KD (M) = 307e-9 ± 29.8e-9
ΔH (kJ/mol) = -41.3 ± 0.401
Offset (kJ/mol) = 6.18 ± 0.171
ΔG (kJ/mol) = -37.2
-TΔS (kJ/mol) = 4.05

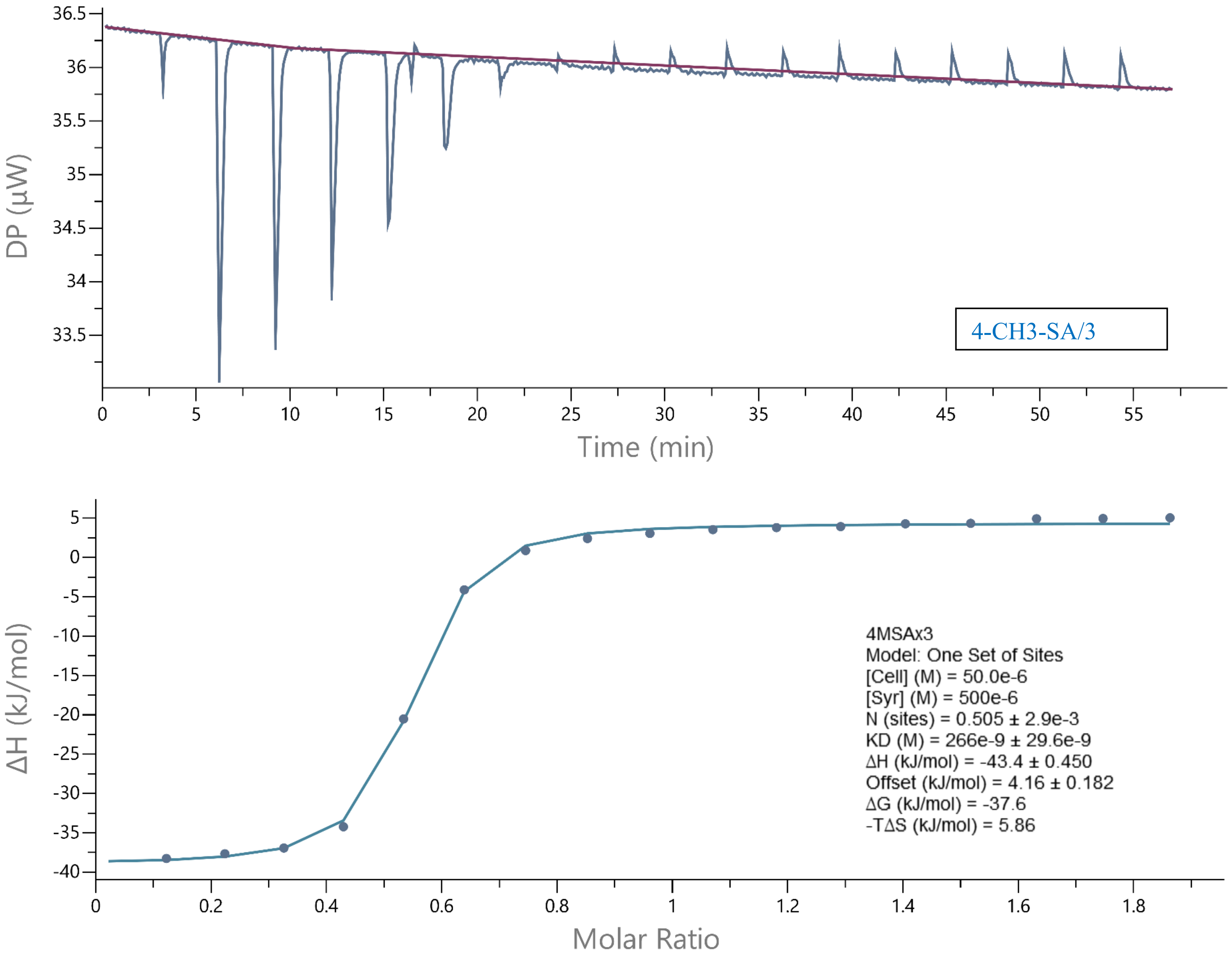

36.5
36
35.5
35
34.5
34
33.5
DP (µW)
0
5
10
15
20
25
30
35
40
45
50
55
Time (min)
4-CH3-SA/3
5
0
-5
-10
-15
-20
-25
-30
-35
-40
ΔH (kJ/mol)
0
0.2
0.4
0.6
0.8
1
1.2
1.4
1.6
1.8
Molar Ratio
4MSAx3
Model: One Set of Sites
[Cell] (M) = 50.0e-6
[Syr] (M) = 500e-6
N (sites) = 0.505 ± 2.9e-3
KD (M) = 266e-9 ± 29.6e-9
ΔH (kJ/mol) = -43.4 ± 0.450
Offset (kJ/mol) = 4.16 ± 0.182
ΔG (kJ/mol) = -37.6
-TΔS (kJ/mol) = 5.86

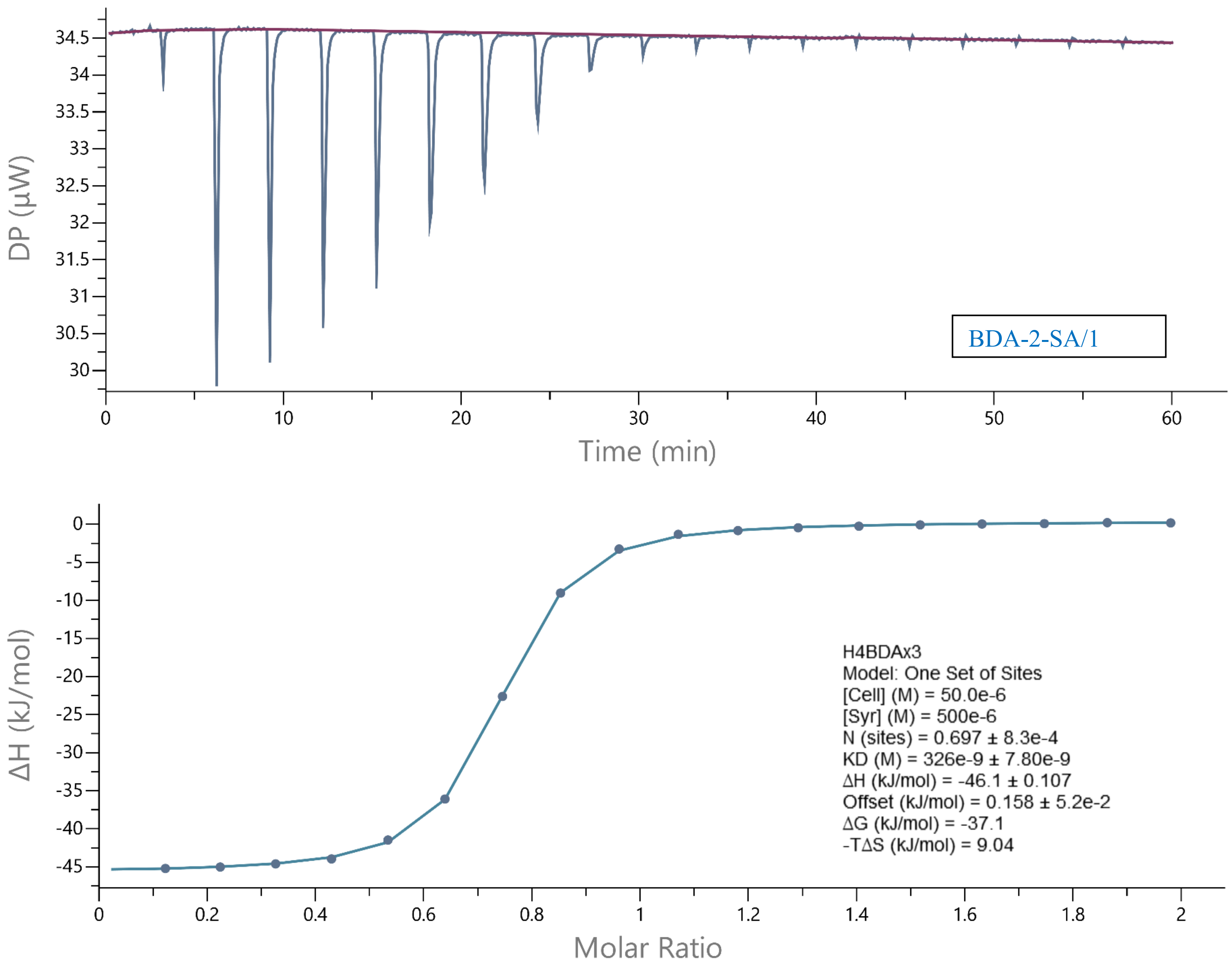

DP (μW)
34.5
34
33.5
33
32.5
32
31.5
31
30.5
30
0
10
20
30
40
50
60
Time (min)
BDA-2-SA/1
ΔH (kJ/mol)
0
-5
-10
-15
-20
-25
-30
-35
-40
-45
0
0.2
0.4
0.6
0.8
1
1.2
1.4
1.6
1.8
2
Molar Ratio
H4BDAx3
Model: One Set of Sites
[Cell] (M) = 50.0e-6
[Syr] (M) = 500e-6
N (sites) = 0.697 ± 8.3e-4
KD (M) = 326e-9 ± 7.80e-9
ΔH (kJ/mol) = -46.1 ± 0.107
Offset (kJ/mol) = 0.158 ± 5.2e-2
ΔG (kJ/mol) = -37.1
-TΔS (kJ/mol) = 9.04

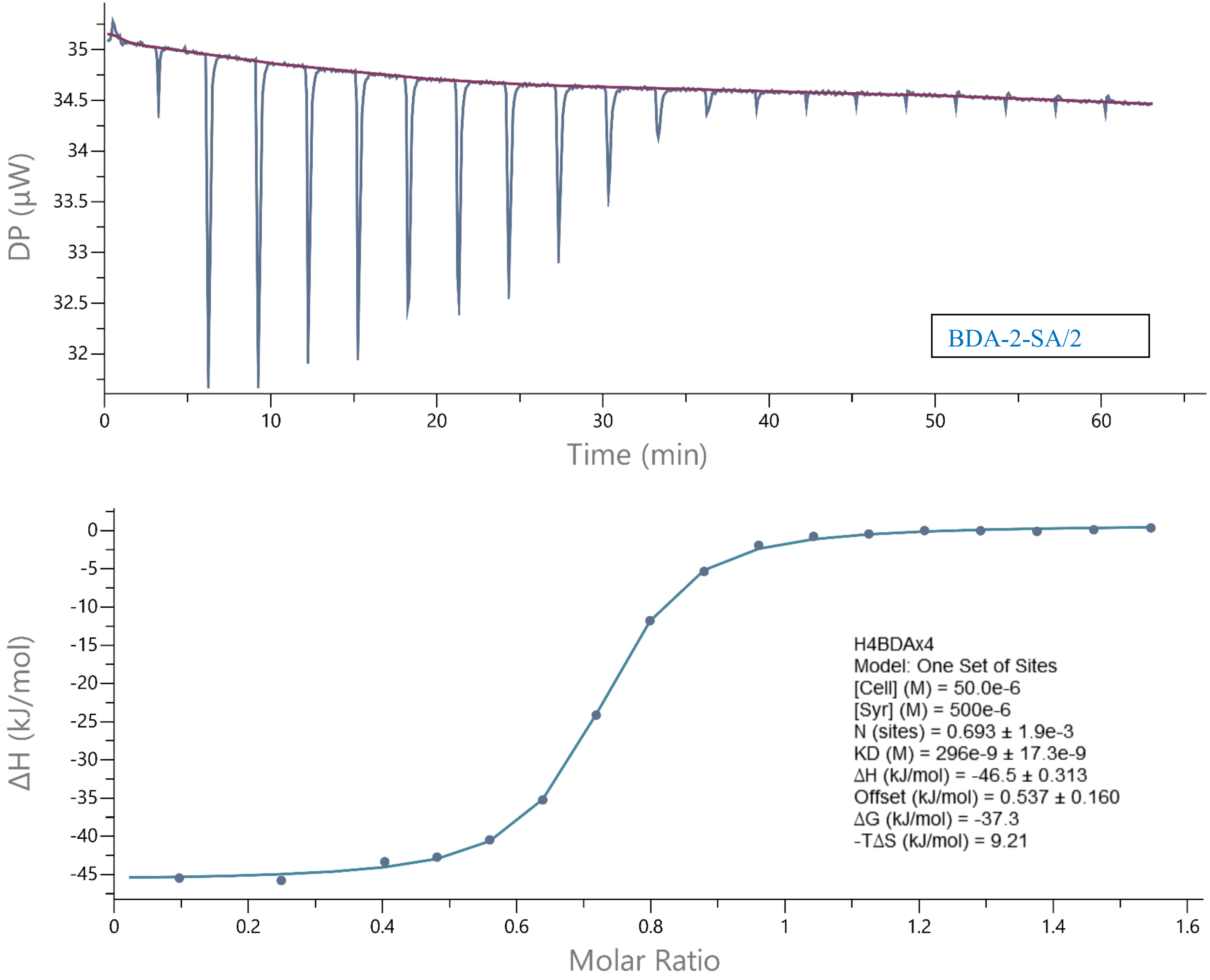
BDA-2-SA/2
DP (μW)
Time (min)
H4BDAx4
Model: One Set of Sites
[Cell] (M) = 50.0e-6
[Syr] (M) = 500e-6
N (sites) = 0.693 ± 1.9e-3
KD (M) = 296e-9 ± 17.3e-9
ΔH (kJ/mol) = -46.5 ± 0.313
Offset (kJ/mol) = 0.537 ± 0.160
ΔG (kJ/mol) = -37.3
-TΔS (kJ/mol) = 9.21
ΔH (kJ/mol)
Molar Ratio

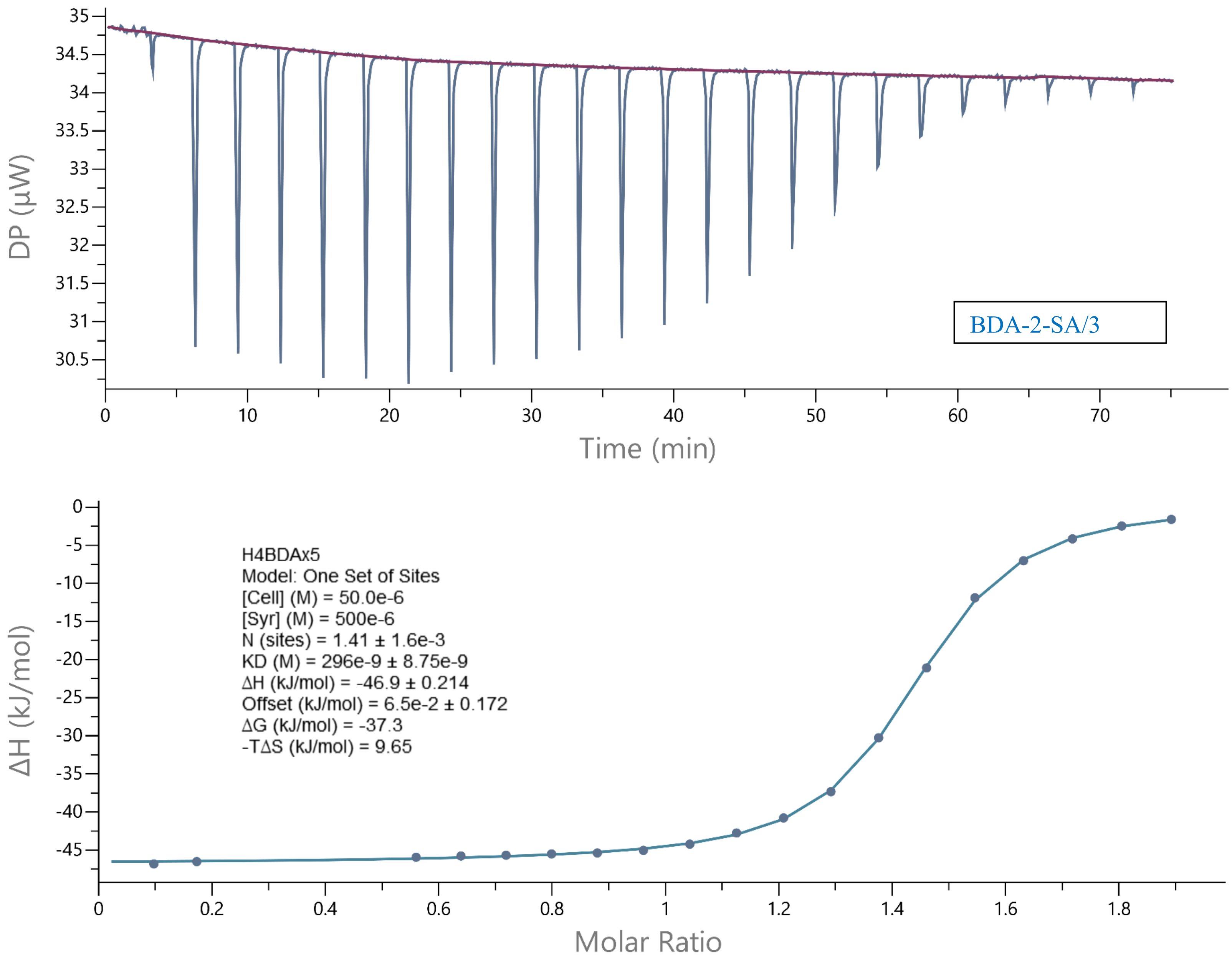
35
34.5
34
33.5
33
32.5
32
31.5
31
30.5
DP (µW)
0
10
20
30
40
50
60
70
Time (min)
BDA-2-SA/3
0
-5
-10
-15
-20
-25
-30
-35
-40
-45
ΔH (kJ/mol)
H4BDAx5
Model: One Set of Sites
[Cell] (M) = 50.0e-6
[Syr] (M) = 500e-6
N (sites) = 1.41 ± 1.6e-3
KD (M) = 296e-9 ± 8.75e-9
ΔH (kJ/mol) = -46.9 ± 0.214
Offset (kJ/mol) = 6.5e-2 ± 0.172
ΔG (kJ/mol) = -37.3
-TΔS (kJ/mol) = 9.65
0
0.2
0.4
0.6
0.8
1
1.2
1.4
1.6
1.8
Molar Ratio

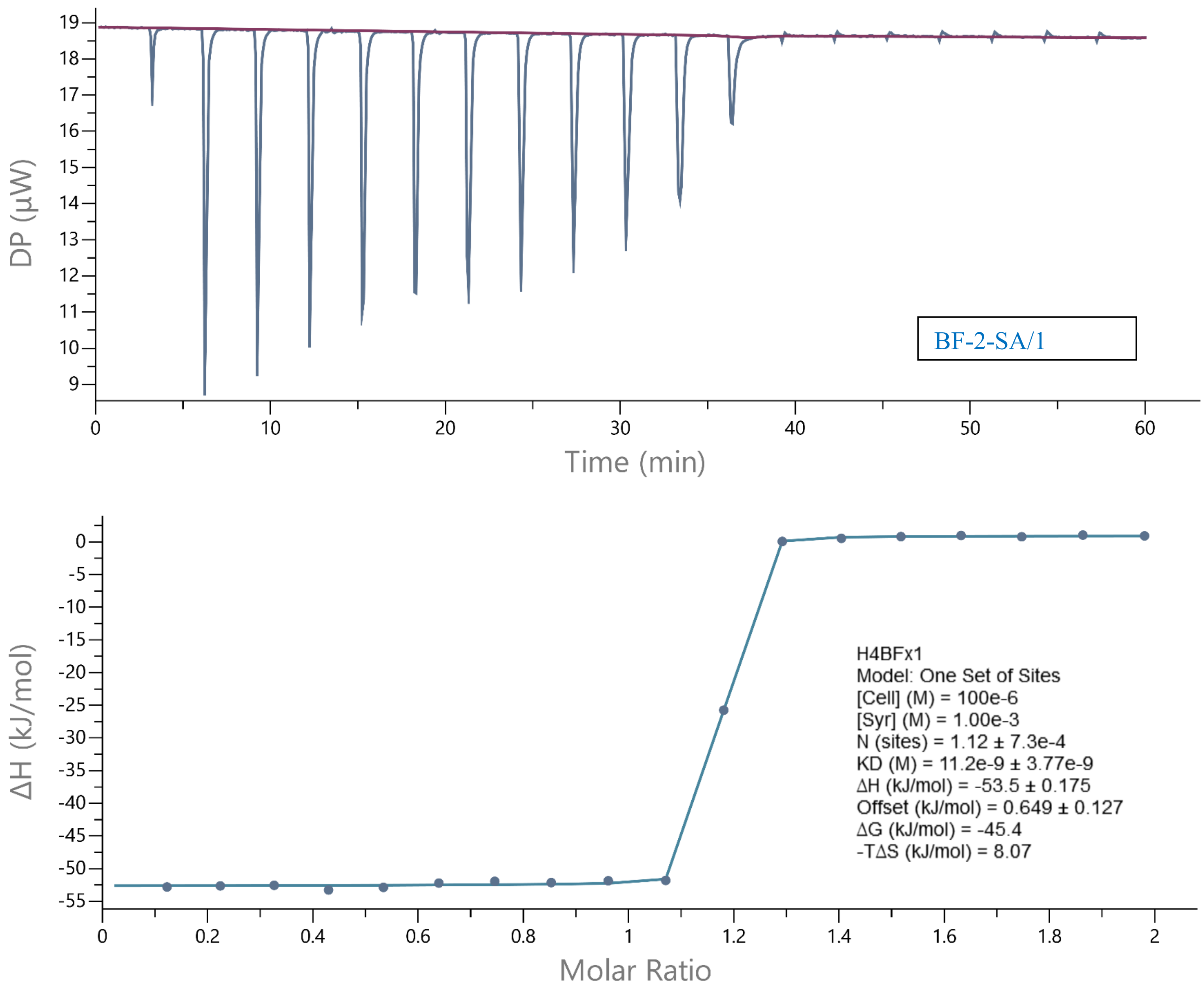

DP (μW)
Time (min)
BF-2-SA/1
ΔH (kJ/mol)
Molar Ratio
H4BFx1
Model: One Set of Sites
[Cell] (M) = 100e-6
[Syr] (M) = 1.00e-3
N (sites) = 1.12 ± 7.3e-4
KD (M) = 11.2e-9 ± 3.77e-9
ΔH (kJ/mol) = -53.5 ± 0.175
Offset (kJ/mol) = 0.649 ± 0.127
ΔG (kJ/mol) = -45.4
-TΔS (kJ/mol) = 8.07

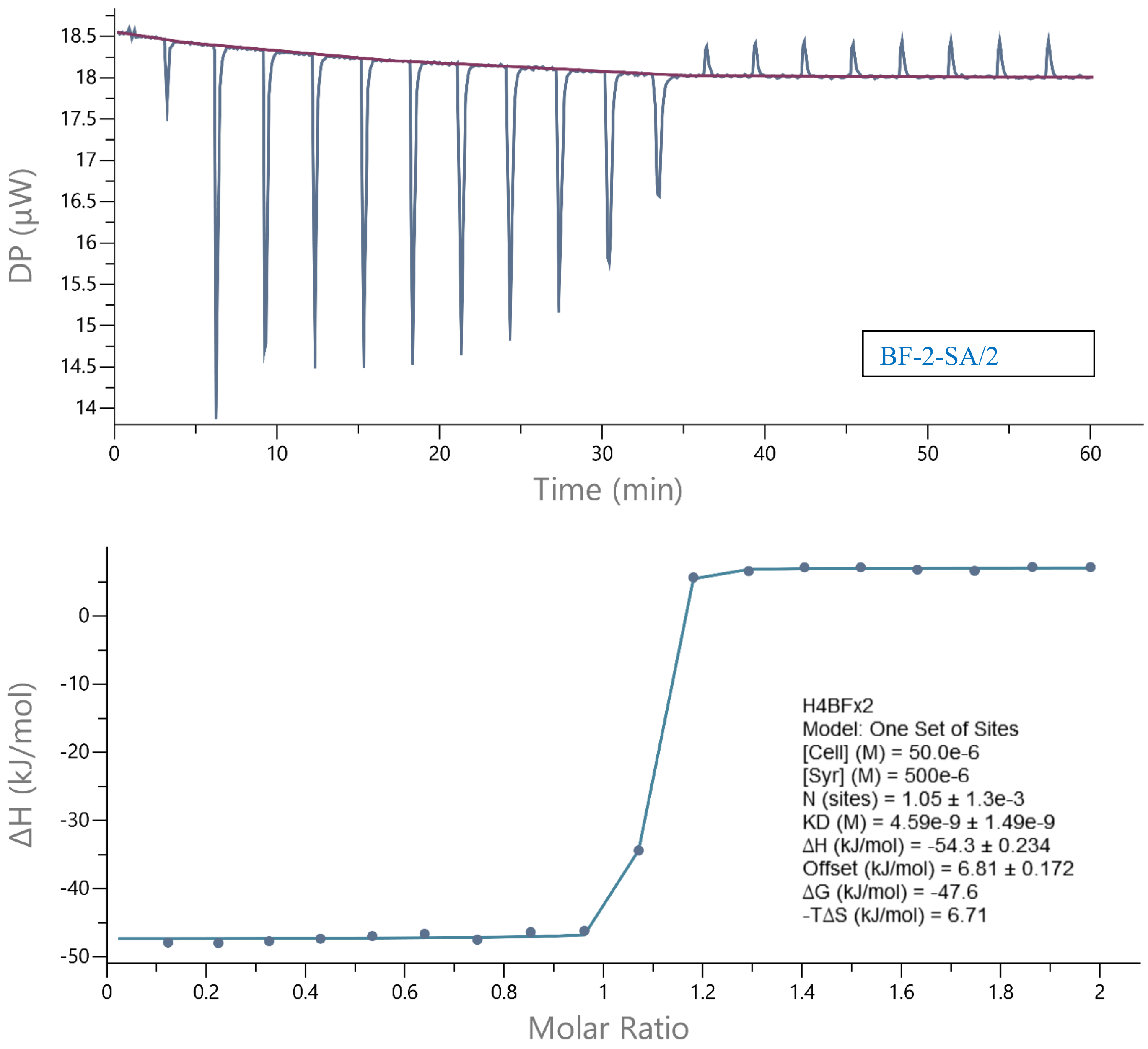
18.5
18
17.5
17
16.5
16
15.5
15
14.5
14
DP (μW)
0
10
20
30
40
50
60
Time (min)
BF-2-SA/2
0
-10
-20
-30
-40
-50
ΔH (kJ/mol)
0
0.2
0.4
0.6
0.8
1
1.2
1.4
1.6
1.8
2
Molar Ratio
H4BFx2
Model: One Set of Sites
[Cell] (M) = 50.0e-6
[Syr] (M) = 500e-6
N (sites) = 1.05 ± 1.3e-3
KD (M) = 4.59e-9 ± 1.49e-9
ΔH (kJ/mol) = -54.3 ± 0.234
Offset (kJ/mol) = 6.81 ± 0.172
ΔG (kJ/mol) = -47.6
-TΔS (kJ/mol) = 6.71

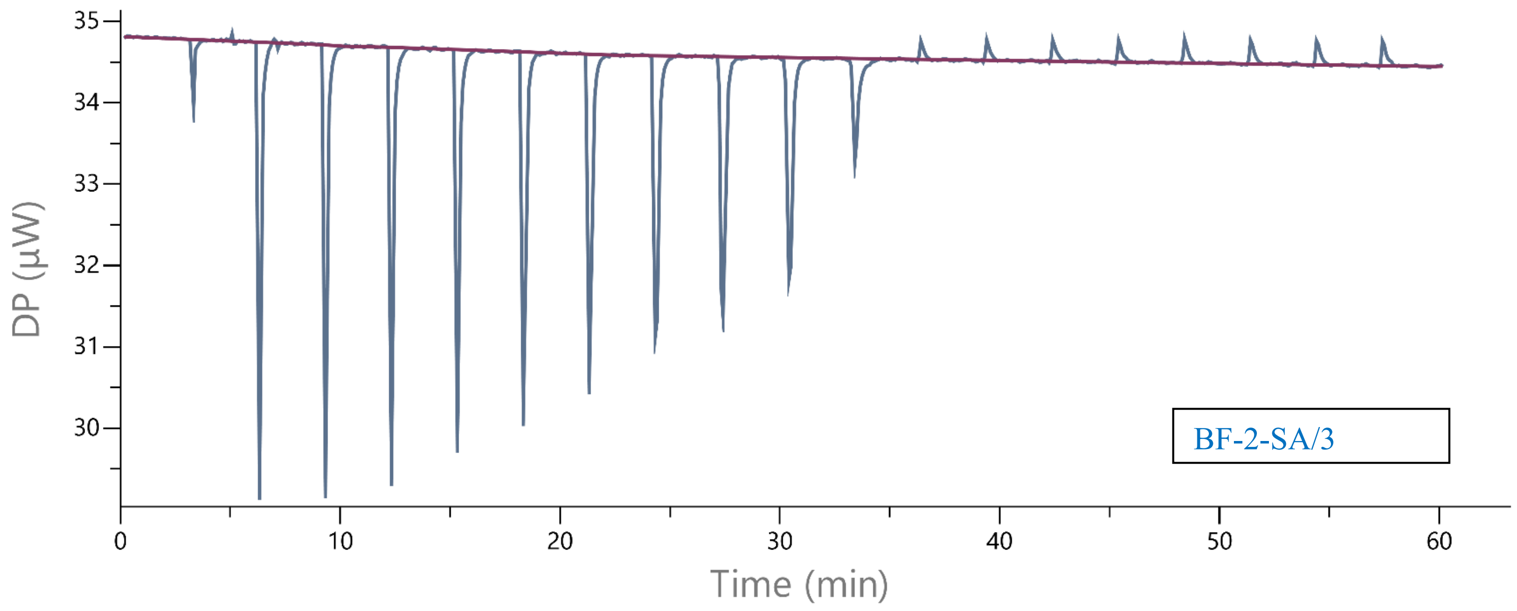
35
34
33
32
31
30
DP (μW)
0
10
20
30
40
50
60
Time (min)
BF-2-SA/3

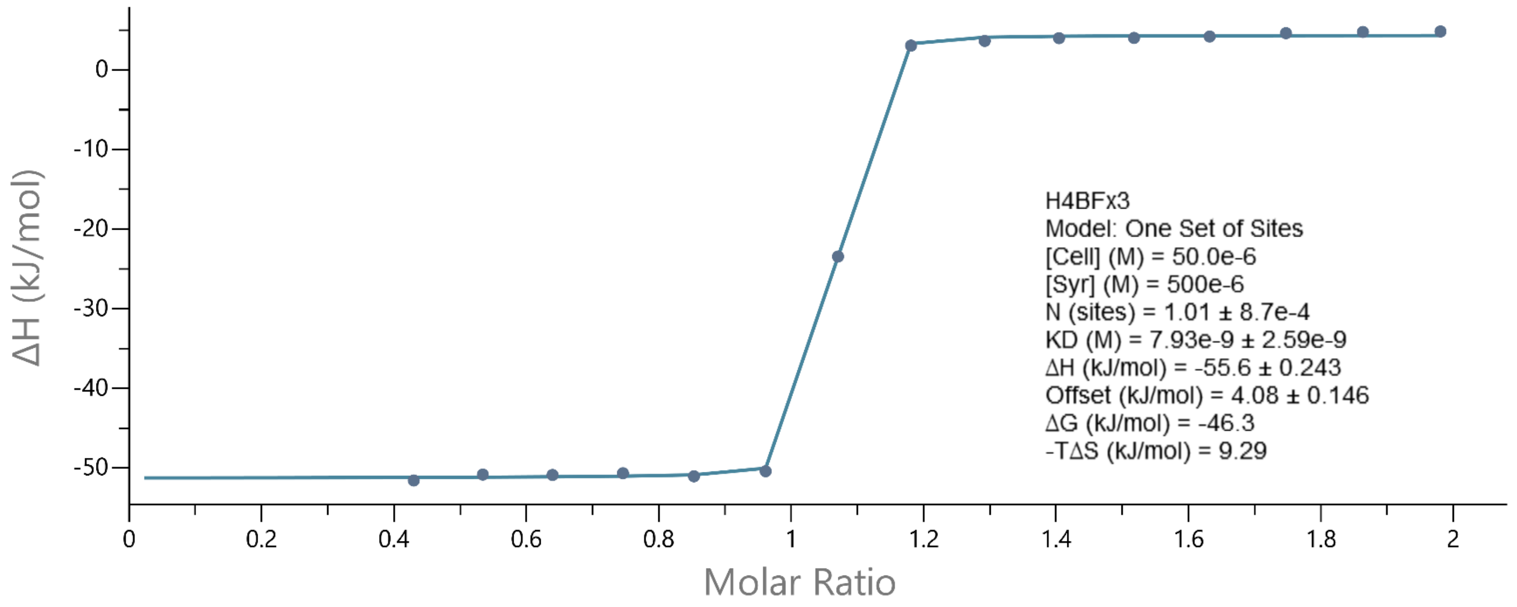
0
-10
-20
-30
-40
-50
ΔH (kJ/mol)
0
0.2
0.4
0.6
0.8
1
1.2
1.4
1.6
1.8
2
Molar Ratio
H4BFx3
Model: One Set of Sites
[Cell] (M) = 50.0e-6
[Syr] (M) = 500e-6
N (sites) = 1.01 ± 8.7e-4
KD (M) = 7.93e-9 ± 2.59e-9
ΔH (kJ/mol) = -55.6 ± 0.243
Offset (kJ/mol) = 4.08 ± 0.146
ΔG (kJ/mol) = -46.3
-TΔS (kJ/mol) = 9.29

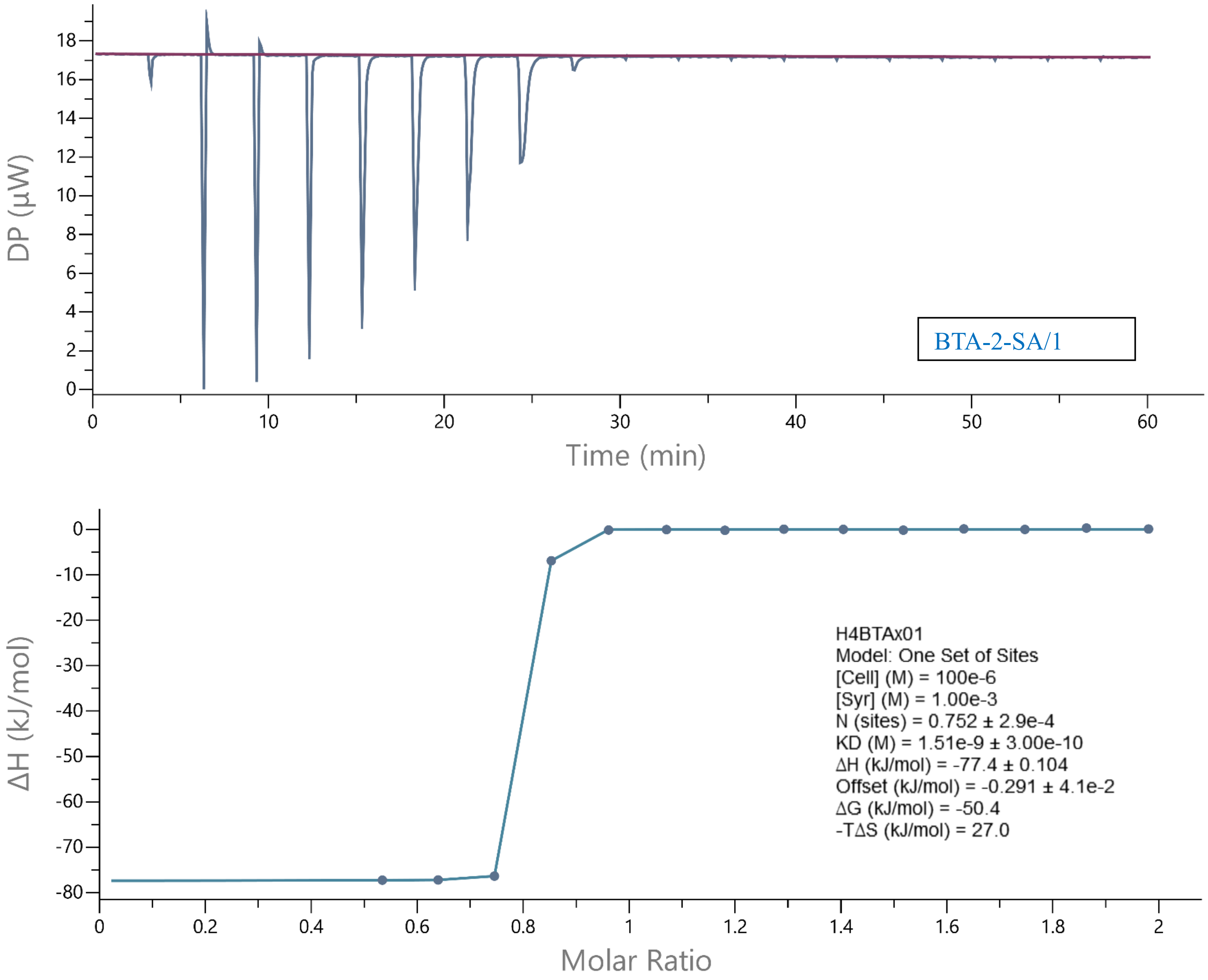
DP (μW)
Time (min)
BTA-2-SA/1
ΔH (kJ/mol)
Molar Ratio
H4BTAx01
Model: One Set of Sites
[Cell] (M) = 100e-6
[Syr] (M) = 1.00e-3
N (sites) = 0.752 ± 2.9e-4
KD (M) = 1.51e-9 ± 3.00e-10
ΔH (kJ/mol) = -77.4 ± 0.104
Offset (kJ/mol) = -0.291 ± 4.1e-2
ΔG (kJ/mol) = -50.4
-TΔS (kJ/mol) = 27.0

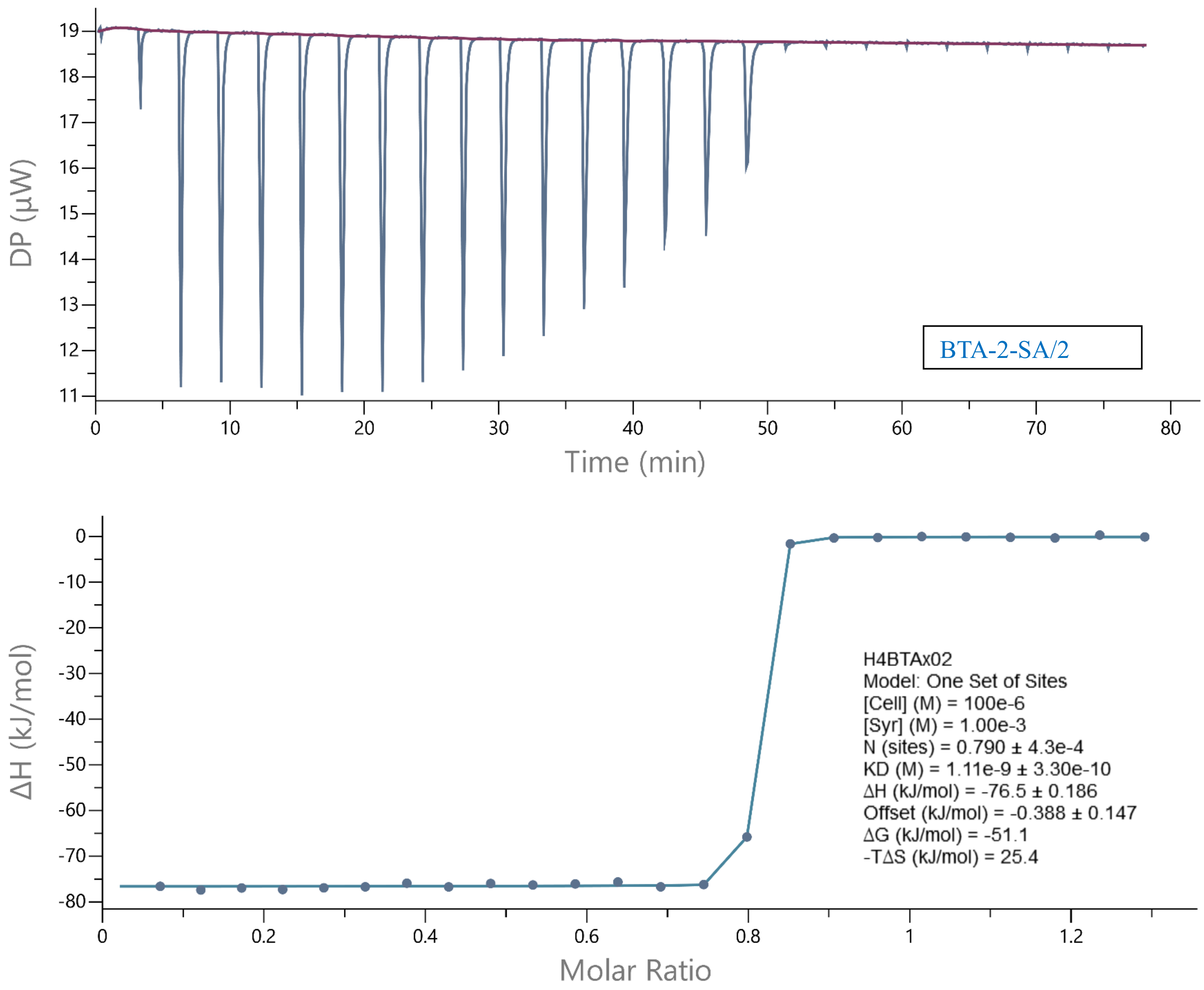

DP (μW)
Time (min)
BTA-2-SA/2
ΔH (kJ/mol)
Molar Ratio
H4BTAx02
Model: One Set of Sites
[Cell] (M) = 100e-6
[Syr] (M) = 1.00e-3
N (sites) = 0.790 ± 4.3e-4
KD (M) = 1.11e-9 ± 3.30e-10
ΔH (kJ/mol) = -76.5 ± 0.186
Offset (kJ/mol) = -0.388 ± 0.147
ΔG (kJ/mol) = -51.1
-TΔS (kJ/mol) = 25.4

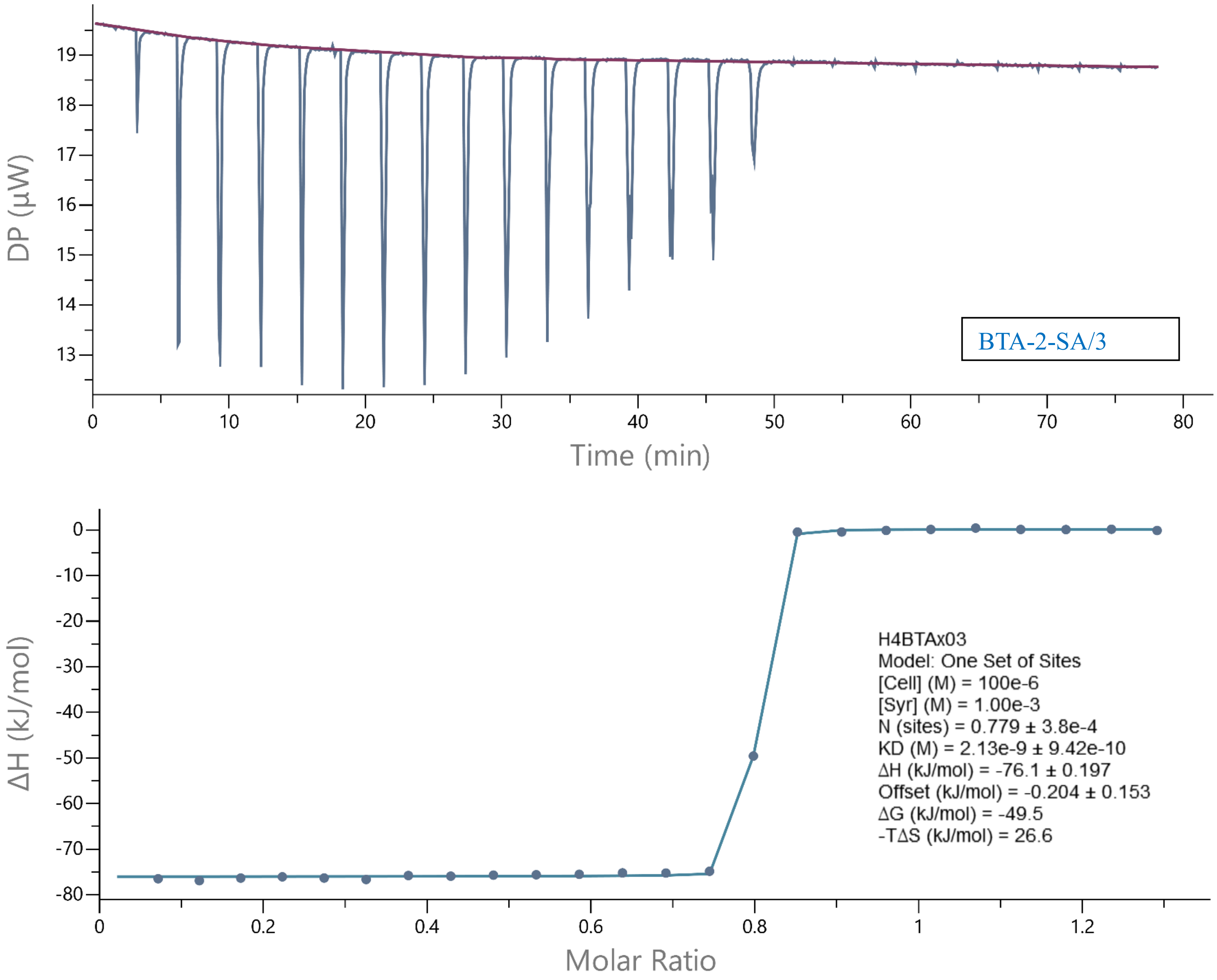
DP (μW)
Time (min)
BTA-2-SA/3
ΔH (kJ/mol)
Molar Ratio
H4BTAx03
Model: One Set of Sites
[Cell] (M) = 100e-6
[Syr] (M) = 1.00e-3
N (sites) = 0.779 ± 3.8e-4
KD (M) = 2.13e-9 ± 9.42e-10
ΔH (kJ/mol) = -76.1 ± 0.197
Offset (kJ/mol) = -0.204 ± 0.153
ΔG (kJ/mol) = -49.5
-TΔS (kJ/mol) = 26.6

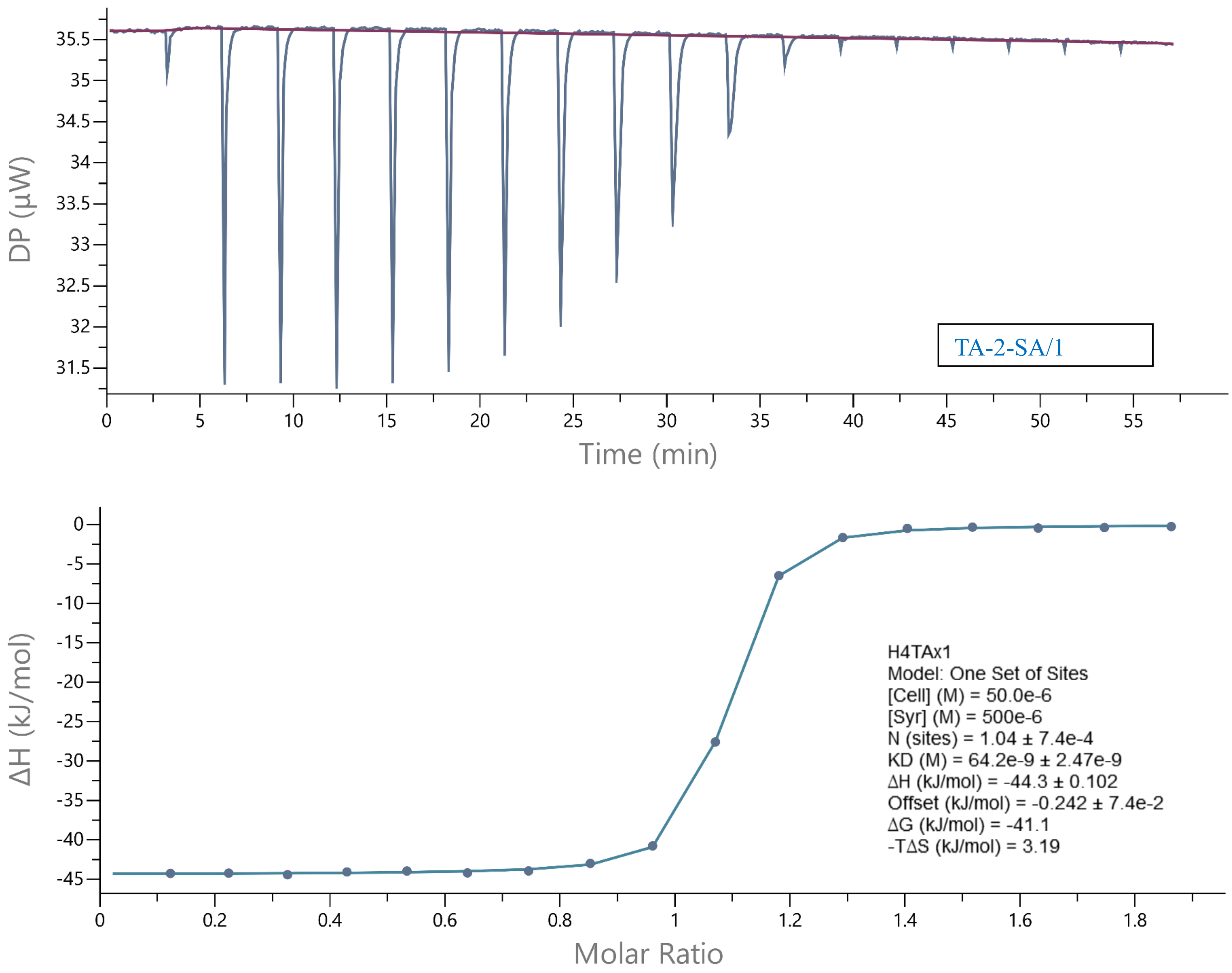

DP (μW)
35.5
35
34.5
34
33.5
33
32.5
32
31.5
0
5
10
15
20
25
30
35
40
45
50
55
Time (min)
TA-2-SA/1
ΔH (kJ/mol)
0
-5
-10
-15
-20
-25
-30
-35
-40
-45
0
0.2
0.4
0.6
0.8
1
1.2
1.4
1.6
1.8
Molar Ratio
H4TAx1
Model: One Set of Sites
[Cell] (M) = 50.0e-6
[Syr] (M) = 500e-6
N (sites) = 1.04 ± 7.4e-4
KD (M) = 64.2e-9 ± 2.47e-9
ΔH (kJ/mol) = -44.3 ± 0.102
Offset (kJ/mol) = -0.242 ± 7.4e-2
ΔG (kJ/mol) = -41.1
-TΔS (kJ/mol) = 3.19

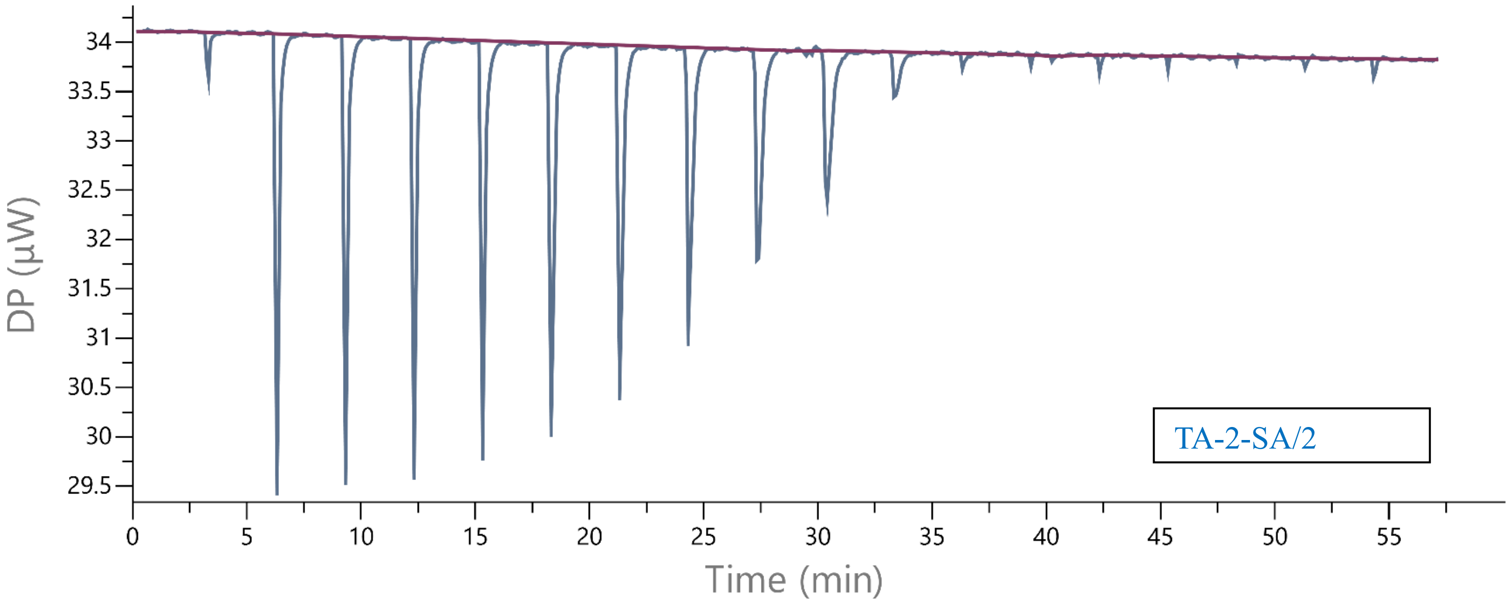
DP (μW)
Time (min)
TA-2-SA/2

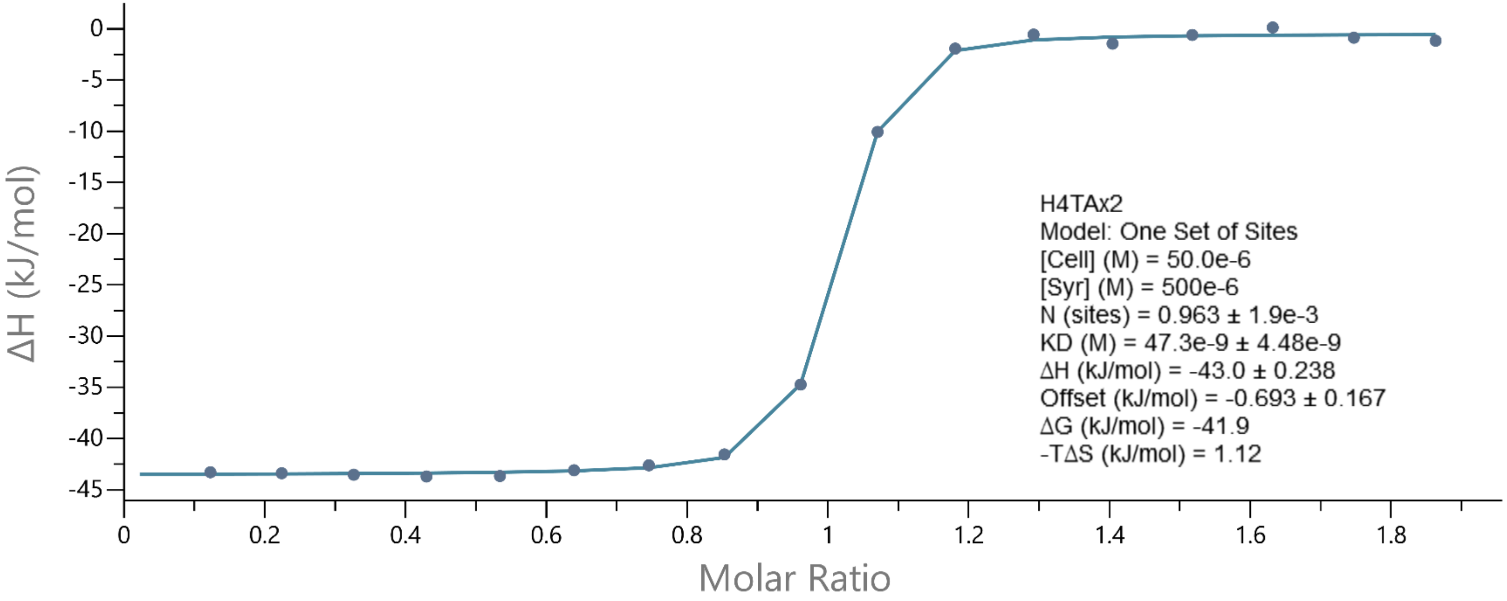
ΔH (kJ/mol)
Molar Ratio
H4TAx2
Model: One Set of Sites
[Cell] (M) = 50.0e-6
[Syr] (M) = 500e-6
N (sites) = 0.963 ± 1.9e-3
KD (M) = 47.3e-9 ± 4.48e-9
ΔH (kJ/mol) = -43.0 ± 0.238
Offset (kJ/mol) = -0.693 ± 0.167
ΔG (kJ/mol) = -41.9
-TΔS (kJ/mol) = 1.12

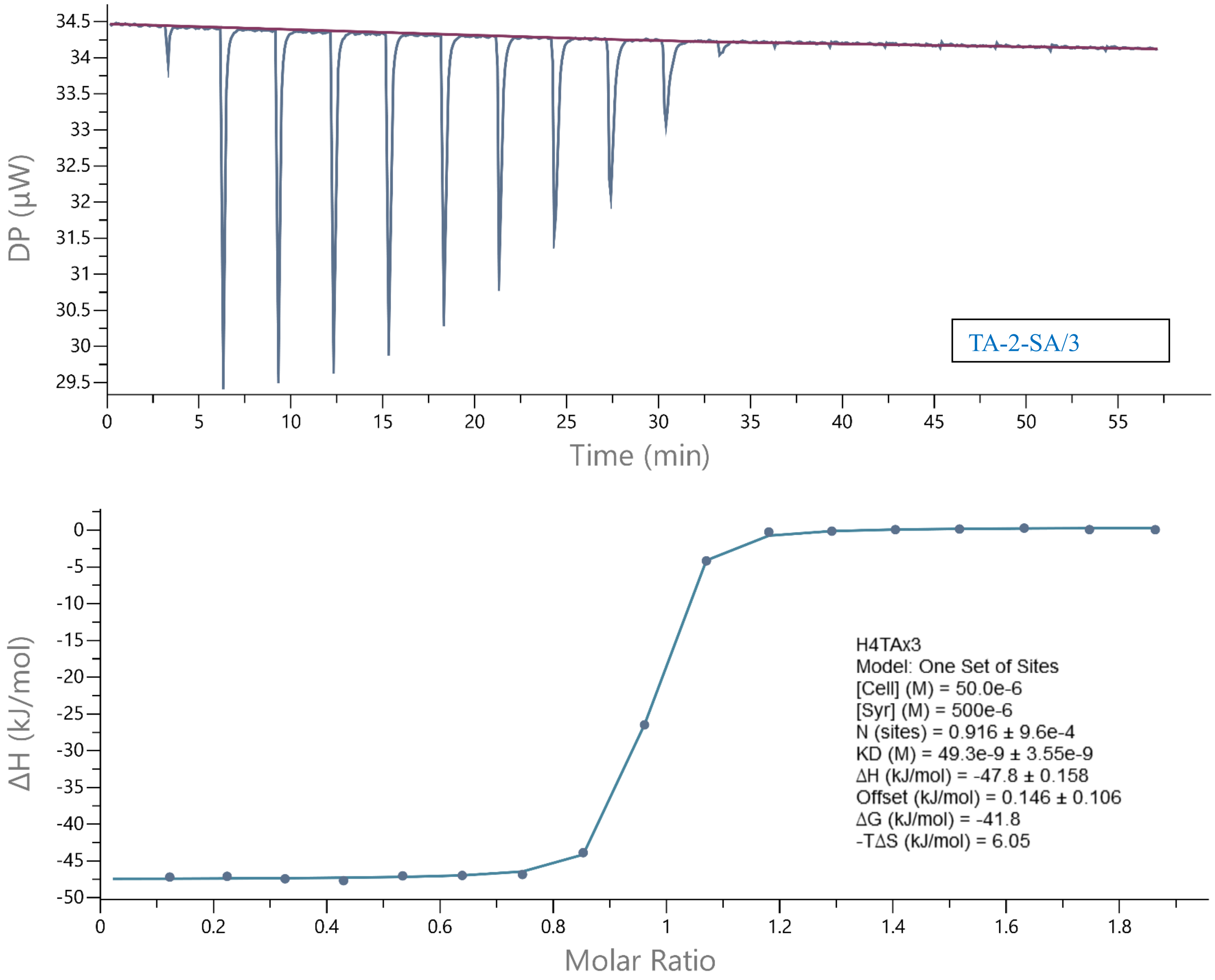
TA-2-SA/3
DP (μW)
Time (min)
ΔH (kJ/mol)
Molar Ratio
H4TAx3
Model: One Set of Sites
[Cell] (M) = 50.0e-6
[Syr] (M) = 500e-6
N (sites) = 0.916 ± 9.6e-4
KD (M) = 49.3e-9 ± 3.55e-9
ΔH (kJ/mol) = -47.8 ± 0.158
Offset (kJ/mol) = 0.146 ± 0.106
ΔG (kJ/mol) = -41.8
-TΔS (kJ/mol) = 6.05

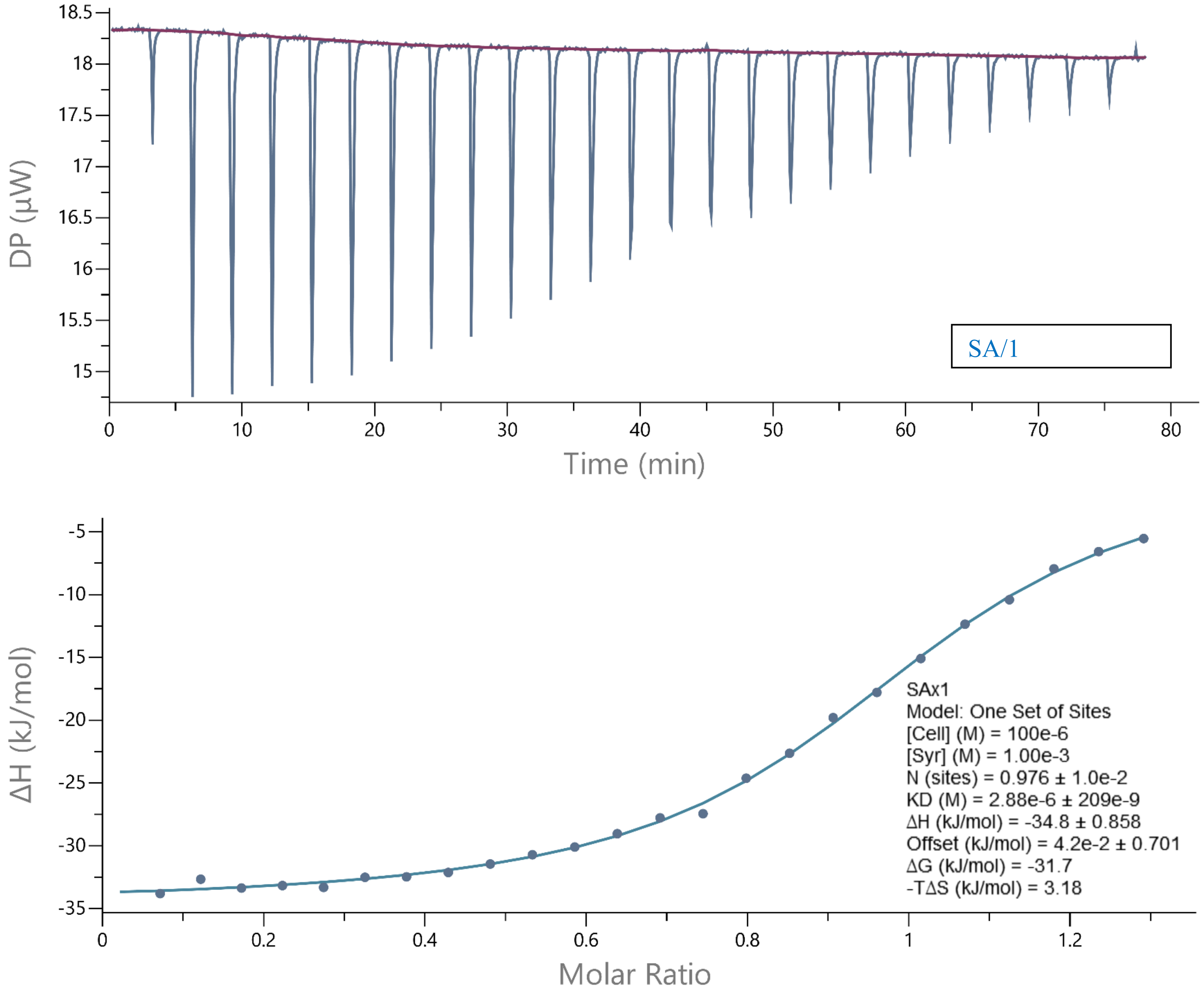

DP (μW)
Time (min)
SA/1
ΔH (kJ/mol)
Molar Ratio
SAx1
Model: One Set of Sites
[Cell] (M) = 100e-6
[Syr] (M) = 1.00e-3
N (sites) = 0.976 ± 1.0e-2
KD (M) = 2.88e-6 ± 209e-9
ΔH (kJ/mol) = -34.8 ± 0.858
Offset (kJ/mol) = 4.2e-2 ± 0.701
ΔG (kJ/mol) = -31.7
-TΔS (kJ/mol) = 3.18

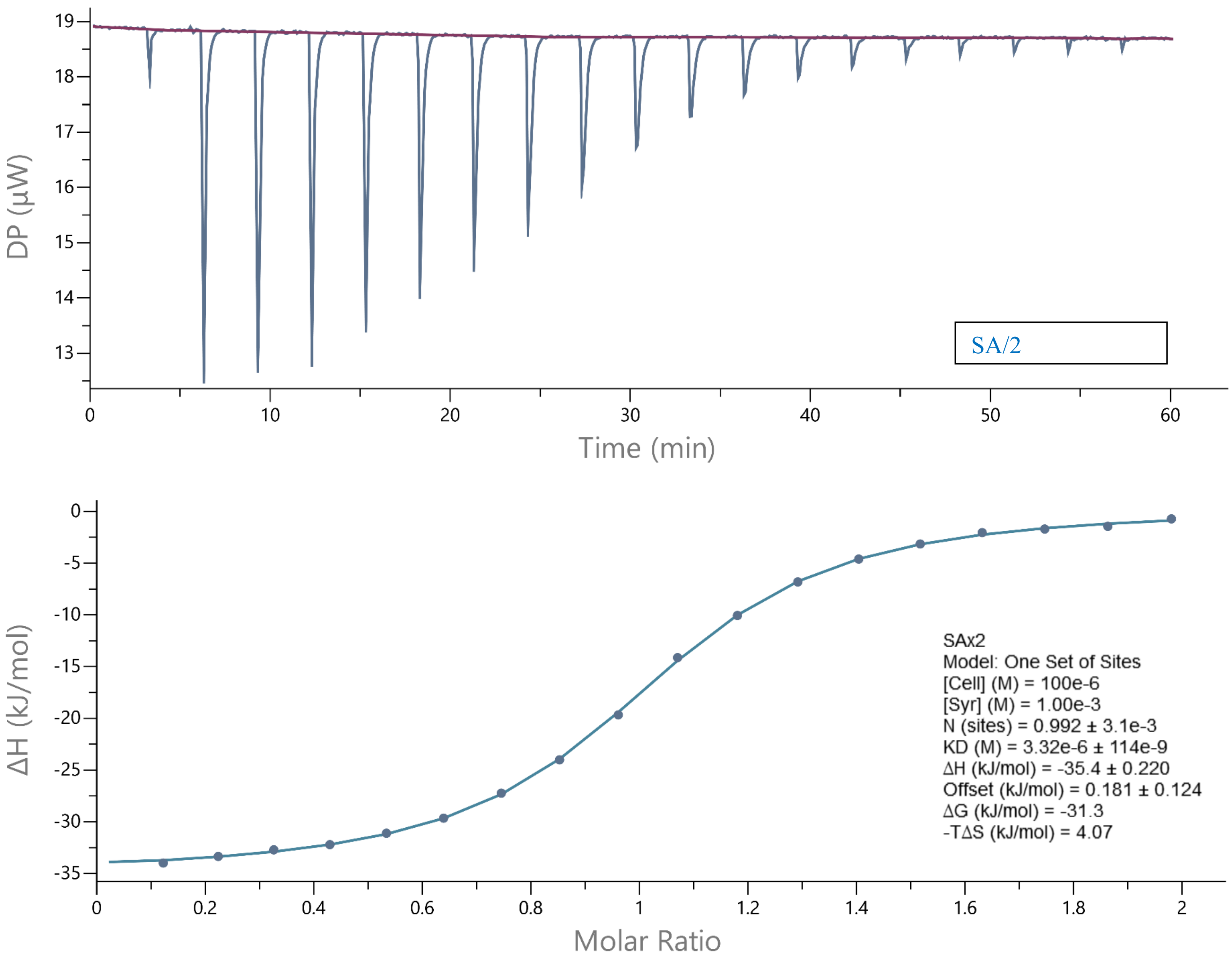
SA/2
DP (µW)
Time (min)
ΔH (kJ/mol)
Molar Ratio
SAx2
Model: One Set of Sites
[Cell] (M) = 100e-6
[Syr] (M) = 1.00e-3
N (sites) = 0.992 ± 3.1e-3
KD (M) = 3.32e-6 ± 114e-9
ΔH (kJ/mol) = -35.4 ± 0.220
Offset (kJ/mol) = 0.181 ± 0.124
ΔG (kJ/mol) = -31.3
-TΔS (kJ/mol) = 4.07

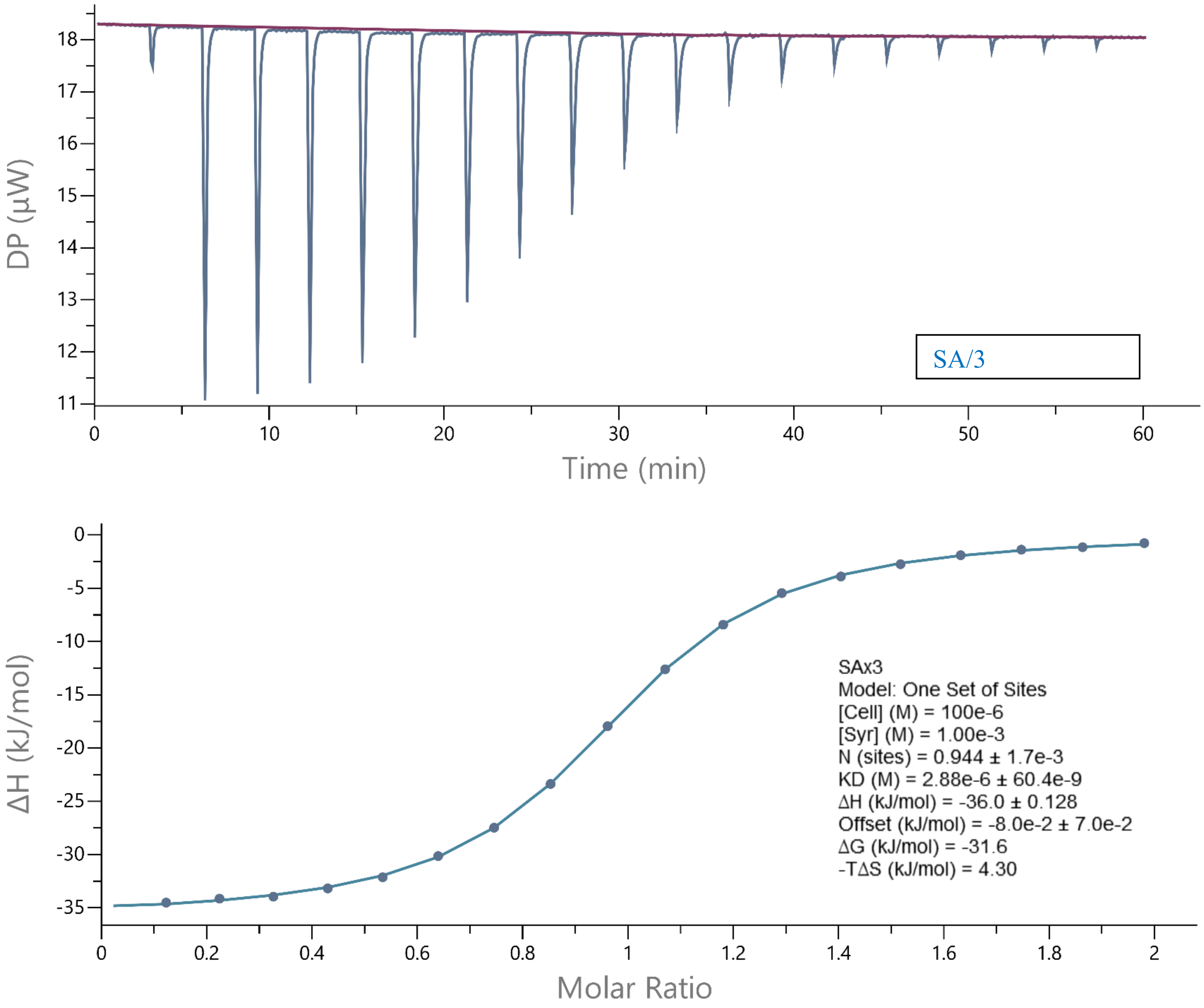
SA/3
DP (μW)
Time (min)
ΔH (kJ/mol)
Molar Ratio
SAx3
Model: One Set of Sites
[Cell] (M) = 100e-6
[Syr] (M) = 1.00e-3
N (sites) = 0.944 ± 1.7e-3
KD (M) = 2.88e-6 ± 60.4e-9
ΔH (kJ/mol) = -36.0 ± 0.128
Offset (kJ/mol) = -8.0e-2 ± 7.0e-2
ΔG (kJ/mol) = -31.6
-TΔS (kJ/mol) = 4.30

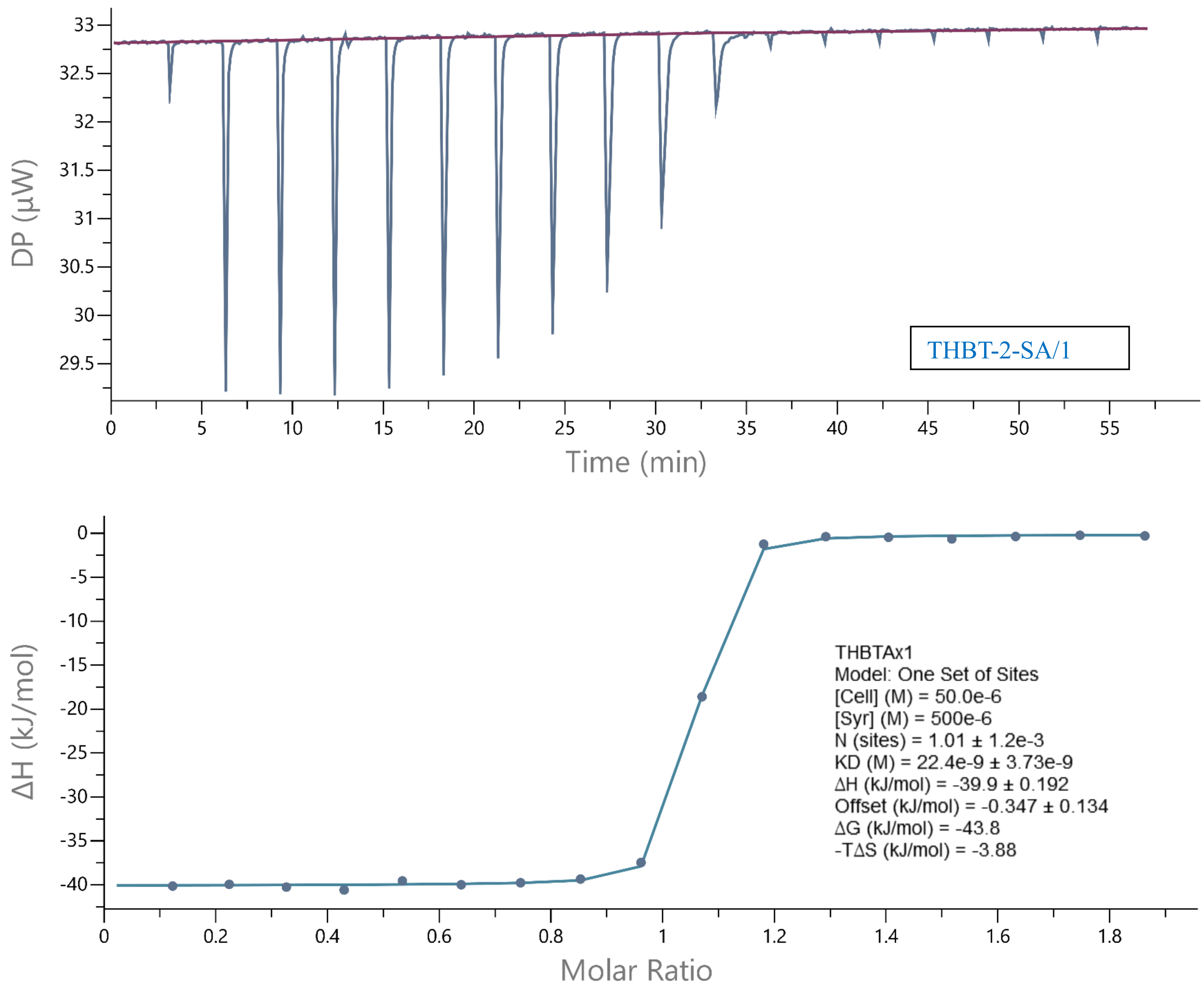

THBT-2-SA/1
DP (μW)
Time (min)
ΔH (kJ/mol)
Molar Ratio
THBTAx1
Model: One Set of Sites
[Cell] (M) = 50.0e-6
[Syr] (M) = 500e-6
N (sites) = 1.01 ± 1.2e-3
KD (M) = 22.4e-9 ± 3.73e-9
ΔH (kJ/mol) = -39.9 ± 0.192
Offset (kJ/mol) = -0.347 ± 0.134
ΔG (kJ/mol) = -43.8
-TΔS (kJ/mol) = -3.88

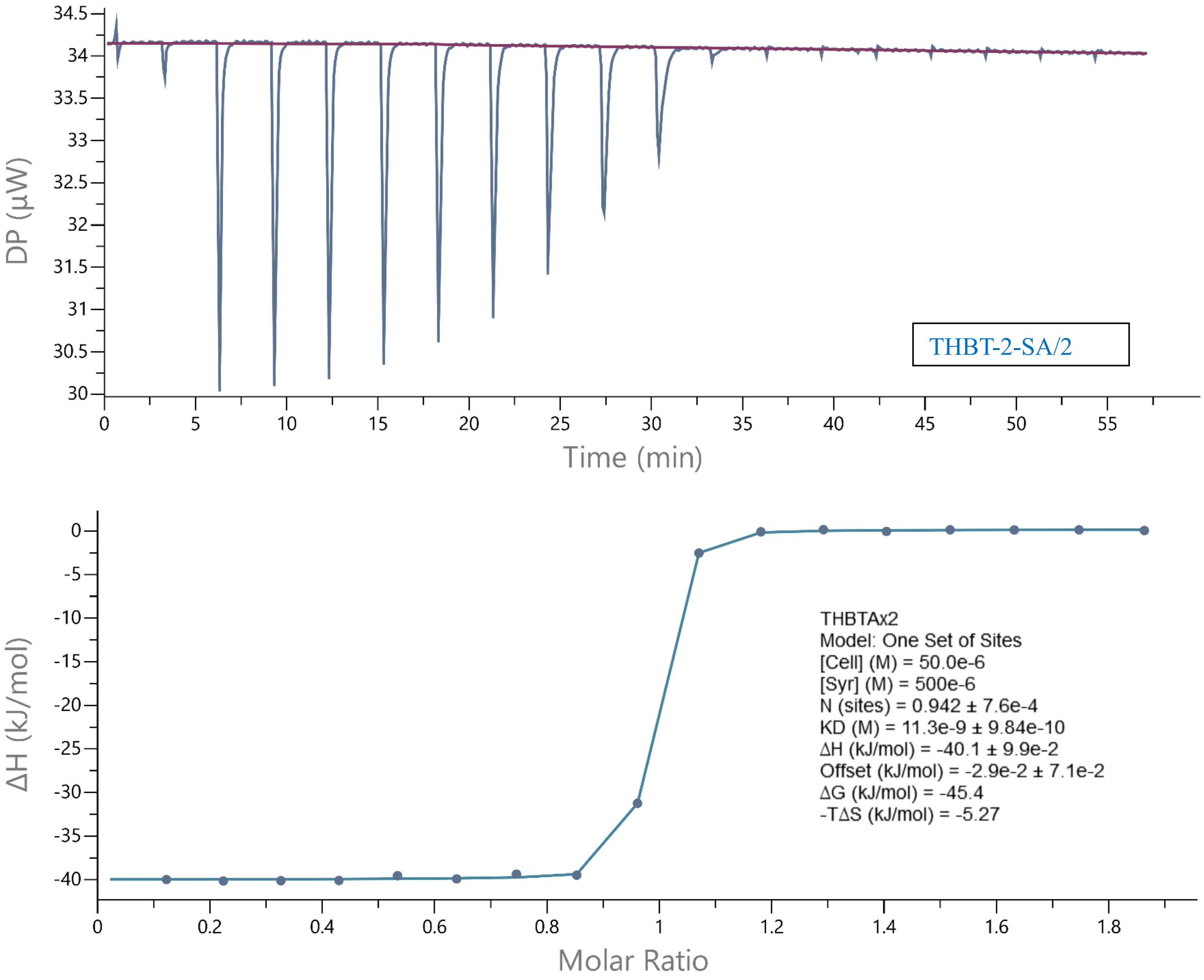

DP (μW)
Time (min)
THBT-2-SA/2
ΔH (kJ/mol)
Molar Ratio
THBTAx2
Model: One Set of Sites
[Cell] (M) = 50.0e-6
[Syr] (M) = 500e-6
N (sites) = 0.942 ± 7.6e-4
KD (M) = 11.3e-9 ± 9.84e-10
ΔH (kJ/mol) = -40.1 ± 9.9e-2
Offset (kJ/mol) = -2.9e-2 ± 7.1e-2
ΔG (kJ/mol) = -45.4
-TΔS (kJ/mol) = -5.27

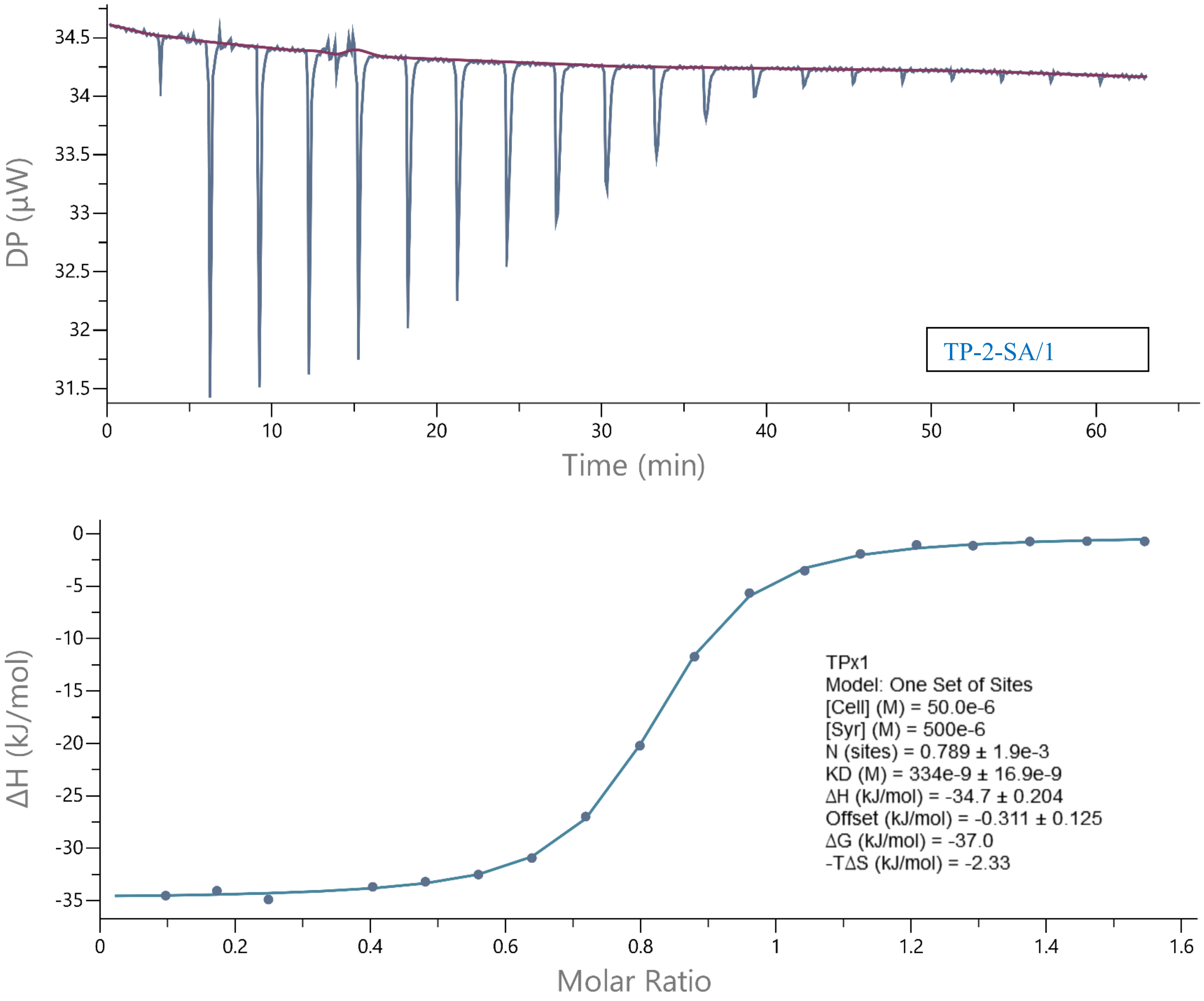

34.5
34
33.5
33
32.5
32
31.5
DP (μW)
0
10
20
30
40
50
60
Time (min)
TP-2-SA/1
0
-5
-10
-15
-20
-25
-30
-35
ΔH (kJ/mol)
0
0.2
0.4
0.6
0.8
1
1.2
1.4
1.6
Molar Ratio
TPx1
Model: One Set of Sites
[Cell] (M) = 50.0e-6
[Syr] (M) = 500e-6
N (sites) = 0.789 ± 1.9e-3
KD (M) = 334e-9 ± 16.9e-9
ΔH (kJ/mol) = -34.7 ± 0.204
Offset (kJ/mol) = -0.311 ± 0.125
ΔG (kJ/mol) = -37.0
-TΔS (kJ/mol) = -2.33

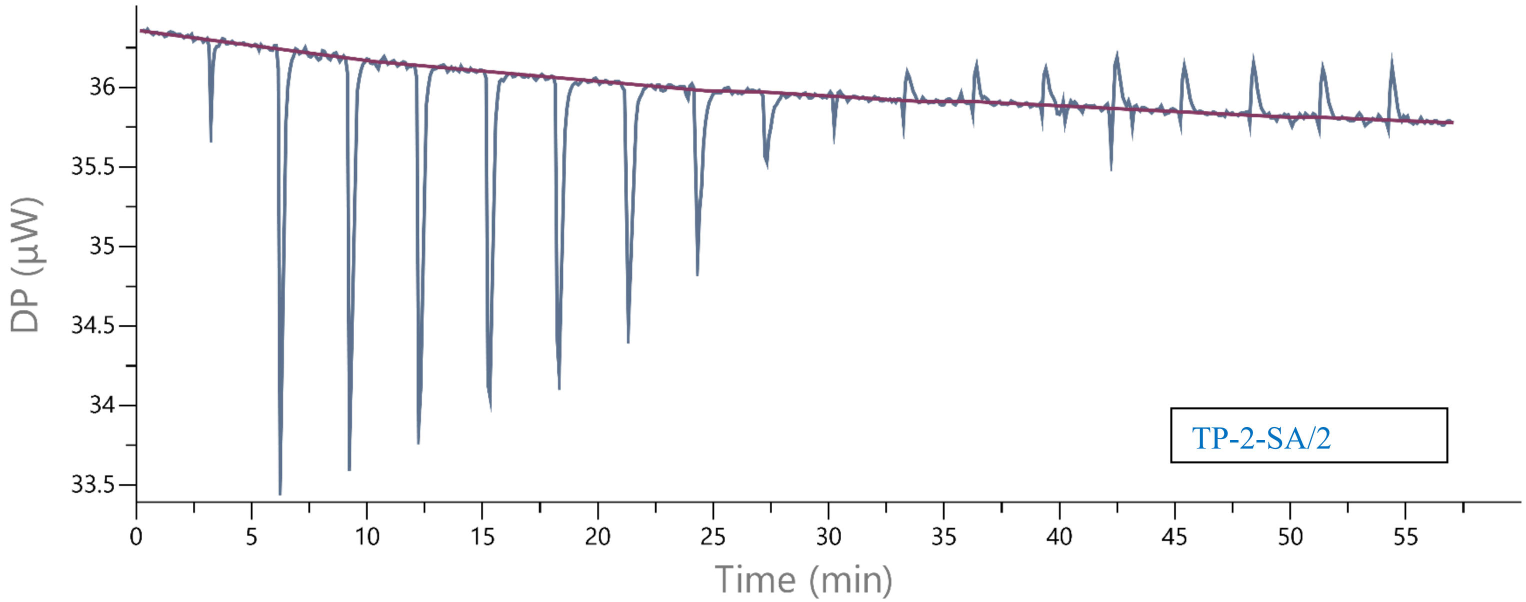

DP (μW)
36
35.5
35
34.5
34
33.5
0
5
10
15
20
25
30
35
40
45
50
55
Time (min)
TP-2-SA/2

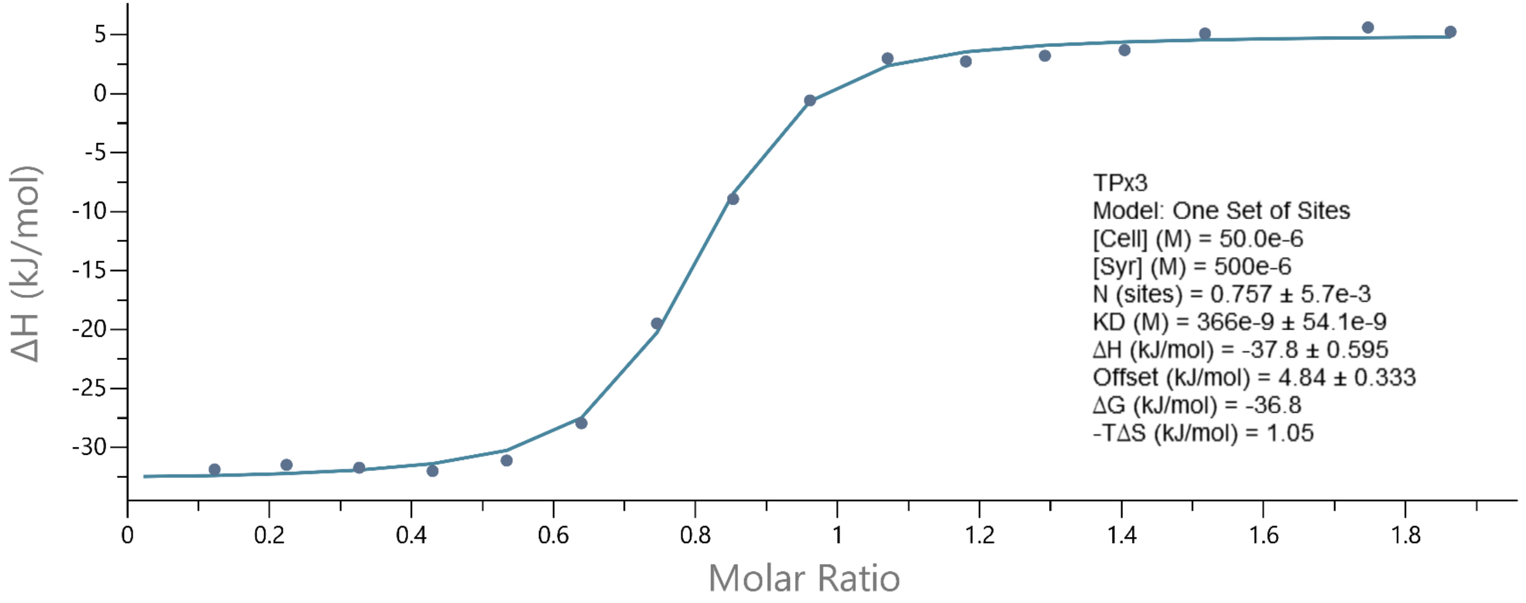

ΔH (kJ/mol)
5
0
-5
-10
-15
-20
-25
-30
0
0.2
0.4
0.6
0.8
1
1.2
1.4
1.6
1.8
Molar Ratio
TPx3
Model: One Set of Sites
[Cell] (M) = 50.0e-6
[Syr] (M) = 500e-6
N (sites) = 0.757 ± 5.7e-3
KD (M) = 366e-9 ± 54.1e-9
ΔH (kJ/mol) = -37.8 ± 0.595
Offset (kJ/mol) = 4.84 ± 0.333
ΔG (kJ/mol) = -36.8
-TΔS (kJ/mol) = 1.05

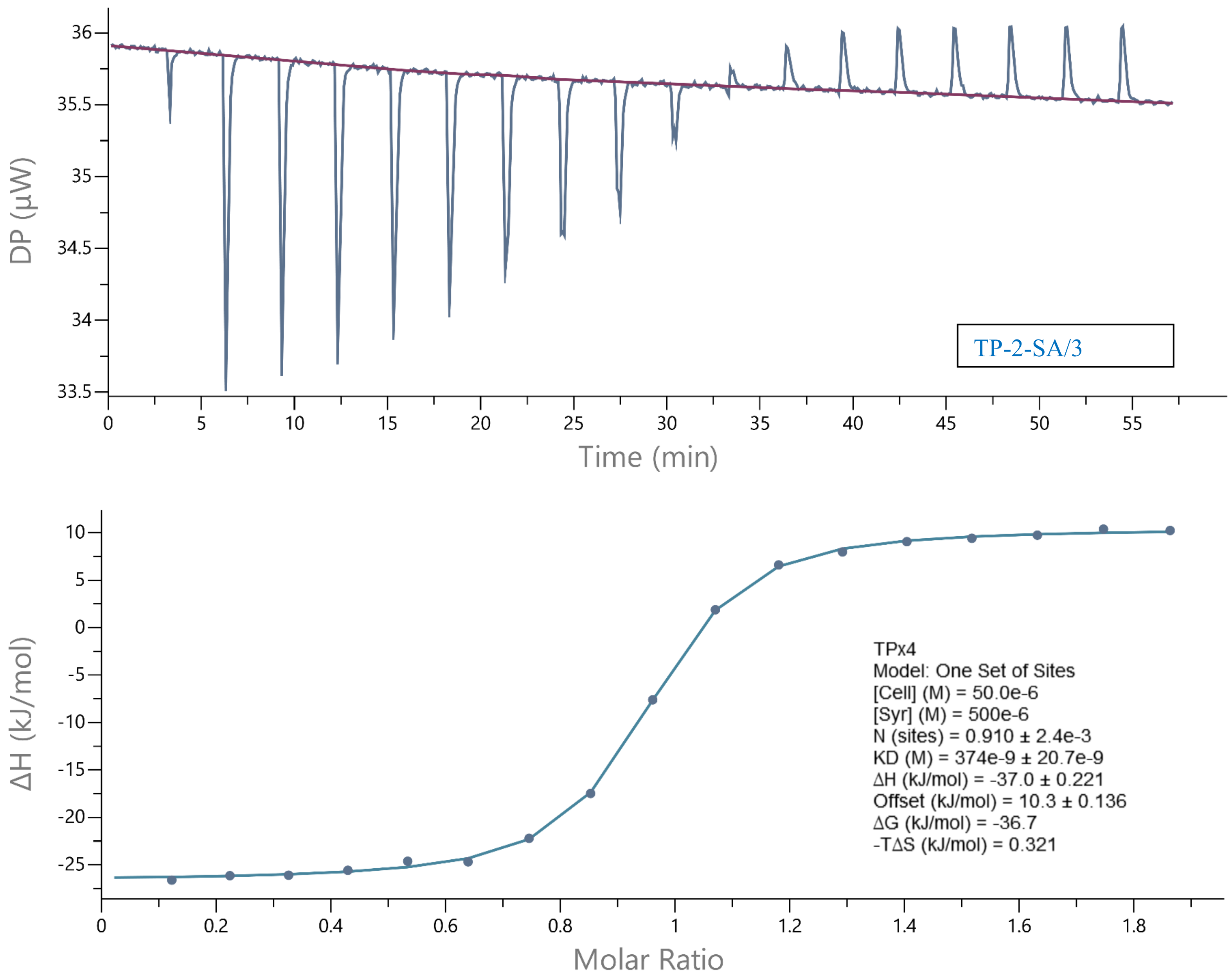

DP (μW)
Time (min)
TP-2-SA/3
ΔH (kJ/mol)
Molar Ratio
TPx4
Model: One Set of Sites
[Cell] (M) = 50.0e-6
[Syr] (M) = 500e-6
N (sites) = 0.910 ± 2.4e-3
KD (M) = 374e-9 ± 20.7e-9
ΔH (kJ/mol) = -37.0 ± 0.221
Offset (kJ/mol) = 10.3 ± 0.136
ΔG (kJ/mol) = -36.7
-TΔS (kJ/mol) = 0.321

3.5. ITC results for CAII measurements in D_2O

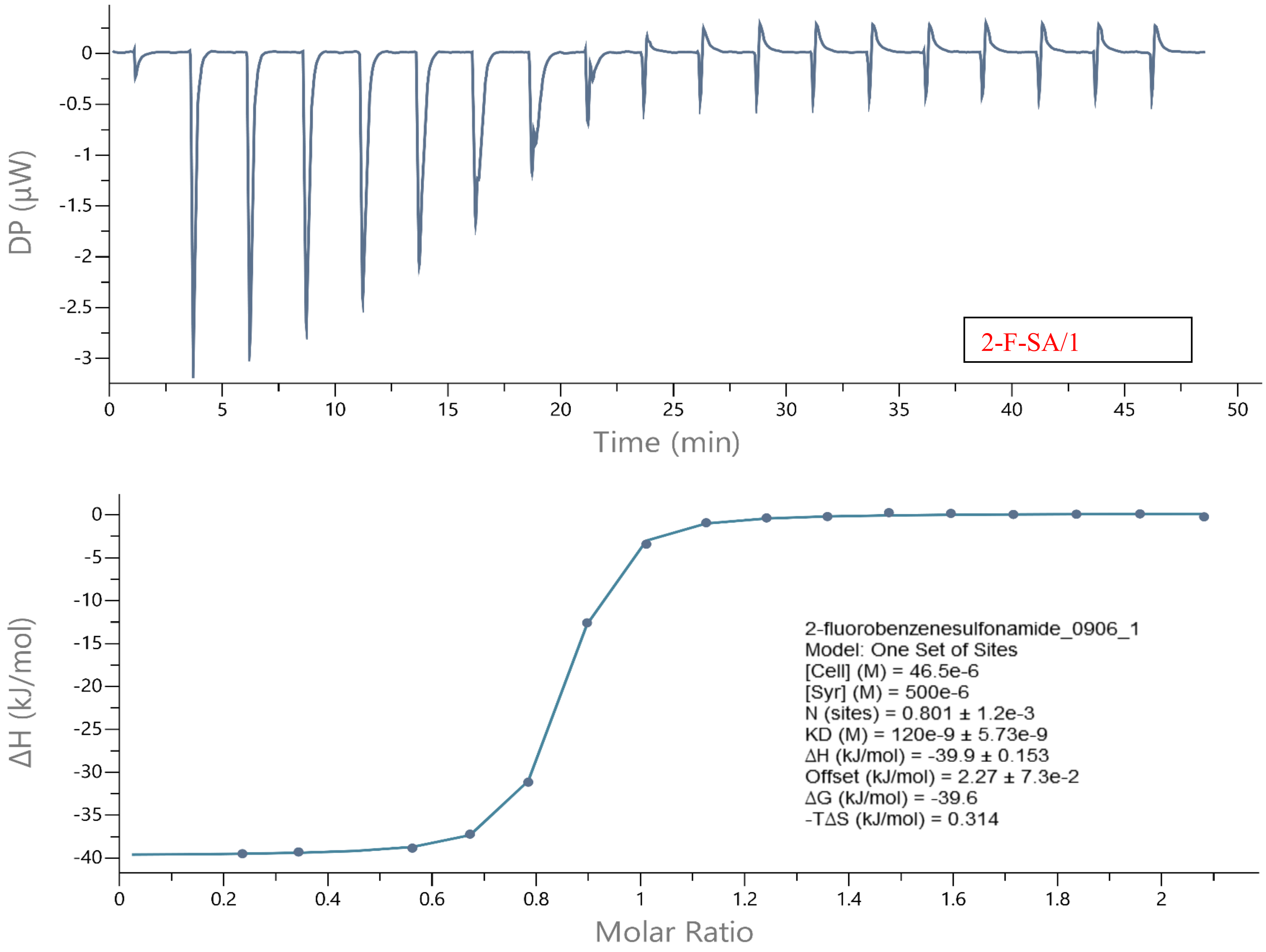

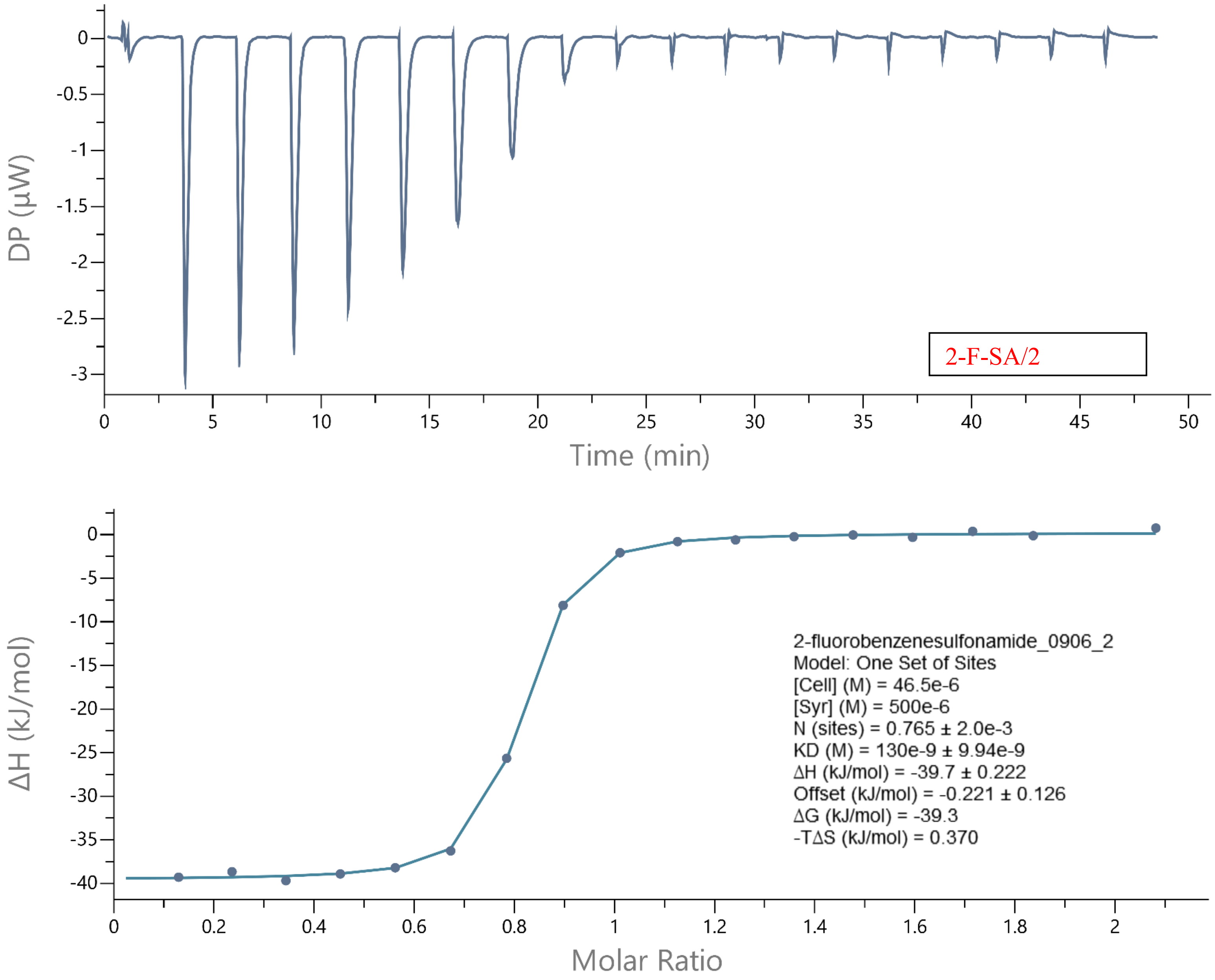
DP (µW)
Time (min)
2-F-SA/2
ΔH (kJ/mol)
Molar Ratio
2-fluorobenzenesulfonamide_0906_2
Model: One Set of Sites
[Cell] (M) = 46.5e-6
[Syr] (M) = 500e-6
N (sites) = 0.765 ± 2.0e-3
KD (M) = 130e-9 ± 9.94e-9
ΔH (kJ/mol) = -39.7 ± 0.222
Offset (kJ/mol) = -0.221 ± 0.126
ΔG (kJ/mol) = -39.3
-TΔS (kJ/mol) = 0.370

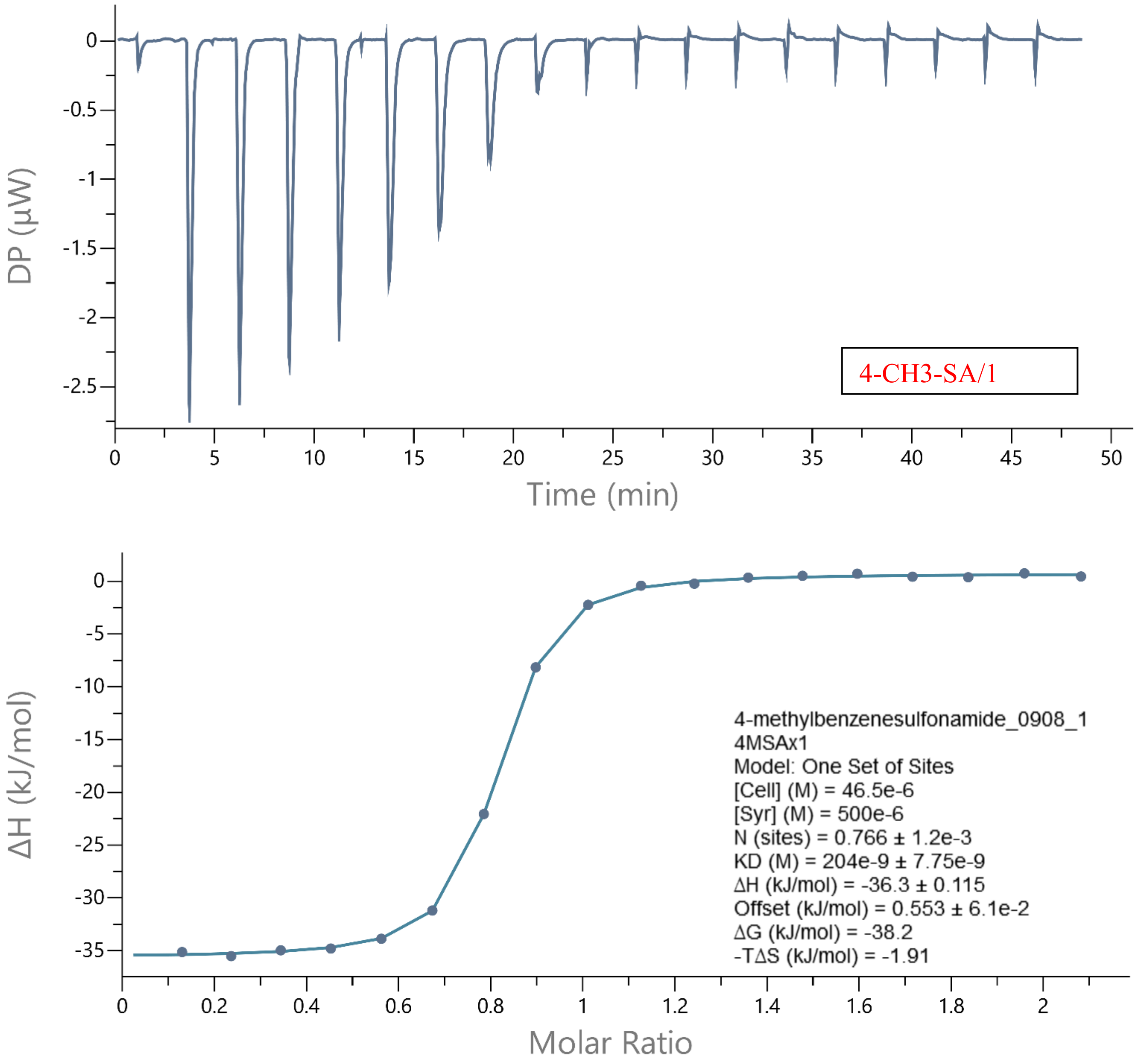

DP (μW)
0
-0.5
-1
-1.5
-2
-2.5
0
5
10
15
20
25
30
35
40
45
50
Time (min)
4-CH3-SA/1
ΔH (kJ/mol)
0
-5
-10
-15
-20
-25
-30
-35
0
0.2
0.4
0.6
0.8
1
1.2
1.4
1.6
1.8
2
Molar Ratio
4-methylbenzenesulfonamide_0908_1
4MSAx1
Model: One Set of Sites
[Cell] (M) = 46.5e-6
[Syr] (M) = 500e-6
N (sites) = 0.766 ± 1.2e-3
KD (M) = 204e-9 ± 7.75e-9
ΔH (kJ/mol) = -36.3 ± 0.115
Offset (kJ/mol) = 0.553 ± 6.1e-2
ΔG (kJ/mol) = -38.2
-TΔS (kJ/mol) = -1.91

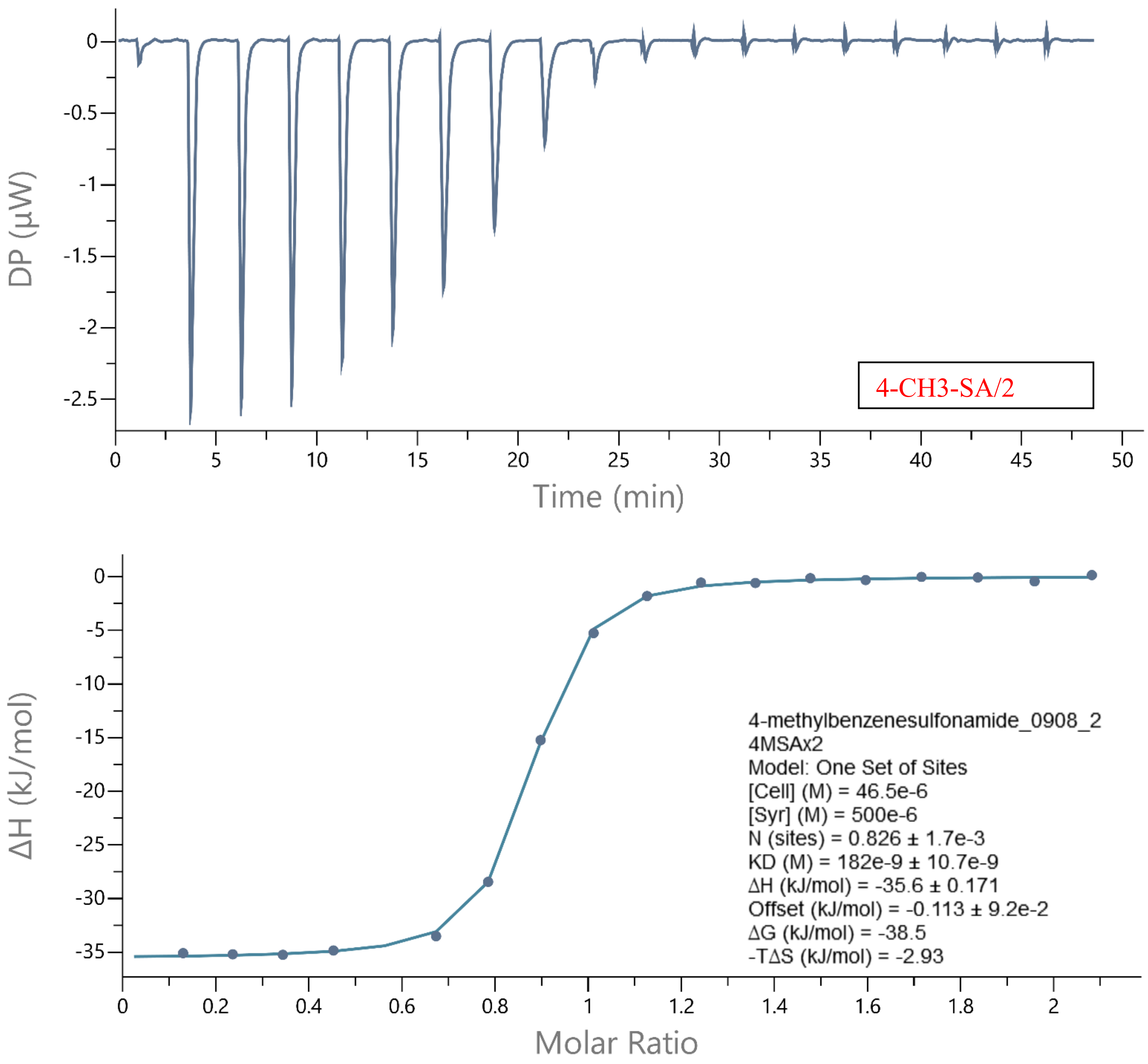

DP (μW)
Time (min)
4-CH3-SA/2
ΔH (kJ/mol)
Molar Ratio
4-methylbenzenesulfonamide_0908_2
4MSAx2
Model: One Set of Sites
[Cell] (M) = 46.5e-6
[Syr] (M) = 500e-6
N (sites) = 0.826 ± 1.7e-3
KD (M) = 182e-9 ± 10.7e-9
ΔH (kJ/mol) = -35.6 ± 0.171
Offset (kJ/mol) = -0.113 ± 9.2e-2
ΔG (kJ/mol) = -38.5
-TΔS (kJ/mol) = -2.93

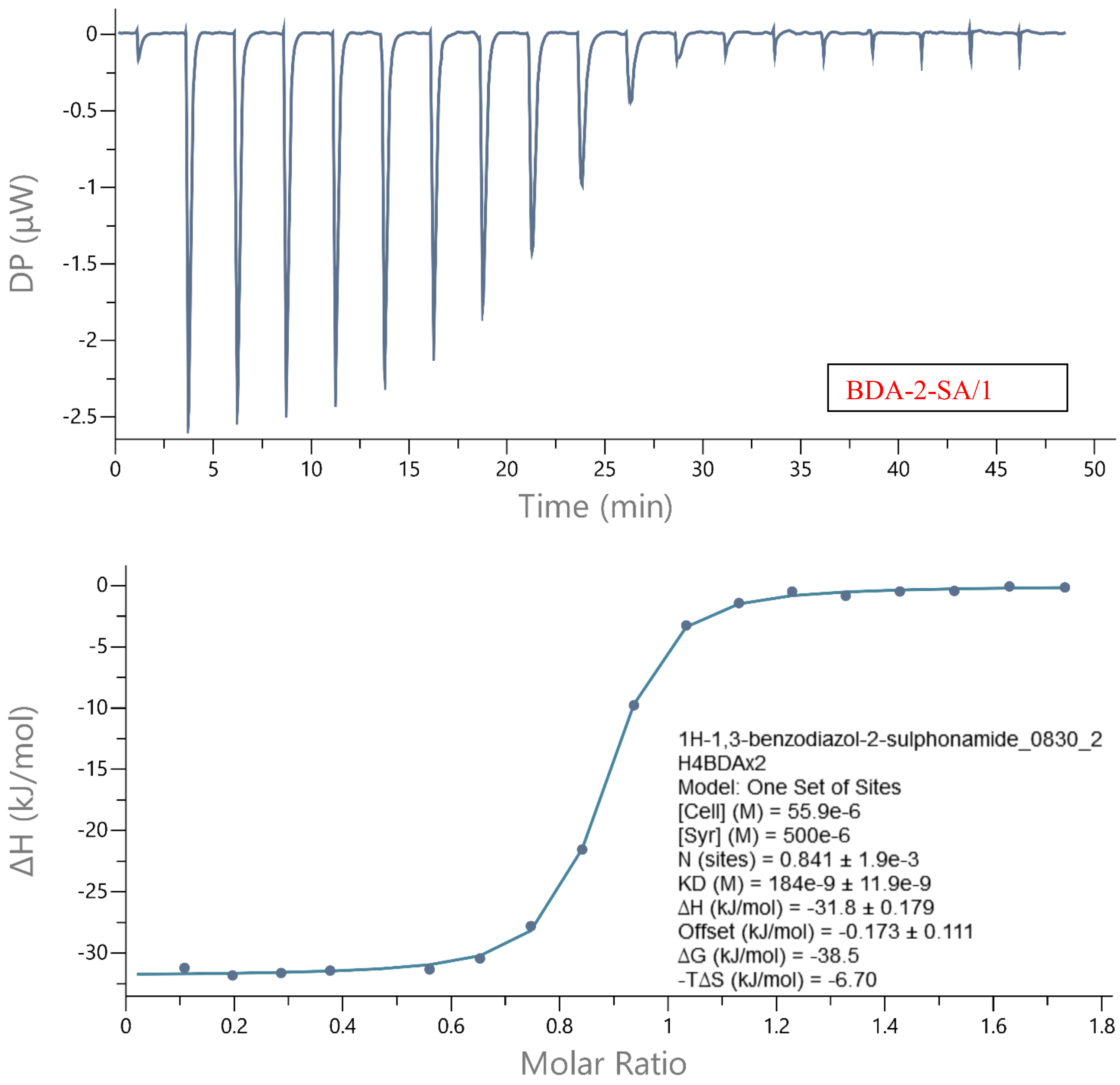

DP (μW)
Time (min)
BDA-2-SA/1
ΔH (kJ/mol)
Molar Ratio
1H-1,3-benzodiazol-2-sulphonamide_0830_2
H4BDAx2
Model: One Set of Sites
[Cell] (M) = 55.9e-6
[Syr] (M) = 500e-6
N (sites) = 0.841 ± 1.9e-3
KD (M) = 184e-9 ± 11.9e-9
ΔH (kJ/mol) = -31.8 ± 0.179
Offset (kJ/mol) = -0.173 ± 0.111
ΔG (kJ/mol) = -38.5
-TΔS (kJ/mol) = -6.70

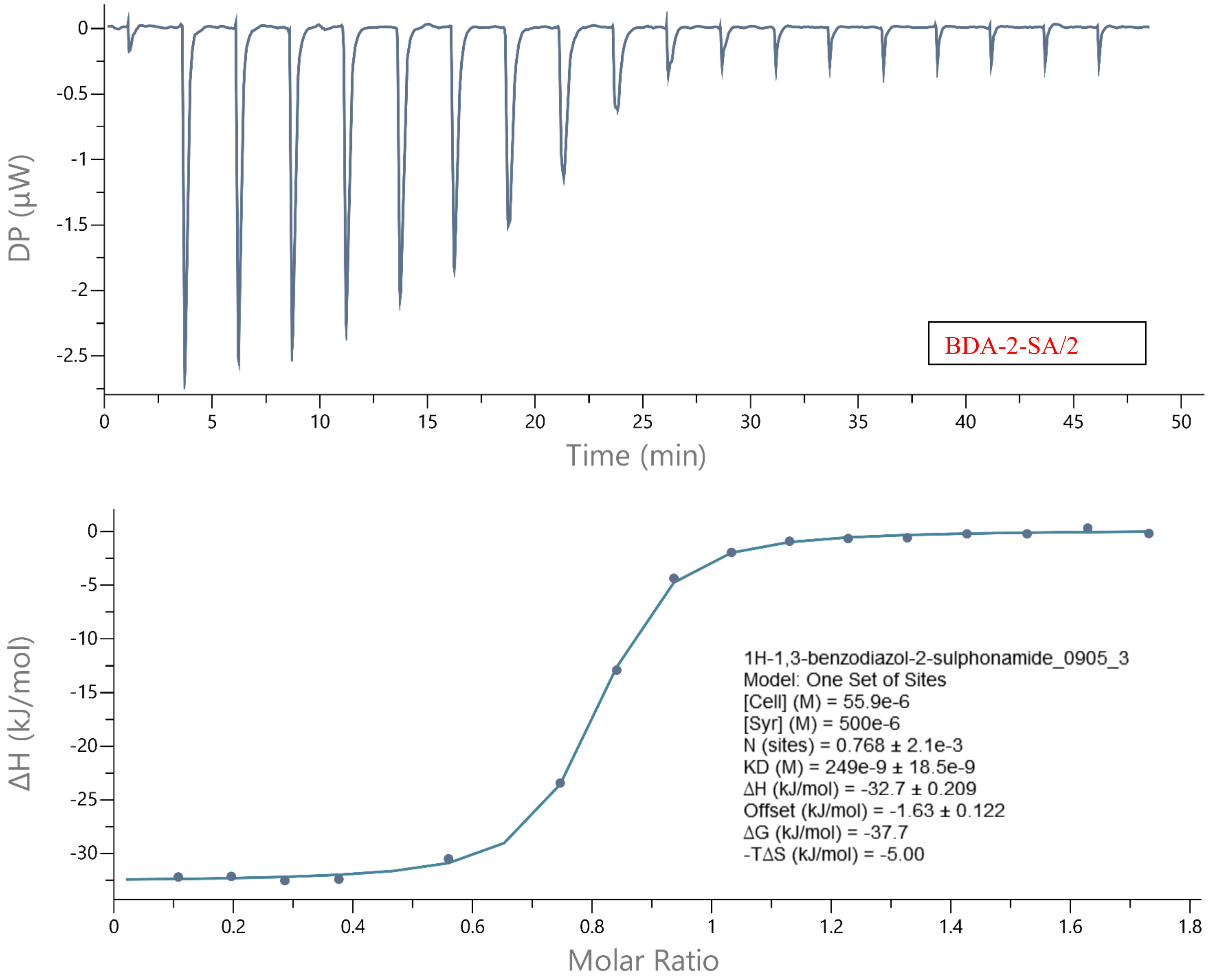

DP (μW)
Time (min)
BDA-2-SA/2
ΔH (kJ/mol)
Molar Ratio
1H-1,3-benzodiazol-2-sulphonamide_0905_3
Model: One Set of Sites
[Cell] (M) = 55.9e-6
[Syr] (M) = 500e-6
N (sites) = 0.768 ± 2.1e-3
KD (M) = 249e-9 ± 18.5e-9
ΔH (kJ/mol) = -32.7 ± 0.209
Offset (kJ/mol) = -1.63 ± 0.122
ΔG (kJ/mol) = -37.7
-TΔS (kJ/mol) = -5.00

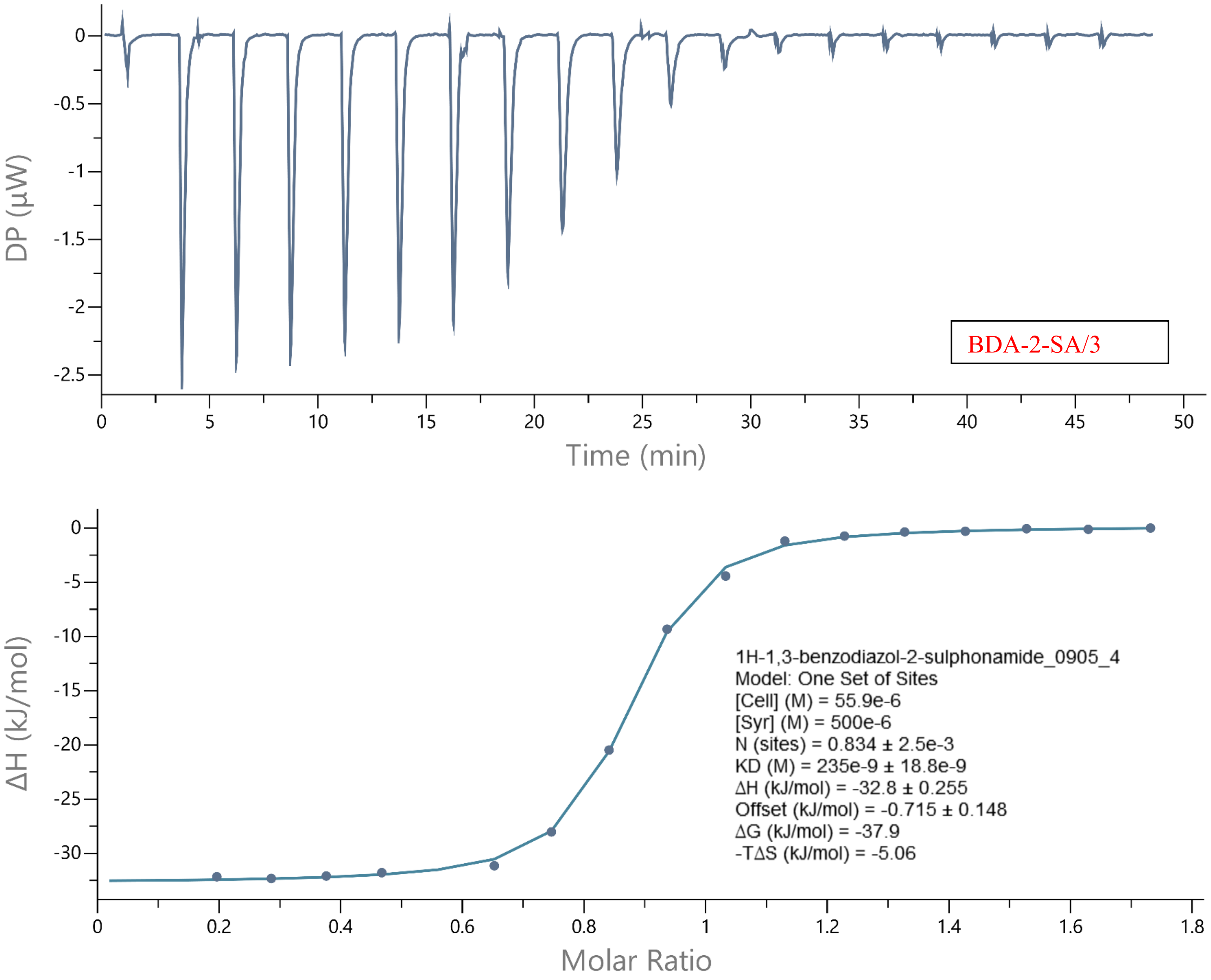

DP (µW)
0
-0.5
-1
-1.5
-2
-2.5
0
5
10
15
20
25
30
35
40
45
50
Time (min)
BDA-2-SA/3
ΔH (kJ/mol)
0
-5
-10
-15
-20
-25
-30
0
0.2
0.4
0.6
0.8
1
1.2
1.4
1.6
1.8
Molar Ratio
1H-1,3-benzodiazol-2-sulphonamide_0905_4
Model: One Set of Sites
[Cell] (M) = 55.9e-6
[Syr] (M) = 500e-6
N (sites) = 0.834 ± 2.5e-3
KD (M) = 235e-9 ± 18.8e-9
ΔH (kJ/mol) = -32.8 ± 0.255
Offset (kJ/mol) = -0.715 ± 0.148
ΔG (kJ/mol) = -37.9
-TΔS (kJ/mol) = -5.06

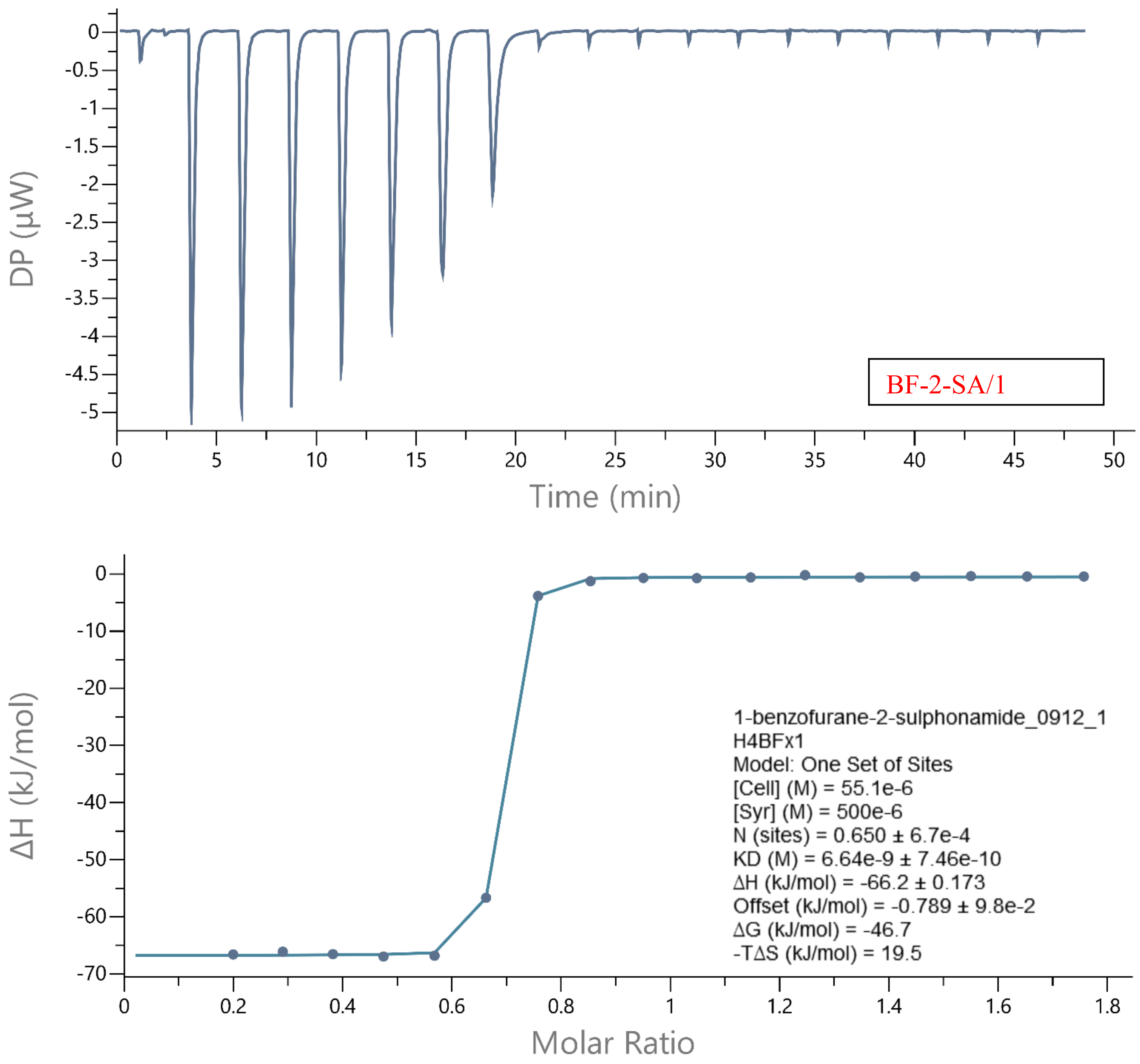

BF-2-SA/1
DP (μW)
Time (min)
ΔH (kJ/mol)
Molar Ratio
1-benzofurane-2-sulphonamide_0912_1
H4BFx1
Model: One Set of Sites
[Cell] (M) = 55.1e-6
[Syr] (M) = 500e-6
N (sites) = 0.650 ± 6.7e-4
KD (M) = 6.64e-9 ± 7.46e-10
ΔH (kJ/mol) = -66.2 ± 0.173
Offset (kJ/mol) = -0.789 ± 9.8e-2
ΔG (kJ/mol) = -46.7
-TΔS (kJ/mol) = 19.5

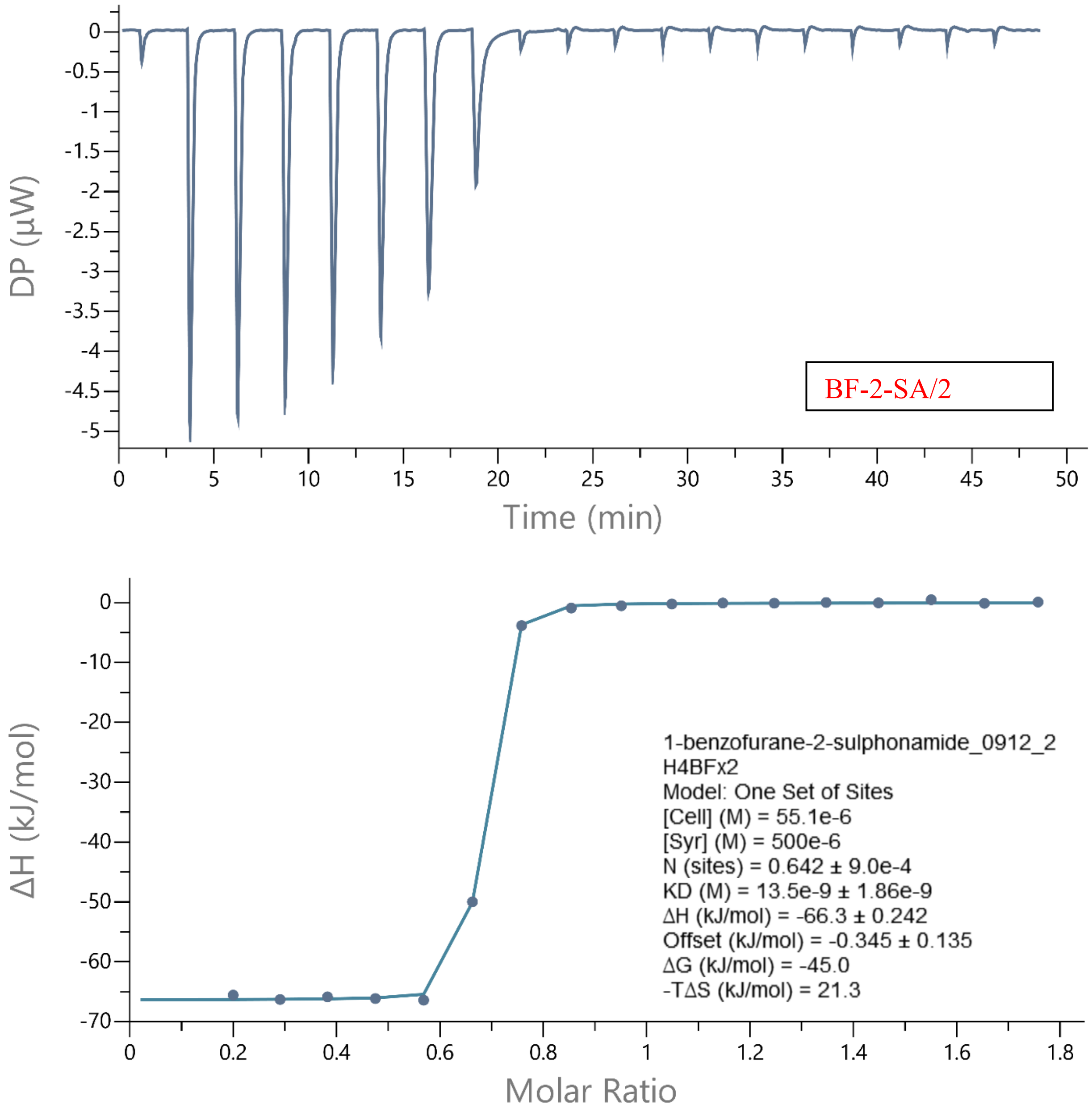

DP (μW)
Time (min)
BF-2-SA/2
ΔH (kJ/mol)
Molar Ratio
1-benzofurane-2-sulphonamide_0912_2
H4BFx2
Model: One Set of Sites
[Cell] (M) = 55.1e-6
[Syr] (M) = 500e-6
N (sites) = 0.642 ± 9.0e-4
KD (M) = 13.5e-9 ± 1.86e-9
ΔH (kJ/mol) = -66.3 ± 0.242
Offset (kJ/mol) = -0.345 ± 0.135
ΔG (kJ/mol) = -45.0
-TΔS (kJ/mol) = 21.3

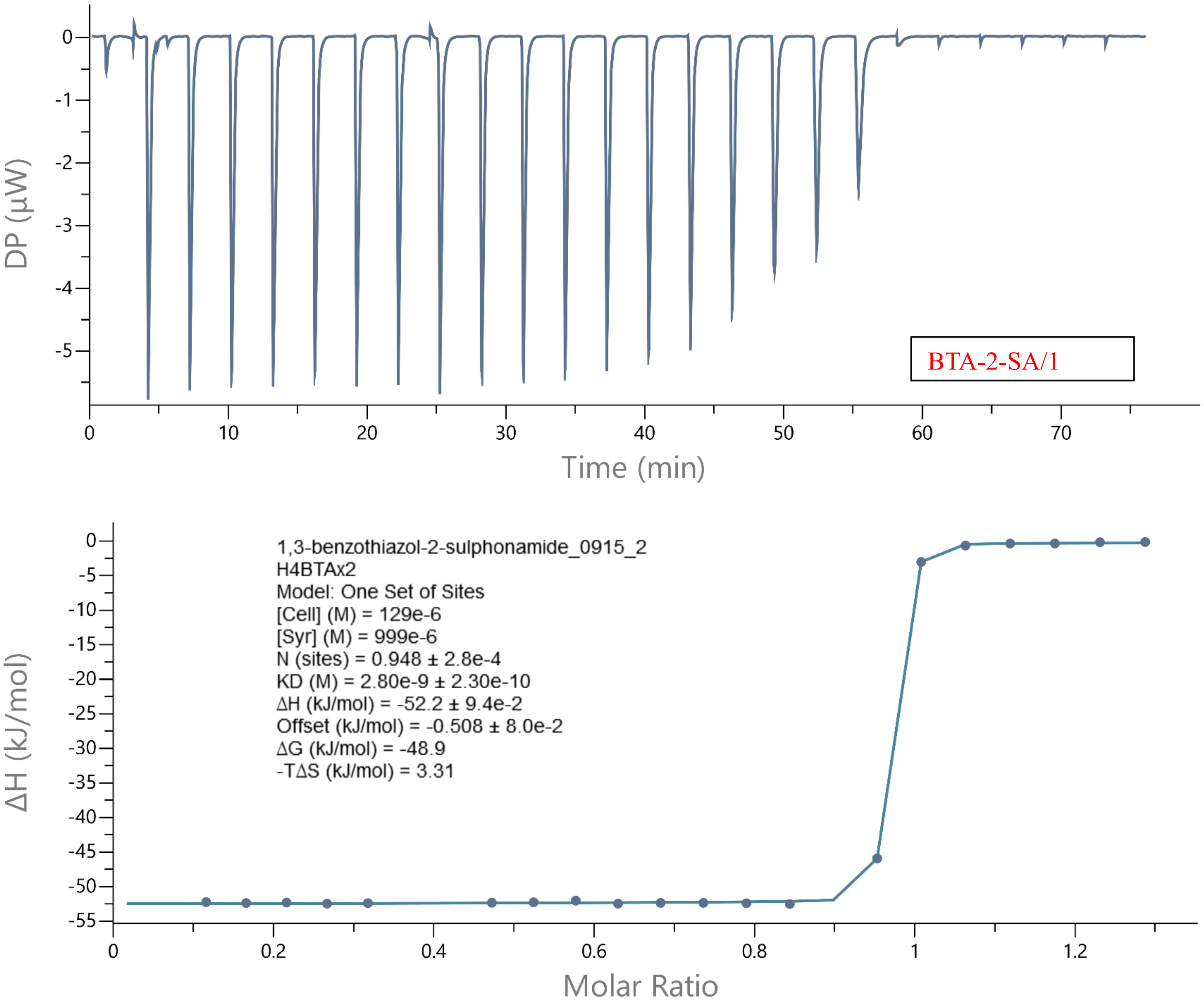

DP (μW)
Time (min)
BTA-2-SA/1
1,3-benzothiazol-2-sulphonamide_0915_2
H4BTAx2
Model: One Set of Sites
[Cell] (M) = 129e-6
[Syr] (M) = 999e-6
N (sites) = 0.948 ± 2.8e-4
KD (M) = 2.80e-9 ± 2.30e-10
ΔH (kJ/mol) = -52.2 ± 9.4e-2
Offset (kJ/mol) = -0.508 ± 8.0e-2
ΔG (kJ/mol) = -48.9
-TΔS (kJ/mol) = 3.31
ΔH (kJ/mol)
Molar Ratio

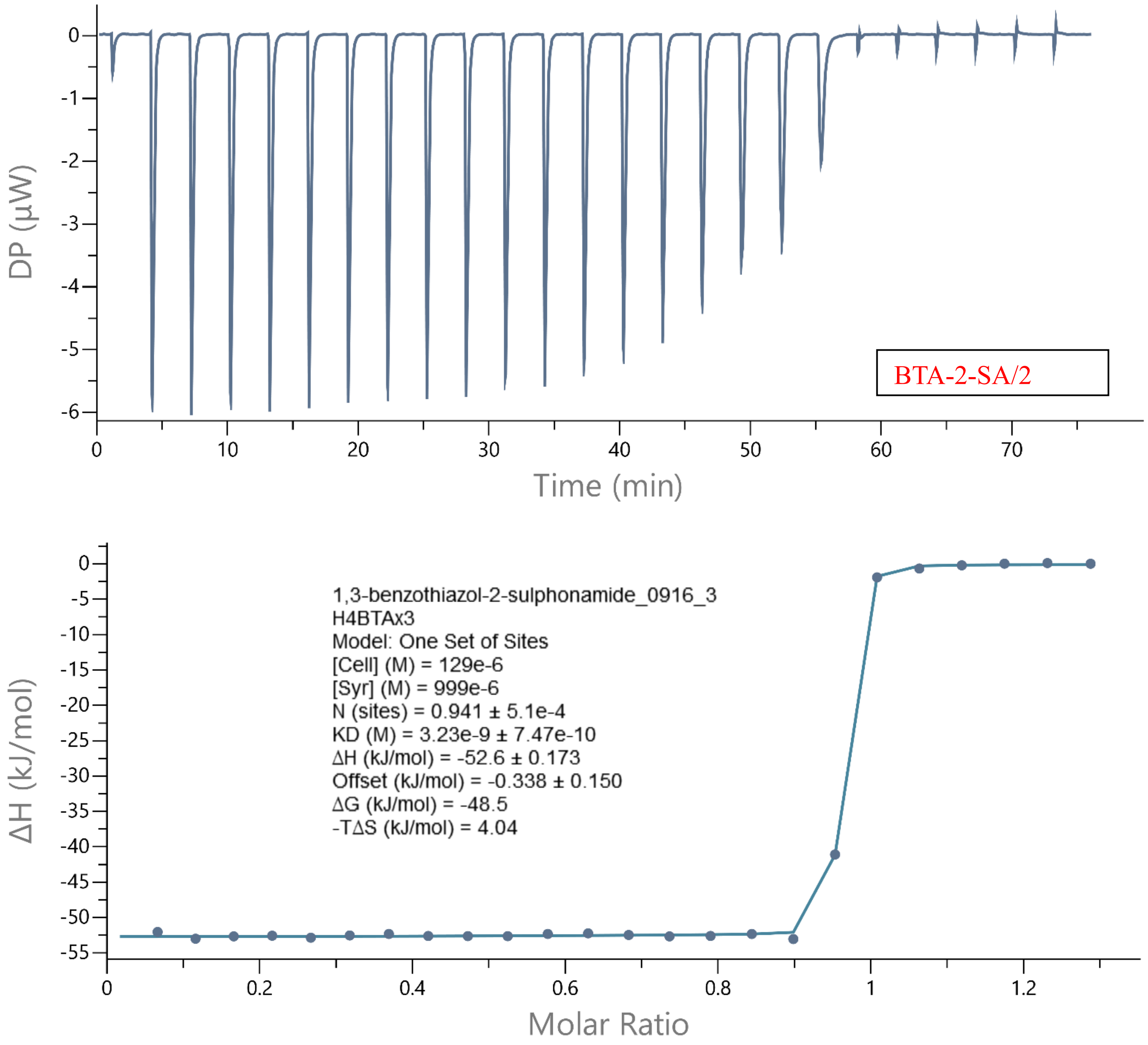

DP (μW)
Time (min)
BTA-2-SA/2
ΔH (kJ/mol)
Molar Ratio
1,3-benzothiazol-2-sulphonamide_0916_3
H4BTAx3
Model: One Set of Sites
[Cell] (M) = 129e-6
[Syr] (M) = 999e-6
N (sites) = 0.941 ± 5.1e-4
KD (M) = 3.23e-9 ± 7.47e-10
ΔH (kJ/mol) = -52.6 ± 0.173
Offset (kJ/mol) = -0.338 ± 0.150
ΔG (kJ/mol) = -48.5
-TΔS (kJ/mol) = 4.04

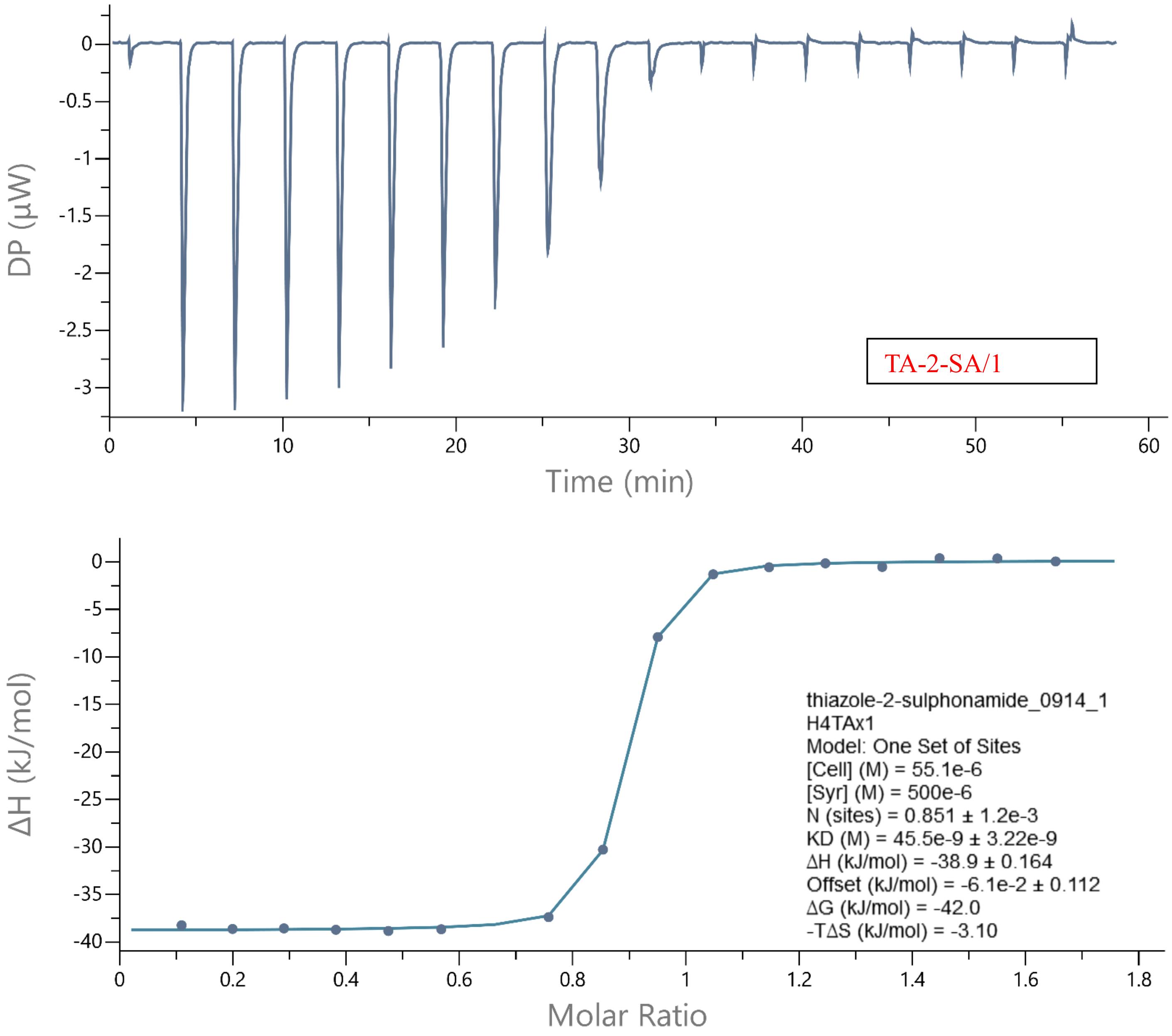
DP (μW)
Time (min)
TA-2-SA/1
ΔH (kJ/mol)
Molar Ratio
thiazole-2-sulphonamide_0914_1
H4TAx1
Model: One Set of Sites
[Cell] (M) = 55.1e-6
[Syr] (M) = 500e-6
N (sites) = 0.851 ± 1.2e-3
KD (M) = 45.5e-9 ± 3.22e-9
ΔH (kJ/mol) = -38.9 ± 0.164
Offset (kJ/mol) = -6.1e-2 ± 0.112
ΔG (kJ/mol) = -42.0
-TΔS (kJ/mol) = -3.10

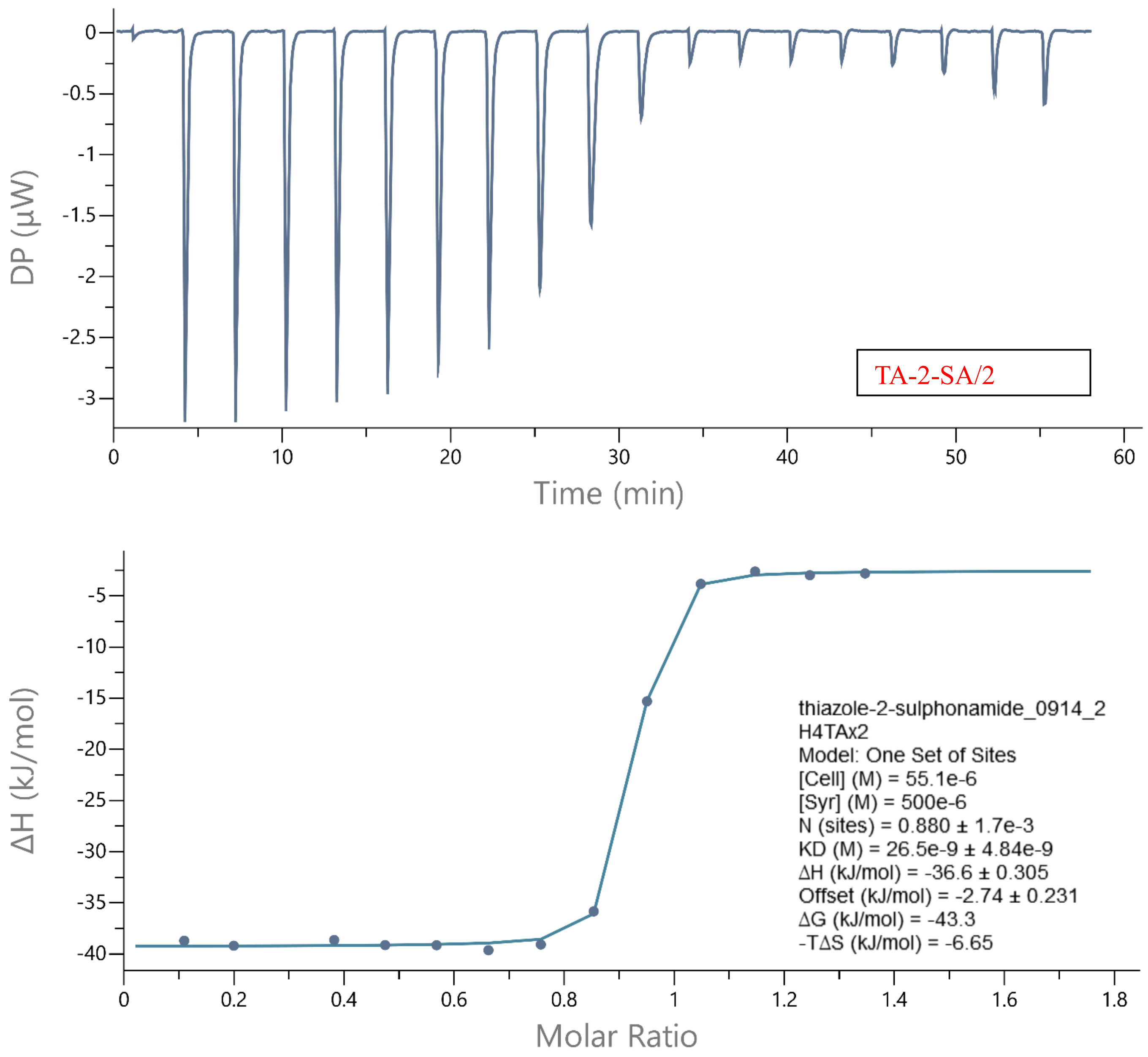

DP (μW)
Time (min)
TA-2-SA/2
ΔH (kJ/mol)
Molar Ratio
thiazole-2-sulphonamide_0914_2
H4TAx2
Model: One Set of Sites
[Cell] (M) = 55.1e-6
[Syr] (M) = 500e-6
N (sites) = 0.880 ± 1.7e-3
KD (M) = 26.5e-9 ± 4.84e-9
ΔH (kJ/mol) = -36.6 ± 0.305
Offset (kJ/mol) = -2.74 ± 0.231
ΔG (kJ/mol) = -43.3
-TΔS (kJ/mol) = -6.65

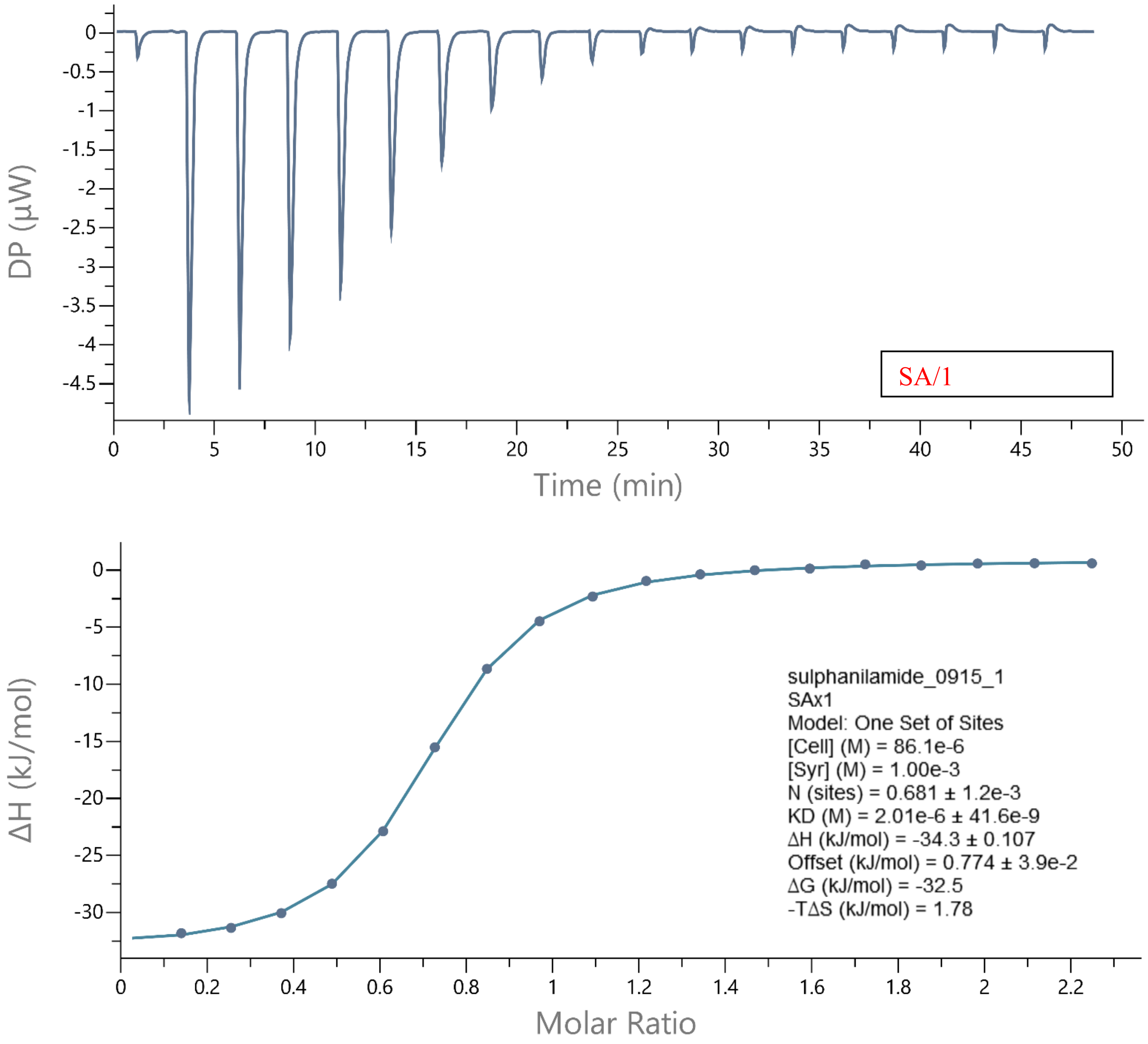

DP (μW)
Time (min)
SA/1
ΔH (kJ/mol)
Molar Ratio
sulphanilamide_0915_1
SAx1
Model: One Set of Sites
[Cell] (M) = 86.1e-6
[Syr] (M) = 1.00e-3
N (sites) = 0.681 ± 1.2e-3
KD (M) = 2.01e-6 ± 41.6e-9
ΔH (kJ/mol) = -34.3 ± 0.107
Offset (kJ/mol) = 0.774 ± 3.9e-2
ΔG (kJ/mol) = -32.5
-TΔS (kJ/mol) = 1.78

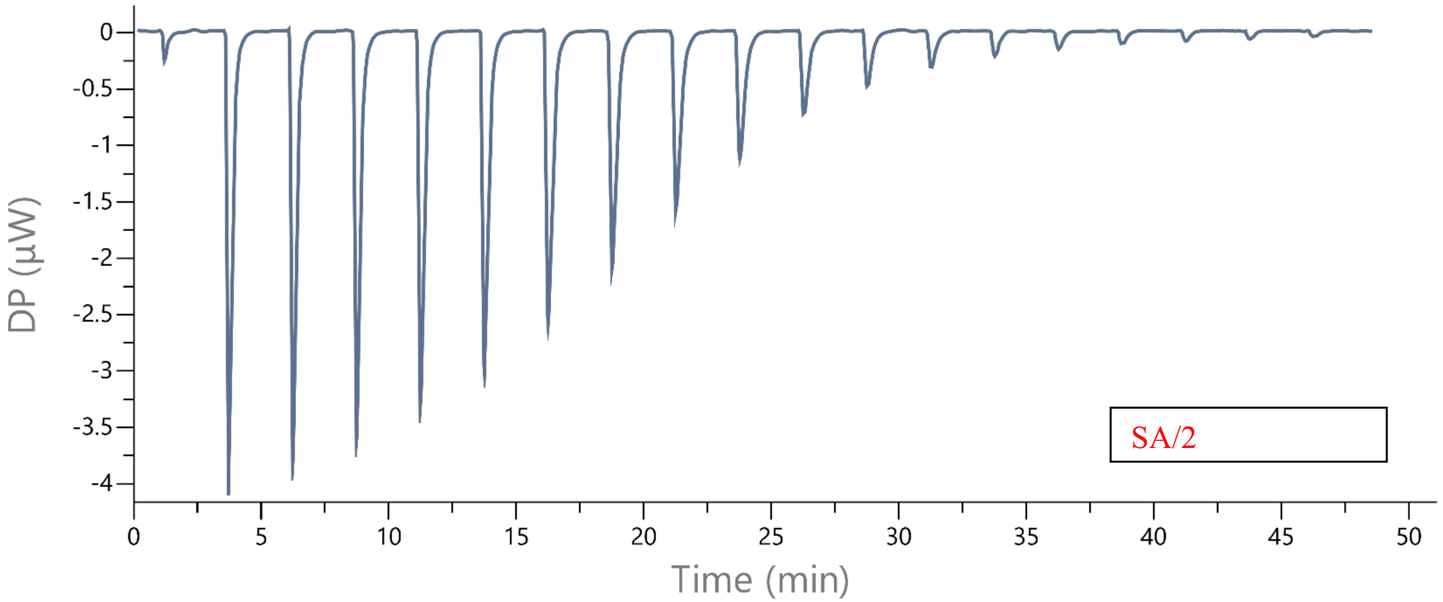

DP (μW)
Time (min)
SA/2

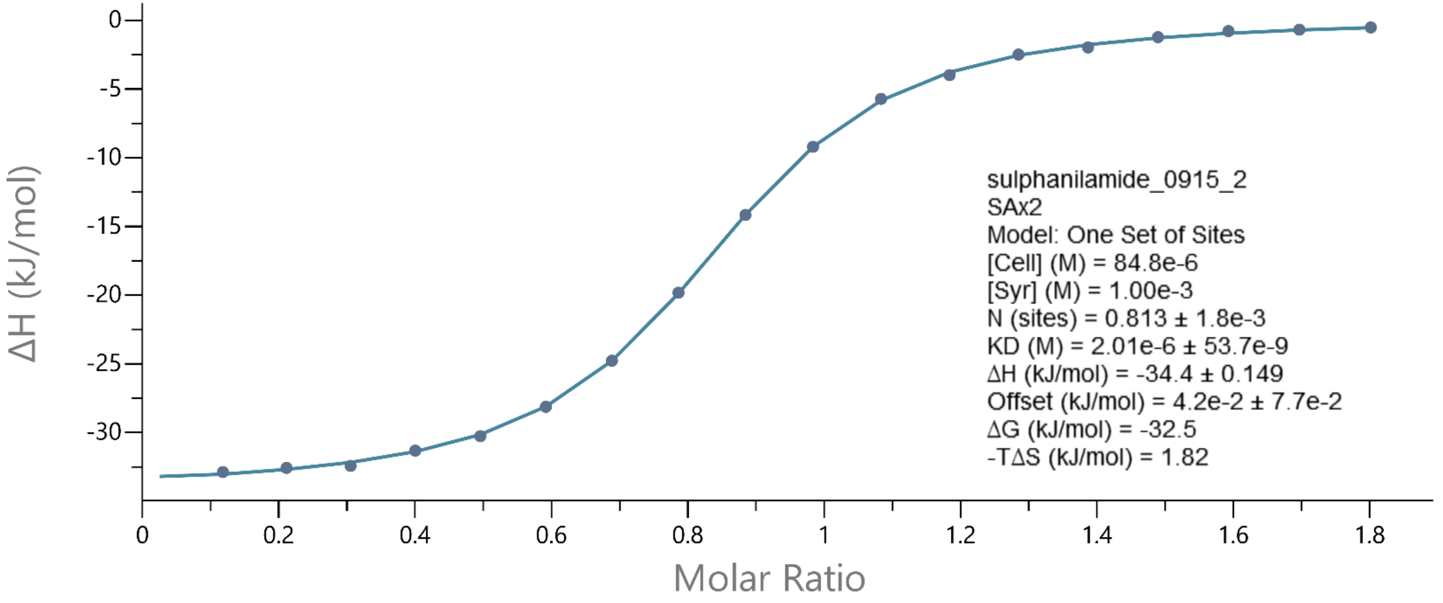

ΔH (kJ/mol)
Molar Ratio
sulphanilamide_0915_2
SAx2
Model: One Set of Sites
[Cell] (M) = 84.8e-6
[Syr] (M) = 1.00e-3
N (sites) = 0.813 ± 1.8e-3
KD (M) = 2.01e-6 ± 53.7e-9
ΔH (kJ/mol) = -34.4 ± 0.149
Offset (kJ/mol) = 4.2e-2 ± 7.7e-2
ΔG (kJ/mol) = -32.5
-TΔS (kJ/mol) = 1.82

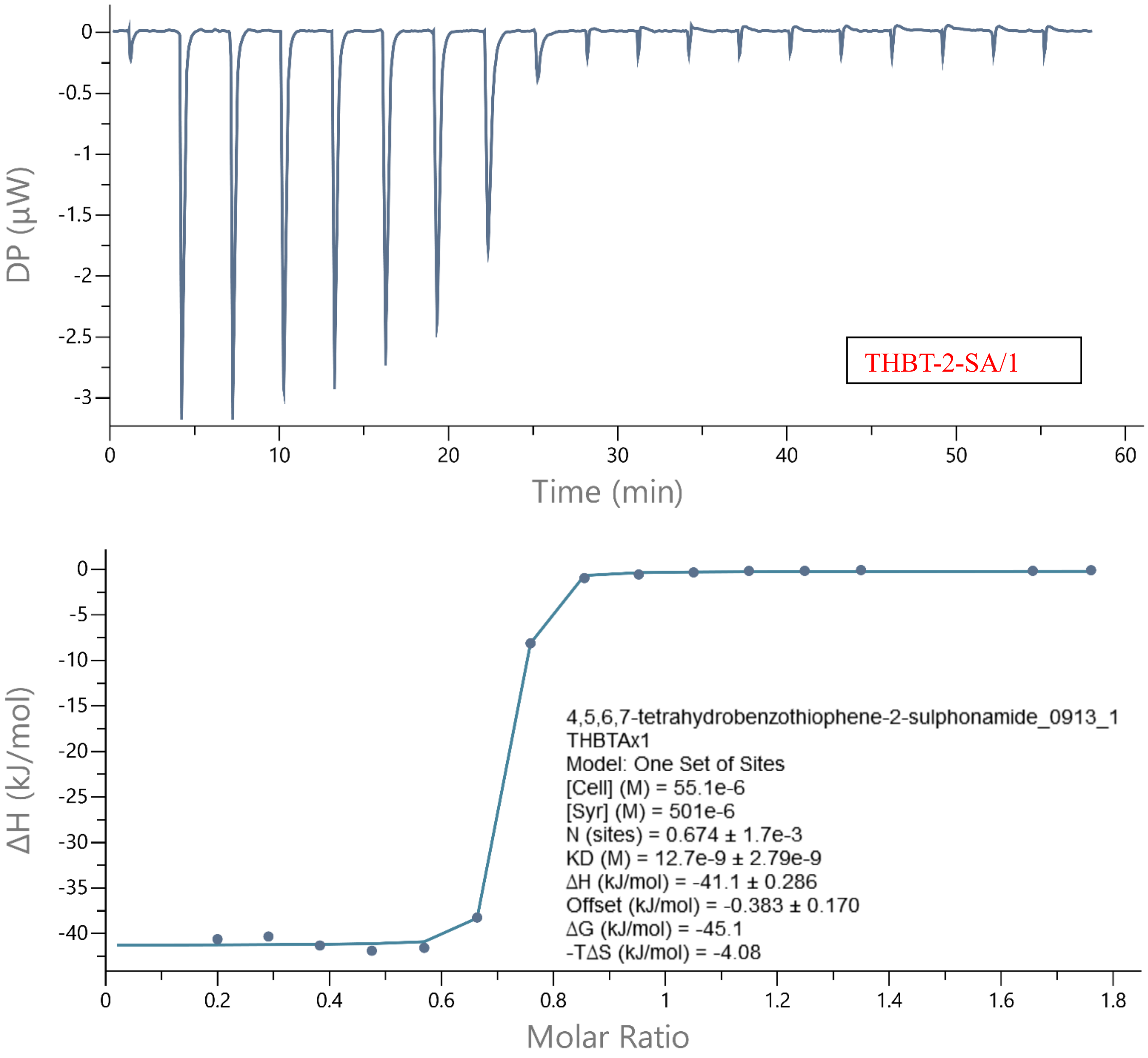

THBT-2-SA/1
DP (μW)
Time (min)
ΔH (kJ/mol)
Molar Ratio
4,5,6,7-tetrahydrobenzothiophene-2-sulphonamide_0913_1
THBTAx1
Model: One Set of Sites
[Cell] (M) = 55.1e-6
[Syr] (M) = 501e-6
N (sites) = 0.674 ± 1.7e-3
KD (M) = 12.7e-9 ± 2.79e-9
ΔH (kJ/mol) = -41.1 ± 0.286
Offset (kJ/mol) = -0.383 ± 0.170
ΔG (kJ/mol) = -45.1
-TΔS (kJ/mol) = -4.08

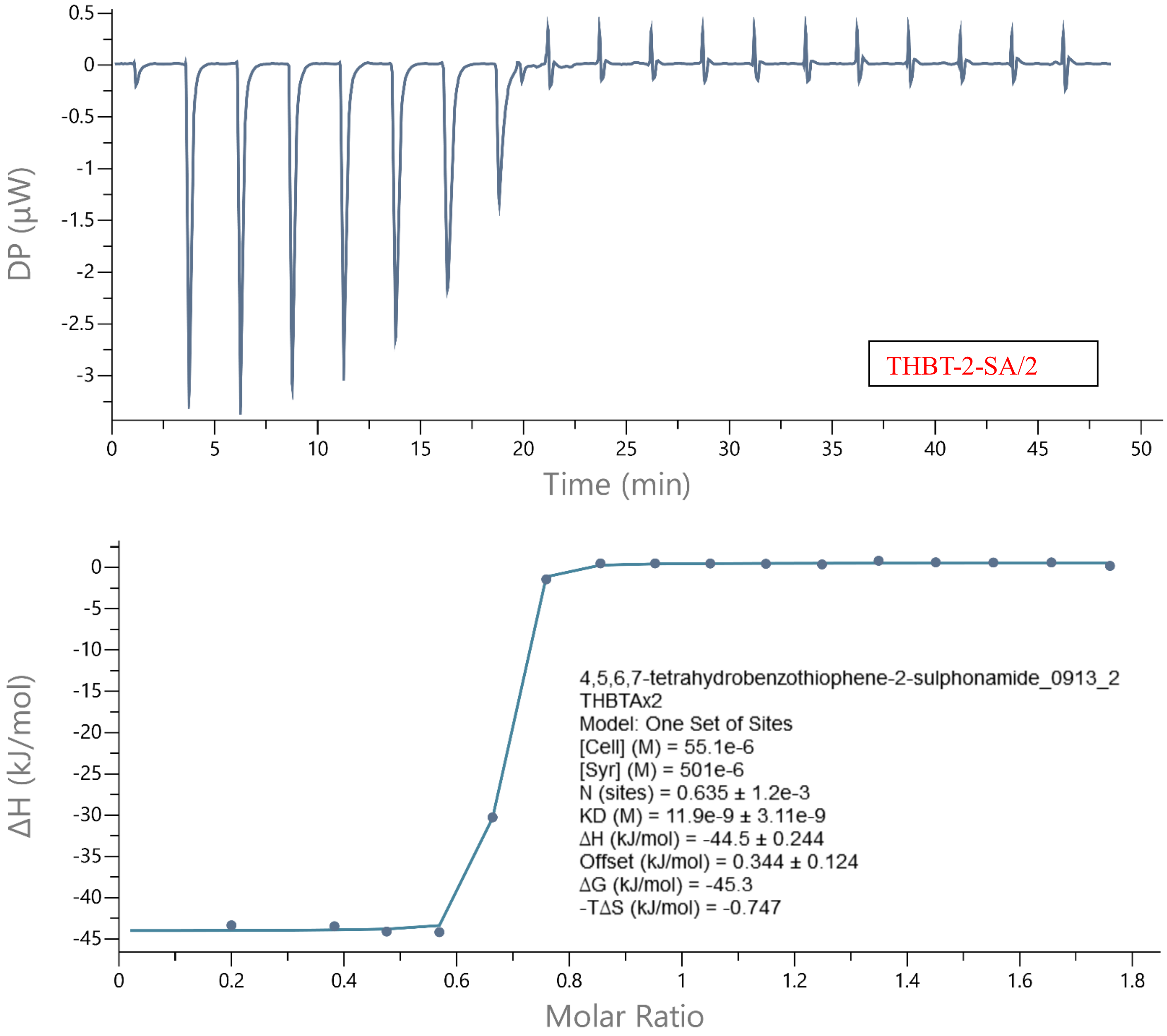
DP (μW)
Time (min)
THBT-2-SA/2
ΔH (kJ/mol)
Molar Ratio
4,5,6,7-tetrahydrobenzothiophene-2-sulphonamide_0913_2
THBTAx2
Model: One Set of Sites
[Cell] (M) = 55.1e-6
[Syr] (M) = 501e-6
N (sites) = 0.635 ± 1.2e-3
KD (M) = 11.9e-9 ± 3.11e-9
ΔH (kJ/mol) = -44.5 ± 0.244
Offset (kJ/mol) = 0.344 ± 0.124
ΔG (kJ/mol) = -45.3
-TΔS (kJ/mol) = -0.747

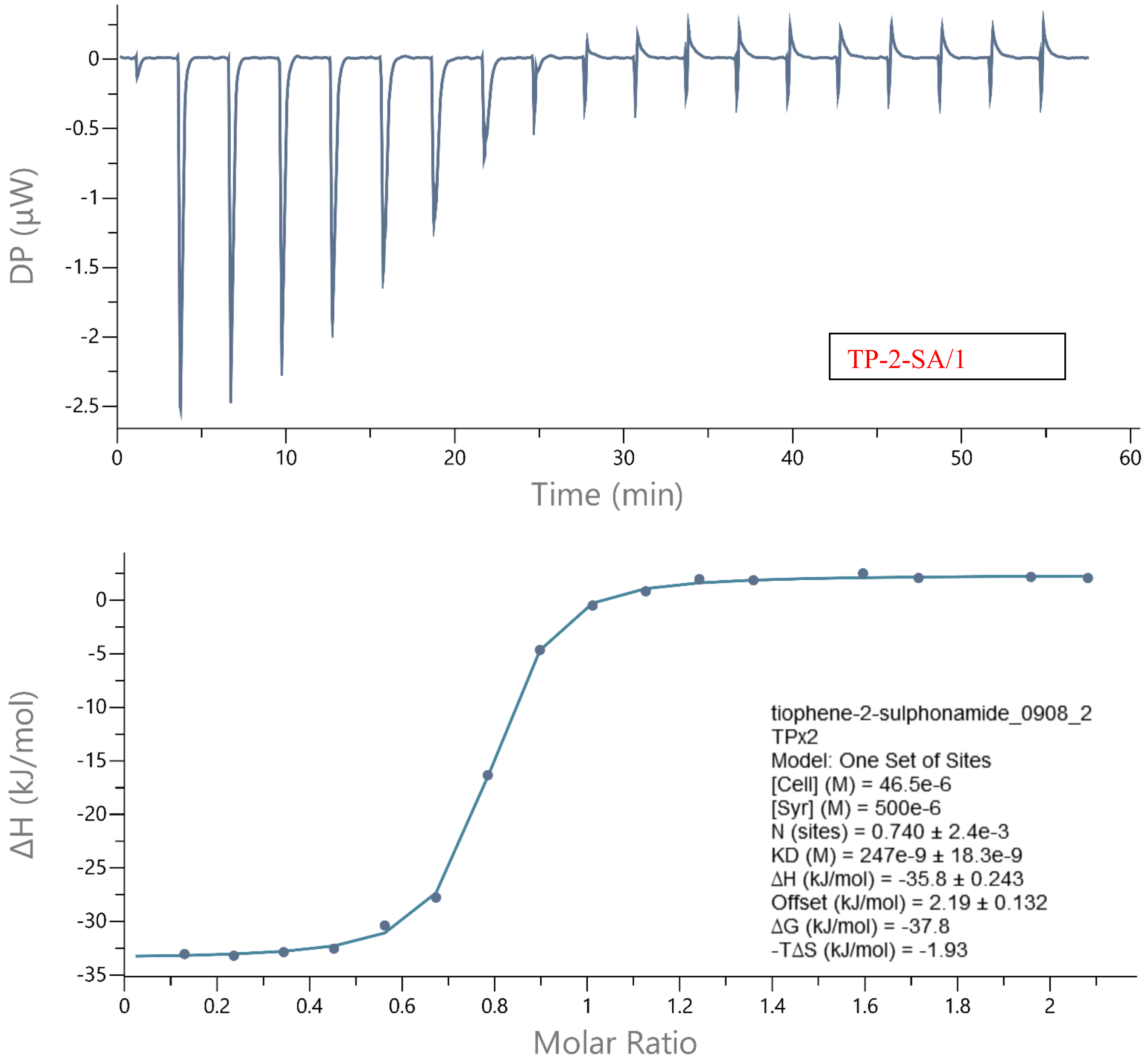
DP (μW)
Time (min)
TP-2-SA/1
ΔH (kJ/mol)
Molar Ratio
tiophene-2-sulphonamide_0908_2
TPx2
Model: One Set of Sites
[Cell] (M) = 46.5e-6
[Syr] (M) = 500e-6
N (sites) = 0.740 ± 2.4e-3
KD (M) = 247e-9 ± 18.3e-9
ΔH (kJ/mol) = -35.8 ± 0.243
Offset (kJ/mol) = 2.19 ± 0.132
ΔG (kJ/mol) = -37.8
-TΔS (kJ/mol) = -1.93

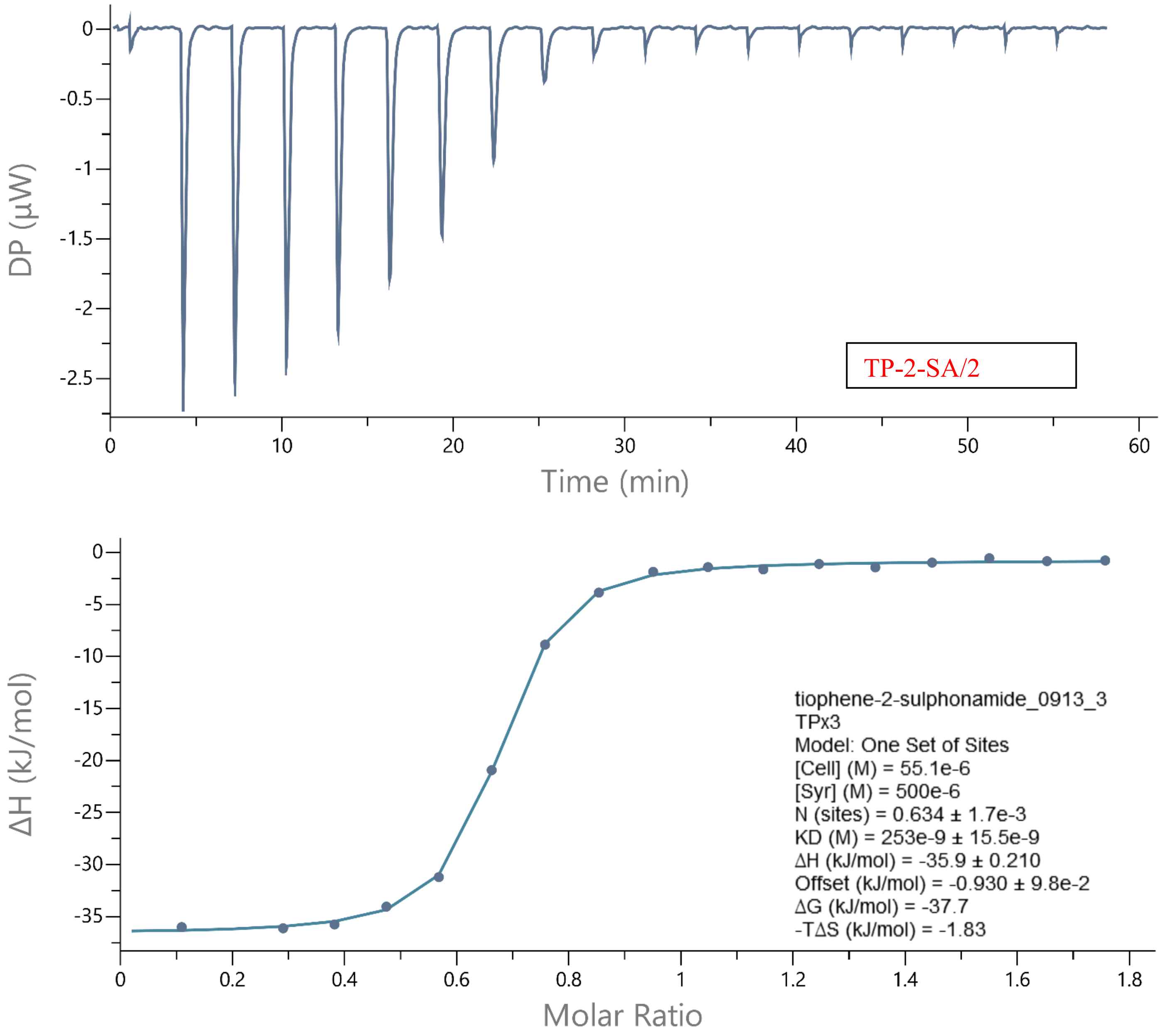
DP (μW)
0
-0.5
-1
-1.5
-2
-2.5
0
10
20
30
40
50
60
Time (min)
TP-2-SA/2
ΔH (kJ/mol)
0
-5
-10
-15
-20
-25
-30
-35
0
0.2
0.4
0.6
0.8
1
1.2
1.4
1.6
1.8
Molar Ratio
tiophene-2-sulphonamide_0913_3
TPx3
Model: One Set of Sites
[Cell] (M) = 55.1e-6
[Syr] (M) = 500e-6
N (sites) = 0.634 ± 1.7e-3
KD (M) = 253e-9 ± 15.5e-9
ΔH (kJ/mol) = -35.9 ± 0.210
Offset (kJ/mol) = -0.930 ± 9.8e-2
ΔG (kJ/mol) = -37.7
-TΔS (kJ/mol) = -1.83

4. References

1. Avvaru, B. S. *et al.* A short, strong hydrogen bond in the active site of human carbonic anhydrase II. *Biochemistry* **49**, 249–251 (2010).

2. Michalczyk, R. *et al.* Joint neutron crystallographic and NMR solution studies of Tyr residue ionization and hydrogen bonding: Implications for enzyme-mediated proton transfer. *Proc Natl Acad Sci U S A* **112**, 5673–5678 (2015).

3. Schiebel, J. *et al.* Intriguing role of water in protein-ligand binding studied by neutron crystallography on trypsin complexes. *Nature Communications 2018 9:1* **9**, 1–15 (2018).

4. Scott, A. D. *et al.* Thermodynamic Optimisation in Drug Discovery: A Case Study using Carbonic Anhydrase Inhibitors. *ChemMedChem* **4**, 1985–1989 (2009).

5. Glöckner, S., Ngo, K., Wagner, B., Heine, A. & Klebe, G. The Influence of Varying Fluorination Patterns on the Thermodynamics and Kinetics of Benzenesulfonamide Binding to Human Carbonic Anhydrase II. *Biomolecules 2020, Vol. 10, Page 509* **10**, 509 (2020).

6. Snyder, P. W. *et al.* Mechanism of the hydrophobic effect in the biomolecular recognition of arylsulfonamides by carbonic anhydrase. *Proc Natl Acad Sci U S A* **108**, 17889–17894 (2011).
